# Supplementary material for: Bertalanffy-Pütter models for avian growth
Source: PLoS One. 2021 Apr 26;16(4):e0250515. doi: 10.1371/journal.pone.0250515 (PMC8075225; doi:10.1371/journal.pone.0250515)
Supplement: Attachment — Submitted filename: PONE-D-20-31677 Review_SAO.pdf [file pone.0250515.s002.pdf]

Review of PONE-D-20-31677 Bertalanffy-Pütter models for avian growth

This paper presents an application of the von Bertalanffy-Pütter growth curve for analyzing differences in growth parameters (chiefly the position of the inflection point) for passerines. There has been much discussion through the years since the initial work of Ricklefs on the most suitable growth curve to use for different species and applications. The strength of this approach lies in the ability to alter m_{infl}/m_{max}, the position of the inflection point proportional to the extent of growth (most common parametric curves are limited to inflection occurring at a specific point in growth: e.g. 50% in the logistic model).

I find the approach appealing. I agree with the argument that a flexible m_{infl}/m_{max} ratio might have biological relevance in some instances of growth. I am also happy that curve-fitting was undertaken for each individual separately, as it is clear that the long tradition of pooling masses across individuals misses biologically important differences as it treats all individuals as equal.

I found the paper well written and easy to follow but hard to evaluate because the data, although analyzed per individual, were never directly presented in that form: Fig 2 presents individual data but it is not clear which belong to which individual and thus to gauge individual growth trajectories. Corsini et al. only report mass at age 15 – near fledging, and they also say that nestlings were ringed (banded) at age 15d. (This begs the question how could they identify individual chicks prior to that point to be able to collect growth on individuals?). It would be beneficial to know how nestlings were weighed and the accuracy of the instruments used to weigh them. I presume this will be rectified when data are deposited but it makes evaluation of the method harder.

My second concern is that the conclusion at the end of the paper: "as noted in the introduction, for many common three-parameter models of avian literature the ratio is fixed (e.g. ratio 0.5 for logistic growth). We conclude that these more traditional three-parameter models are not suitable to explore and compare the shapes of growth curves". I believe that this statement is too strong. Depending on the application of the growth curve a simpler curve may be just as appropriate and since it could be implemented in a well-developed analytical framework, such as mixed-effects models, it might be easier to use. I am very interested in the B-P approach but having to learn another program (Mathematica) just to implement it, is discouraging. From my experience, the majority of ornithologists use R for statistics and thus converting this code into an R package would greatly help foster its use by ornithologists.

One thing that is common is that as more parameters are estimated in a curve there is a greater potential for over-fitting, as changing one parameter can have a knock-on effect on another. I am familiar with this for the 4-parameter Richards curve and imagine that the B-P (4 or 5-parameters in eqn 1 in the paper) would suffer from the same issues. Thus, it is conceivable that different ratios of m_{infl}/m_{max} with different parameters for c, p and q might achieve similar shaped curves. This would imply that just because the ratio of m_{infl}/m_{max} has sufficiently different values for individuals or groups does not *necessarily* mean that it reflects the same differences in growth curve. I think what is needed is a figure (or even supplementary material) that shows there is consistency (or at least a limit to the range of variation) in curve shape across different ratios of m_{infl}/m_{max}. I also strongly believe that to truly show the B-P method is superior in the current application to two species of tits that the other 3-parameter curves (and/or Richards curve) need to be fit and compared to the B-P results. You could argue (perhaps by providing more details) that previous publications give support for the fact that B-P fit better than 3-

parameter curves and so this is not necessary. I would still suggest at this stage to play it safe and demonstrate this (even if just in supplementary material) for the data set at hand.

It would also be nice to provide an indication of approximately how many data points are needed for a good fit: with a 4-parameter Richards curve clearly 6+ is desirable. The need for a large number of data points well-spaced through development clearly precludes using the method in many cases where data are more irregularly collected.

Third, although stated in the 5th paragraph of the Introduction, the precise purpose of this paper [in how it differs from similar explorations for poultry (Kühleitner et al. 2019), poultry and dinosaurs (Brunner et al. 2019), and fish (Renner-Martin et al. 2018)] is currently not immediately forthcoming. I am interpreting it as: the need to show that differences in the ratio of m_{infl}/m_{max} have ecological implications. If this is the case, please state it more strongly in the abstract and introduction (and discussion) rather than focusing on the B-P as a superior method. I also believe that the paper would benefit from reference to some of the following ornithological literature on growth curves that is missing from the Introduction:

Brisbin Jr., I.L., C.T. Collins, G.C. White and D.A. McCallum. 1987. A new paradigm for the analysis and interpretation of growth data: The shape of things to come. Auk 104: 552–554.

Oswald, S.A., I.C.T. Nisbet, A. Chiaradia and J.M. Arnold. 2012. FlexParamCurve: R package for flexible fitting of nonlinear parametric curves. Methods in Ecology and Evolution 3: 1073–1077.

Remeŝ, V. and T.E. Martin. 2002. Environmental influences on the evolution of growth and developmental rates in passerines. Evolution 56: 2505–2518.

Sofaer, H.R., P.L. Chapman, T.S. Sillett and C.K. Ghalambor. 2013. Advantages of nonlinear mixed models for fitting avian growth curves. Journal of Avian Biology 44: 469–478.

Svagelj, W. S., Laich, A. G., & Quintana, F. (2019). Richards's equation and nonlinear mixed models applied to avian growth: why use them? Journal of Avian Biology, 50: e01864.

Zach, R. 1988. Growth-curve analysis: A critical reevaluation. Auk 105: 208–210.

Fourth, I find the "shotgun" style statistical treatment in "Associations between environmental and model parameters" a bit unusual. Remember, these hypothesis-testing statistics are testing the model parameters, not the data. Thus, prior to this type of approach, it should be verified that all individual models fit well and those for individuals that don't should be excluded. I know summary stats (and all values in the Supp. Material) are provided for the SSLE values but there is no indication of what SSLE value would constitute a good fit and what a poor fit in the present ms – I recommend including more detail on this, even though previously published elsewhere. If I were to square-root and backtransform SSLE, i.e. $e^{SQRT(SSLE)}$, would this equal the number of grams by which the curve deviated from the mass data across all measurements of an individual? I get a value between 1.03 and 1.5 across all individuals which seems like a very tight fit. It is my experience that some curves fit much worse than others, but this is not reflected here. Perhaps, it would help if the authors explained how the reader could gauge the fit of the model based on this value (a couple of plotted examples would help immensely).

Once poor-fitting models are excluded and we can be sure the m_{infl}/m_{max} ratio represents actual differences in shapes of growth curves (not just variation among parameter estimates – see my second

point above), then we can use this to identify ecological associations with the m_{infl}/m_{max} ratio. The approaches used here are not common for the ornithological literature for two reasons:

i. Multiple comparisons of hypothesis tests: for the types of studies common in ornithological literature, moderate sample sizes abound and thus, as you note, multiple comparisons lead to higher probability of spurious significance (this is different when considering large order comparisons as common in the genetics/microbiology literature – see Lazzeroni, L. C., & Ray, A. (2012). The cost of large numbers of hypothesis tests on power, effect size and sample size. Molecular psychiatry, 17(1), 108-114.).
ii. Multicollinearity: several predictor variables may be substantially correlated and so it is unclear the extent to which each is individually (or synergistically) important.

While I believe that with careful treatment of the multiple comparisons, it is possible to state as you do:

"In total, for each of the 11 environmental indicators we have applied six tests to each sample, resulting in 264 tests for significance (the entries in Tables 3 to 5). Amongst them, at most 19 outcomes would be spurious, recording a false significance (P-value 4.4% for a binomial distribution assuming a 5% chance for a false "significant"), while for our samples 61 outcomes were significant."

Clearly 61/264 significant outcomes is beyond the realm of pure chance at a 0.05 level. However, you cannot identify which of the 61 were the 19 spurious outcomes. I believe this means that you cannot further subdivide these results by saying:

"light pollution (11 of 24 tests were significant), followed by the impervious area (10 significant tests) and the count of hatchlings (9 significant tests)"

What if all 11 of the 24 tests of the light pollution predictor happened to be the spurious ones? Even if the chance of a spurious test was equal across all 264 tests (which given the various assumptions of the different tests and data themselves is unlikely), we would still expect 1 or 2 of the light pollution (19/264 x 24) tests to be spurious. I don't believe you can look any deeper with this analytical approach than saying that overall there are 3 times as many significant associations at the 0.05 level than would be expected by chance.

This supports your statement:

"We conclude that despite the small sample size the BP-model could detect significant statistical association between some local environmental variables and certain shape parameters of the BP-model, namely the ratio minfl/mmax and the exponent-difference b–a."

but renders the details presented in Tables 3-5 misleading and unreliable.

As for (ii) above, I imagine a correlation matrix across the 11 environmental variables would reveal strong associations (e.g. path vs human activity, sound vs road, etc..) and thus it is unclear which are important without adjustment for this. Moreover, this suggests that the 264 significance tests aren't fully independent which would affect the likely rate of a spurious significance test. Finally, a related point is – how were nest-mates used in the analysis? If you have two chicks from the same nest box they are certainly not independent and thus have the potential to have more influence within the analytical group in which they fall.

Finally, I believe the paper could be a bit easier to follow if revisions were made to the Tables and Figures. I found all the tables to be too detailed for the reader to get an immediate idea of what is important in each. These types of tables belong in supplementary data, with a figure or short table in the text. Table 1 could go in supplementary info. If the concerns about significance testing given above can be addressed, Tables 3 &4 could somehow be incorporated into Table 2 – thus the significance is reported using * = p< 0.05 etc.. and the medians from Table 4 could even be plotted in a figure. I certainly found it hard going to get much out of the current tabular presentation. I would also remove Figure 1. As indicated above it would be nice to see actual individuals labelled in Figure 2, even if fewer were plotted. Figure 3 could be presented better – since this is one specific example, couldn't the mass data points be plotted with the curves? Even better would be a multipanel figure showing several fits. In Fig 4, the near-diagonal exponent pair stratification is an interesting one. Can you expand in the discussion about what this means (what are the physiological implications of having a near-diagonal exponent pair in the B-P curve)?

I thank you for the opportunity to review this interesting work. I tried to be as detailed in my comments as I could as I believe this method has much potential. However, please feel free to contact me if you have any further questions. I have also provided minor textual suggestions as comments in the ms I attached.

Steve Oswald (sao10@psu.edu)
