# Supplementary material for: Bertalanffy-Pütter models for avian growth
Source: PLoS One. 2021 Apr 26;16(4):e0250515. doi: 10.1371/journal.pone.0250515 (PMC8075225; doi:10.1371/journal.pone.0250515)
Supplement: Attachment — Submitted filename: PONE-D-20-31677_reviewer.pdf [file pone.0250515.s003.pdf]

PLOS ONE

Bertalanffy-Pütter models for avian growth

--Manuscript Draft--

**Manuscript Number:**	PONE-D-20-31677
**Article Type:**	Research Article
**Full Title:**	Bertalanffy-Pütter models for avian growth
**Short Title:**	Models for avian growth
**Corresponding Author:**	Manfred Kühleitner<br>University of natural resources and life sciences<br>Vienna, AUSTRIA
**Keywords:**	passerine birds; Cyanistes caeruleus; Parus major,metabolic exponents; life history; Growth model; Bertalanffy-Pütter differential equation; simulated annealing
**Abstract:**	The logistic model is widely used in avian growth literature, but it is inflexible with respect to the shape of the growth curve. For instance, it assumes a constant ratio 0.5 of the size of the inflection point over the asymptotic size. The Bertalanffy-Pütter (BP) model is more versatile in this respect, as it has two additional parameters. We explored whether certain shape-parameters for the growth curves of the BP-model (e.g. the above-mentioned ratio) could be used to detect life history impacts from environmental variation. To this end we used literature data for the growth of 81 nestlings of blue tits (BT) and great tits (GT) from nestlings from an urban parkland in Warsaw, Poland. We identified the best-fit BP-models to these data and found significant associations between the shape-parameters (computed from the best-fit models) and the environmental condition at the nests.
**Order of Authors:**	Manfred Kühleitner
	Norbert Brunner
**Additional Information:**	
**Question**	**Response**
**Financial Disclosure**<br><br>Enter a financial disclosure statement that describes the sources of funding for the work included in this submission. Review the submission guidelines for detailed requirements. View published research articles from *PLOS ONE* for specific examples.<br><br>This statement is required for submission and **will appear in the published article** if the submission is accepted. Please make sure it is accurate.	The author(s) received no specific funding for this work.

Powered by Editorial Manager® and ProduXion Manager® from Aries Systems Corporation

**Unfunded studies**<br>Enter: *The author(s) received no specific funding for this work.*<br><br>**Funded studies**<br>Enter a statement with the following details:<br>• Initials of the authors who received each award<br>• Grant numbers awarded to each author<br>• The full name of each funder<br>• URL of each funder website<br>• Did the sponsors or funders play any role in the study design, data collection and analysis, decision to publish, or preparation of the manuscript?<br>• **NO** - Include this sentence at the end of your statement: *The funders had no role in study design, data collection and analysis, decision to publish, or preparation of the manuscript.*<br>• **YES** - Specify the role(s) played.<br><br>* typeset	
**Competing Interests**<br><br>Use the instructions below to enter a competing interest statement for this submission. On behalf of all authors, disclose any competing interests that could be perceived to bias this work—acknowledging all financial support and any other relevant financial or non-financial competing interests.<br><br>This statement **will appear in the published article** if the submission is accepted. Please make sure it is accurate. View published research articles from *PLOS ONE* for specific examples.	The authors have declared that no competing interests exist.

**NO authors have competing interests**<br><br>Enter: *The authors have declared that no competing interests exist.*<br><br>**Authors with competing interests**<br><br>Enter competing interest details beginning with this statement:<br><br>*I have read the journal's policy and the authors of this manuscript have the following competing interests: [insert competing interests here]*<br><br>* typeset	
**Ethics Statement**<br><br>Enter an ethics statement for this submission. This statement is required if the study involved:<br><br>• Human participants<br>• Human specimens or tissue<br>• Vertebrate animals or cephalopods<br>• Vertebrate embryos or tissues<br>• Field research<br><br>Write "N/A" if the submission does not require an ethics statement.<br><br>General guidance is provided below. Consult the submission guidelines for detailed instructions. **Make sure that all information entered here is included in the Methods section of the manuscript.**	N/A

**Format for specific study types**<br><br>**Human Subject Research (involving human participants and/or tissue)**<br>• Give the name of the institutional review board or ethics committee that approved the study<br>• Include the approval number and/or a statement indicating approval of this research<br>• Indicate the form of consent obtained (written/oral) or the reason that consent was not obtained (e.g. the data were analyzed anonymously)<br><br>**Animal Research (involving vertebrate animals, embryos or tissues)**<br>• Provide the name of the Institutional Animal Care and Use Committee (IACUC) or other relevant ethics board that reviewed the study protocol, and indicate whether they approved this research or granted a formal waiver of ethical approval<br>• Include an approval number if one was obtained<br>• If the study involved *non-human primates*, add *additional details* about animal welfare and steps taken to ameliorate suffering<br>• If anesthesia, euthanasia, or any kind of animal sacrifice is part of the study, include briefly which substances and/or methods were applied<br><br>**Field Research**<br><br>Include the following details if this study involves the collection of plant, animal, or other materials from a natural setting:<br>• Field permit number<br>• Name of the institution or relevant body that granted permission	
**Data Availability**<br><br>Authors are required to make all data underlying the findings described fully available, without restriction, and from the time of publication. PLOS allows rare exceptions to address legal and ethical concerns. See the PLOS Data Policy and FAQ for detailed information.	No - some restrictions will apply

A Data Availability Statement describing where the data can be found is required at submission. Your answers to this question constitute the Data Availability Statement and **will be published in the article**, if accepted.<br><br>**Important:** Stating 'data available on request from the author' is not sufficient. If your data are only available upon request, select 'No' for the first question and explain your exceptional situation in the text box.<br><br>Do the authors confirm that all data underlying the findings described in their manuscript are fully available without restriction?	
**Describe where the data may be found in full sentences. If you are copying our sample text, replace any instances of XXX with the appropriate details.**<br><br>• If the data are **held or will be held in a public repository**, include URLs, accession numbers or DOIs. If this information will only be available after acceptance, indicate this by ticking the box below. For example: *All XXX files are available from the XXX database (accession number(s) XXX, XXX.*).<br>• If the data are all contained **within the manuscript and/or Supporting Information files**, enter the following: *All relevant data are within the manuscript and its Supporting Information files.*<br>• If neither of these applies but you are able to provide **details of access elsewhere**, with or without limitations, please do so. For example:<br><br>*Data cannot be shared publicly because of [XXX]. Data are available from the XXX Institutional Data Access / Ethics Committee (contact via XXX) for researchers who meet the criteria for access to confidential data.*<br><br>*The data underlying the results presented in the study are available from (include the name of the third party*	The data underlying the results presented in the study are available from Corsini et al. under the following links:<br><br>https://www.frontiersin.org/article/10.3389/fevo.2017.00082/full#supplementary-material<br><br>https://onlinelibrary.wiley.com/action/downloadSupplement?doi=10.1111%2Feva.13081&file=eva13081-sup-0001-Supinfo.docx

and contact information or URL).<br>• This text is appropriate if the data are owned by a third party and authors do not have permission to share the data.<br><br>* typeset	
Additional data availability information:	

Manuscript

Click here to access/download;Manuscript;GrowthBirdsFinal.docx

Title: Bertalanffy-Pütter models for avian growth

Short title: Models for avian growth

Authors: Norbert Brunner, Manfred Kühleitner*, and Katharina Renner-Martin

Affiliation of all authors:

University of Natural Resources and Life Sciences (BOKU),

Department of Integrative Biology and Biodiversity Research (DIBB),

A-1180 Vienna, Austria

E-mail of all authors:

norbert.brunner@boku.ac.at, manfred.kuehleitner@boku.ac.at, kathi.renner-martin@gmx.de

ORCID of authors:

NB: 0000-0001-6487-5752, MK: 0000-0002-0217-7550, KRM: 0000-0002-5733-2546

* **Corresponding author:** Manfred Kühleitner

Institute of Mathematics, DIBB, BOKU

Gregor Mendel Strasse 33, A-1180 Vienna, Austria

E-Mail: manfred.kuehleitner@boku.ac.at

Bertalanffy-Pütter models for avian growth

Abstract. The logistic model is widely used in avian growth literature, but it is inflexible with respect to the shape of the growth curve. For instance, it assumes a constant ratio 0.5 of the size of the inflection point over the asymptotic size. The Bertalanffy-Pütter (BP) model is more versatile in this respect, as it has two additional parameters. We explored whether certain shape-parameters for the growth curves of the BP-model (e.g. the above-mentioned ratio) could be used to detect life history impacts from environmental variation. To this end we used literature data for the growth of 81 nestlings of blue tits (BT) and great tits (GT) from nestlings from an urban parkland in Warsaw, Poland. We identified the best-fit BP-models to these data and found significant associations between the shape-parameters (computed from the best-fit models) and the environmental condition at the nests.

Key words: passerine birds, Cyanistes caeruleus, Parus major,metabolic exponents, life history, growth model, Bertalanffy-Pütter differential equation, simulated annealing

Abbreviations: *BP model*: Bertalanffy-Pütter model; *BT*: blue tits; *F*: female; *GT*: great tits; *M* male; *SSLE*: sum of squared logarithmically transformed errors

Introduction

This paper uses nonlinear regression models to study the growth of passerine birds. In temporal order, the most common three-parameter growth models in avian research (Kuhi et al., 2010) are the Gompertz (1832) model, the logistic model of Verhulst (1838), the monomolecular model (bounded exponential growth) of Brody (1945), the von Bertalanffy (1949) model, and the more recent model by West et al. (2001). There are also simpler trend models, such as power-laws between size and age (Katsanevakis & Maravelias, 2008), and more complex models aiming at explaining growth in relation to e.g. food consumption (Manabe et al., 2018).

Ricklefs (1967) was amongst the first to recommend simple regression models for exploring the growth patterns of nestlings. For example, the logistic model was used to relate growth to predation (Ricklefs, 1984), to annual adult mortality rates and to incubation period duration (Remeš, 2007), and to environmental variation (Bleu et al., 2017; Ndithia et al., 2017; Remacha et al., 2016). Thereby, various authors considered e.g. tree cover (Kirschel et al., 2009), pollution by light and sound (Da Silva et al, 2014; Francis, 2015; Halfwerk et al, 2011) or nest interference by humans and pets (Gladalski et al., 2016). All these factors are known to affect the breeding success of birds, too. When clearly distinct environments were compared, e.g. forests and urban parklands, then significant differences in the breeding success of BT (Gladalski et al., 2016) and in the growth of BT-nestlings (Remacha et al., 2016) could be established by logistic growth and other simple models. In this paper we aimed at demonstrating that less distinct environmental variation and the ongoing adaption of birds to urban environments (Bańbura & Bańbura, 2012) at a small scale could potentially be detected with more complex models.

There are many different types of complex growth models, such as (incomplete list) spline-interpolation (Aggrey, 2002; Maceina, 2007), GARCH and similar time series methods (Hsing, 2005), stochastic (partial) differential equations (Garcia, 1983; Jacobs et al., 2019) or mixed-effect models (Pinheiro & Bates, 2000; Serroyenet al., 2009; Strathe et al., 2010). We decided to use the five-parameter Bertalanffy-Pütter regression model (BP-model) because it generalizes the above-mentioned commonly used three-parameter regression models and because it provides a common biophysical interpretation (see below) for them. The growth function $m(t)$ of the BP-model describes mass (m) at time (t) using the following differential equation (Pütter, 1920). It can be solved analytically, but in general not by means of elementary functions (Ohnishi et al., 2014).

$$(1) \quad m'(t) = p \cdot m(t)^a - q \cdot m(t)^b$$

The model parameters of equation (1) are to be determined from fitting the model to mass-at-age data: Four parameters are displayed in the equation, namely the non-negative exponent-pair $a < b$ (literature refers to them as "metabolic exponents") and the constants p and q. An additional parameter is the initial value (intuitively the hatching mass), i.e. $m(0) = c > 0$. Each exponent-pair (a, b) defines a distinct model $BP(a, b)$ with three parameters (c, p and q) as a special case: The above-mentioned models of Bertalanffy and West are $BP(2/3, 1)$ and $BP(3/4, 1)$, respectively, monomolecular growth is $BP(0, 1)$, and logistic growth is $BP(1, 2)$. The Gompertz model is the limit case $BP(1, 1)$ with a distinct differential equation, where b converges to $a = 1$ from above (Marusic & Bajzer, 1993).

Bertalanffy (1957) interpreted equation (1) as a model of ontogenetic growth, where the body would utilize resources at a metabolic rate ($p \cdot m^a$) for growth, except for the resources allocated to the operation and maintenance of existing tissue ($q \cdot m^b$). Different biophysical explanations for growth translated into different metabolic exponents. Thus, Bertalanffy (1949) proposed the exponents $a = 2/3$, as lung surface (assumed to be proportional to the 2/3th power of mass) would delimit oxygen uptake, and $b = 1$, as cell count (assumed to be proportional to mass) would determine the resources needed for maintenance. West et al. (2004) developed a different argument in support of the exponents $a = 3/4$ and $b = 1$ for modeling avian growth. Other authors stressed that the metabolic exponents would also depend on the environment and not merely on biophysics (e.g. Stewart et al., 2013; White, 2010), whence it would be meaningful to identify the best-fit exponent-pair.

In previous studies of animal growth, the BP-model (1) achieved significant improvements over e.g. the logistic model with respect to the fit of the data (e.g. Brunner et al., 2019; Renner-Martin et al., 2018 and 2019; Pauly & Cheung, 2018), but the benefits of its use remained little known and insufficiently evaluated. A major concern about simple three-parameter models was the inflexibility with respect to the shape of the growth curve (Tjørve & Tjørve, 2010). Thereby, a main advantage of using additional parameters was the added flexibility for estimating the location of the inflection point in relation to asymptotic size. For solutions of equation (1), the asymptotic mass m_{max} and the mass m_{infl} at the inflection point are computed from equation (2). For $q = 0$ asymptotic mass is infinite and for $a = 0$ there is no inflection point. Further, other than for the above-mentioned three-parameter models (Tjørve & Tjørve, 2017), for the general model (1) the age t_{infl} at the inflection point could only be determined numerically (solving $m(t) = m_{infl}$ for t).

$$(2) \quad m_{max} = \left(\frac{p}{q}\right)^{\frac{1}{b-a}}, \quad m_{infl} = \left(\frac{a}{b}\right)^{\frac{1}{b-a}} \cdot m_{max}$$

Both parameters have a biological meaning. The maximal growth rate, i.e. the derivative $m'(t_{infl})$, was suggested as a proxy for the basal metabolic rate (Calder, 1985). Further, the inflection point was related to a change in diet, from spiders with much keratin (for the growth of feathers) to protein-rich caterpillars (Naef-Daenzer & Keller, 1999). Asymptotic mass was often used to estimate the adult mass. However, we did not use this extrapolation, as after fledging and prior to attaining adult mass there might have occurred another growth phase, which we could not observe for wild birds (Grossmann, 1988.)

By equation (2), for logistic growth the size at the inflection point is always half of the asymptotic size (similar relations hold for the other above-mentioned three-parameter models), while the general model (1) is flexible. (It allows to realize any ratio between 0 and 1.) In this paper we argue that the ratio m_{infl}/m_{max} may have a biological meaning, whence models that are flexible with respect to this ratio would be preferable. To this end we used data from Corsini et al. (2020) on the development of 81 nestlings of blue tits (BT: *Cyanistes caeruleus*) and great tits (GT: *Parus major*) that grew up in a park of the Warsaw city center. For each nestling we identified the best-fit parameters for the BP-model to compute, for each bird, the individual ratio m_{infl}/m_{max}. (This approach differed from the above-mentioned papers that fitted one model to the aggregate data, namely average-size-at-age, of many animals.) For further statistical analysis we used environmental data from Szulkin et al. (2020) to explore whether the ratio and other parameters derived from the BP-model could reveal impacts of the environment on avian growth that were unobserved with other models.

Methods

Bird and nest-box data

This paper is based on life history data from Corsini et al. (2020) and environmental data from Szulkin et al. (2020) that were collected between March and June of 2016. We received from these authors data about 429 nestlings from 56 nest-boxes at various study sites in and around Warsaw, Poland. For each bird, data included the ID of the bird (ring number) and of the nest-box, the species (BT or GT), the hatching date, the number of hatchlings, mass in gram (eight repeated weighing), sex (F or M), and fledging success.

To ensure comparability, we studied only a fraction of the 429 nestlings. First, to exclude irregular growth patterns from runts, we selected only birds whose sex could be determined (F or M) and that finally became fledged. (We used these criteria as a proxy for healthy birds.) Second, we selected only birds that were weighed at the same eight odd numbered days 1 (hatching), 3, 5, 7, … 15. We thereby considered only nestlings that survived that long (the others were runts or predated by woodpeckers or martens). As we wished to compare the shapes of the growth curves, we did not consider birds that were weighed at other days, even if they survived. Third, we selected only hatchlings from the first brood. They grew up during May and June; the month of May was dry with cool nights and June was warm and humid (Figure 1). This reduced the count of nestlings to 95 birds in 24 nests at two study sites: Kampinos national park (4 nest-boxes with 14 nestlings) and Pole Mokotowskie park close to the city center (20 nest-boxes with 81 nestlings). Fourth, for a statistical comparison of growth in relation to the environment, we removed the Kampinos data. Otherwise, a rural-urban comparison would depend on merely four rural nests. Further, as for our data rural hatching occurred later, it was likely that food availability and composition differed, but there were no data about the diet.

Thus, our study was based on 81 Pole Mokotowskie nestlings. As is evident from a plot of the data (Figure 2), species and sex affected body mass, whence we controlled for these factors: There were 26 female and 31 male blue tits and 12 female and 12 male great tits. To keep sample sizes large enough for statistically significant conclusions we did not consider further stratifications.

To study the association between environmental variables and growth we used the following information about the nest-boxes that the above-cited authors had retrieved from geo-information systems or collected at the eight visits. The latter data measured human activity, air temperature at the nest, and light and sound pollution, the former were distance to paths and roads, percentage of impervious surface (ISA), tree cover and NDVI (normalized difference vegetation index). Table 1 informs about the upper and lower observed values (and it adds further explanations about the indices). Methodology details can be found in Corsini et al. (2017), Corsini et al. (2019) and Szulkin et al. (2020).

Data fitting

Using the growth data, we aimed at finding parameters that minimized *SSLE*, the sum of squared errors for fitting the logarithmically transformed growth function to logarithmically transformed mass-data. If $m(t)$ is a solution of equation (1), using certain exponents $a < b$ and parameters p, q, c, and if (t_i, m_i) are the mass-at-age data (for our data: $n = 8$), then *SSLE* is defined by equation (3):

$$(3) \quad SSLE = \sum_{i=1}^{n} \left(\ln(m_i) - \ln\left(m(t_i)\right)\right)^2$$

We used *SSLE* instead of the more common sum of squared errors for model comparison because this measure was less sensitive to higher variances for the higher masses. For, shortly before fledging the body mass of birds may reach a peak and then go down or begin to fluctuate (Brown et al., 2007). The suitability of this approach has already been confirmed for chicken (Renner-Martin et al., 2019) and other dinosaurs (Brunner et al., 2019). As was explained there, the implicit statistical assumption for *SSLE* is a lognormal distribution of mass. (For this distribution, variance increases with mass.)

For the search of optimal parameters that minimized *SSLE*, standard optimization tools in general failed, even if they started close to the best-fit parameters (Renner-Martin et al., 2018; for related observations: Loibel et al., 2010; Shi et al., 2014). We therefore used a grid-search combined with a custom-made variant of the method of simulated annealing (Vidal, 1993). The details of our approach to optimization were outlined elsewhere (Brunner et al., 2019, Renner-Martin et al., 2019). We did not use simplifications that are common in growth literature. For example, for the differential equation (1), the first weighing at day 1 (hatching) corresponded to the age $t = 0$. Many authors defined the parameter c by the hatching mass $m(0) = c$, as then only the four remaining parameters needed to be optimized. In contrast, we used hatching mass as an estimate for the initial value but allowed the optimization to identify a better value of c to find a lower *SSLE*.

Computations and statistics

We used Mathematica 12.1 of Wolfram Research for computer algebra and statistical computations. Thereby, for data-fitting we used the custom-made optimization method described above. The Mathematica-code was explained elsewhere (Brunner et al., 2019, Renner-Martin et al., 2019) and it is available as a supporting information for the cited papers.

For all statistical tests we decided at 95% significance (P-value 5%). Thereby, we preferred non-parametric methods suitable for small samples (Hollander & Wolfe 1999). For correlations we computed the Spearman rho and used the Spearman rank test for assessing its significance. We used the one-sided sign test to estimate confidence intervals for the median. When we compared the medians of different samples, we used the Mann-Whitney test.

Anderson-Darling test was used to test distribution assumptions, specifically the above assumption of lognormally distributed mass.

Results

Best-fit model parameters

We first tested the assumption of log-normal distributions of the masses. This assumption is of interest, because under this assumption the *SSLE*-parameters would be maximum likelihood estimates and therefore asymptotically normally distributed. Hence, statistics derived from *SSLE* (e.g. R-squared, F-ratio test or the Akaike information criterion) could be used to compare models and decide, if simpler models, such as logistic growth, would be preferable over the more general BP-model. For our data, the Anderson-Darling test did refute this assumption for only six (7.4% of 81) birds. Nevertheless, we did not discuss model

selection, as we had decided to apply the BP-model, whereby we perceived the best-fit model curve as a smoothening of the growth data.

Table 2 gives an overview of the range of the best-fit model parameters and several statistics computed from them. (Details for each individual bird are provided as a supporting information.) In view of an excessively large asymptotic size for one bird, we first analyzed the quotient of the asymptotic mass over the maximal observed mass (in general, mass at day 15): It ranged from 0.89 to $1.4 \cdot 10^{13}$. However, the median was 1.006 with a narrow confidence interval between 0.998 and 1.016 (one-sided sign tests: P-values 2.24%, i.e. 95.5% confidence).

The focus of this research was on the ratio m_{infl}/m_{max} of the mass at the inflection-point (peak growth) over the asymptotic mass. For five birds there was no inflection point, $a = 0$; for them we set $m_{infl} = 0$ and $t_{infl} = 0$. Mathematically, a higher/lower ratio was related to the shape of the BP-growth curves. This could be visualized by a specific example, the growth trajectories and the inflection points of logistic growth with ratio 0.5 and of an alternative growth curve with a higher ratio 0.63 (Figure 3). Both curves were defined to start at the same initial value ($c = 0.1$), approach the same asymptotic limit 1, and intersect at the inflection point of the logistic growth curve. Consequently, for the alternative curve the parameters p and q were smaller than the same parameters for logistic growth. Further, the alternative curve had a higher inflection point that was reached later (higher t_{infl}) and that was sooner close to the asymptotic limit. This difference in the growth patterns may be interpreted as a faster/slower growth for a higher/lower ratio m_{infl}/m_{max}. (Note that in this example the final sizes for both growth curves were the same. However, there might be a biological advantage if the final size is approached earlier and the ratio explores this matter.)

The following associations demonstrate that a similar pattern was observed for the bird-data: Using the full sample of 81 birds, with high significance a higher ratio m_{infl}/m_{max} was associated a) to a higher mass m_{infl} (Spearman rho 0.47; P-value close to zero for the Spearman rank test), b) to a lower asymptotic mass m_{max} (Spearman rho –0.35, P-value 0.1%), c) to a longer time span t_{infl} till maximal growth (Spearman rho 0.78, P-value close to zero), and d) to lower parameter values p and q (Spearman rho –0.57 and –0.52, respectively, with P-values close to zero). To study this pattern, we defined two classes of birds from their ratio m_{infl}/m_{max}: Using the ratio of logistic growth as a threshold, birds with ratios $m_{infl}/m_{max} > 0.5$ were high-ratio birds (54% of our sample) and the other low-ratio birds.

Further, for our data a higher ratio was associated to a higher exponent-difference $b–a$ (Spearman rho 0.5 with P-value close to zero). This was surprising, as a negative correlation would be expected: In equation (2), if the smaller exponent (a) was kept fixed while the larger one (b) increased, then the ratio decreased. This indicated a peculiarity of the distribution of best-fit exponent-pairs (plot: Figure 4). The exponent-pairs appeared to be scattered at random, but a notable observation was the concentration close to the diagonal: Defining a bird as near-diagonal, if its best-fit exponent pair satisfies $b–a < 1.5$ (yellow lines in Figure 4), then 47% of the birds were near-diagonal (the other birds are far-diagonal).

Associations between environmental and model parameters

For this section we systematically searched for significant associations between the indicators of Table 1 and two model parameters for the shape of the growth curves, the ratio m_{infl}/m_{max} and the exponent-difference $b–a$. The stratification of the 81 birds by species and gender

defined four samples (BT-F, BT-M, GT-F, and GT-M). In the sequel this resulted in small to moderate sample sizes: 12-31 individuals for the samples used for Table 3 and at least three individuals in each of the subsamples for Table 4 and Table 5. We searched under three different perspectives.

First, we identified the significant correlations between the indicators and the two shape parameters). With respect to the ratio (left half of Table 3), most of the nine significant correlations were highly significant (P-value below 1%, using the Spearman rank test) and strong (Spearman rho with absolute value 0.5 or higher). As a common observation for all groups, except male blue tits, a larger initial count of hatchlings was associated to a lower ratio m_{infl}/m_{max} (three correlations). Further, both for female and for male blue tits, a higher percentage of impervious area around the nest-box and a higher level of light pollution was associated to a lower ratio m_{infl}/m_{max} (four correlations). There were eight significant correlations between environmental indicators and the exponent-difference $b–a$ (right half of Table 3), in particular the following: For female and male blue tits, a higher level of light pollution was associated to a lower exponent-difference, and for female and male great tits a higher distance to a path was related to a lower exponent-difference. The maximal number of significant correlations in Table 3 was observed for the sample GT-F with respect to the ratio (4, compared to 1-2 for the other samples).

Second, we complemented the study of individual correlations by a comparison at the population level. We compared the overall environmental situation between two complementary groups of birds that were defined from the shape parameters (high-ratio vs. other birds, and near-diagonal vs. other birds, respectively). Thus, for each indicator we computed its median for each of the two complementary groups of birds, and we tested, if the two medians differed significantly (Mann-Whitney test), resulting in 24 significant outcomes. Thereby, several of the 17 significant correlations of Table 3 could be explained by related differences in the environments of the nest-sites that were chosen by (the parents of) these birds (Table 4): The negative correlation between nest size and the ratio m_{infl}/m_{max} was explained by the observation that for all samples (except BT-M) in the median the high-ratio birds had fewer siblings than the other birds. For blue tits, the negative correlation of the ratio with the percentage of impervious area was confirmed by the lower median of this percentage for high-ratio birds. Further, Table 4 identified significant differences in the environmental conditions, specifically for the comparison of near-diagonal and other birds, that were not detected from the correlations. The maximal number of significant associations in Table 4 was observed for the sample GT-F with respect to the exponent-difference (8, compared to 0-4 for the other samples).

Third, we tested the hypothesis that the shape of the growth curves might have been affected by favorable (or unfavorable) environments. To this end, for each indicator we divided our samples (i.e. BT-F, BT-M, GT-F, GT-M) into two groups of approximately equal sizes, namely birds that grew up in an ambience with a high parameter value (larger than the sample median) and the other birds. We then computed the medians of the shape parameters and used the Mann-Whitney test to check for stochastic differences. This resulted in 20 significant differences (Table 5). For example, for the two GT-samples the ratio m_{infl}/m_{max} was smaller for birds that had more siblings than the median than for the other ones. (The same was observed for BT-F However, its division was unbalanced.) Further, as with the negative Spearman rho of Table 3, a higher percentage of impervious area and higher light pollution was associated to a significantly smaller median ratio for the blue tits from such

neighborhoods. For light pollution, this observation was confirmed by the exponent-difference (which is correlated to the ratio). The maximal number of significant associations in Table 5 was observed for the sample GT-M with respect to the exponent-difference (6, compared to 1-4 for the other samples).

Discussion and conclusions

We studied indicators from avian literature that potentially affect the growth of birds. However, previously such findings were based on comparisons of large samples from clearly distinct environments, e.g. the urban and rural dichotomy. In our context, the sample size was comparably small, and we expected that the environmental variation across the urban study site (Pole Mokotowskie park) would be gradual. Could we nevertheless identify significantly different growth patterns?

For instance, it is plausible that the efforts of parents to feed their nestlings may not grow proportional to the number of nestlings, whence more siblings to feed may reduce the efforts per nestling and affect the shape of the nestlings' growth curves. Considering the small sample sizes, were the changes distinct enough so that statistical tests could detect them? Thus, it was not clear a priori, whether these changes would occur for all nests in about the same way. Further, why should the two considered shape parameters (ratio m_{infl}/m_{max} and exponent-difference $b–a$) be sensitive to such changes? As was noted for Figure 4, a higher/lower ratio could be interpreted as faster/slower growth and the exponent-difference was correlated to the ratio, but there are many other shape parameters conceivable that could as well measure faster/slower growth. For another example, it is plausible that impervious area around the nest may inhibit the success of fetching food and affect the growth of the nestlings. However, why should this matter within the narrow confines of an urban park?

To overcome these difficulties, we increased the number of tests. In total, for each of the 11 environmental indicators we have applied six tests to each sample, resulting in 264 tests for significance (the entries in Tables 3 to 5). Amongst them, at most 19 outcomes would be spurious, recording a false significance (P-value 4.4% for a binomial distribution assuming a 5% chance for a false "significant"), while for our samples 61 outcomes were significant. Further, several of these outcomes were highly significant, or they were significant under two or three aspects (i.e. they occurred in 2-3 tables), or they were observed for two or more samples. For such outcomes there may have been indeed an association between the environmental indicator and the considered shape parameter. Thereby, we obtained the maximal number of significant associations for light pollution (11 of 24 tests were significant), followed by the impervious area (10 significant tests) and the count of hatchlings (9 significant tests); for the other indicators, between seven and none (temperature) of the tests were significant.

We conclude that despite the small sample size the BP-model could detect significant statistical association between some local environmental variables and certain shape parameters of the BP-model, namely the ratio m_{infl}/m_{max} and the exponent-difference $b–a$. Thereby the exponent-difference was meaningful only for the BP-model, while the ratio would be meaningful for all other models. However, as noted in the introduction, for many common three-parameter models of avian literature the ratio is fixed (e.g. ratio 0.5 for logistic growth). We conclude that these more traditional three-parameter models are not suitable to explore and compare the shapes of growth curves. We recommend the BP-model for such a purpose.

Acknowledgements and declarations

This paper originated from conversations with Michela Corsini, Eva Maria Schöll and Irene Di Lecce, who provided and explained to us the raw data for their paper Corsini et al. (2020). The authors are grateful to Michaela Corsini and Júlio Neto, who provided insightful comments on preliminary versions of our paper. Further, the authors appreciate the helpful remarks by the editors and reviewers of this journal.

The authors declare no competing interests and there occurred no ethical issues, as the paper was based on literature data from ethically approved research.

This source also links to the repository for these data. The Mathematica code for optimization is stored in repositories for Brunner et al. (2019) and Renner-Martin et al. (2018, 2019). In addition, the authors have prepared supporting information. [Links, file names and descriptions will be added upon publication.]

References

Aggrey S.E. (2002) Comparison of three nonlinear and spline regression models for describing chicken growth curves. Poultry Science 81:1782-1788.

Bańbura J., Bańbura M. (2012) Blue tits Cyanistes caeruleus and great tits Parus major as urban habitat breeders. Inter Studies Sparrows 36: 66-72.

BertalanffyL.v. (1949) Problems of organic growth. Nature 163: 156-158.

BertalanffyL.v. (1957) Quantitative laws in metabolism and growth. Quarterly Revues in Biology 32: 217-231.

Bleu J, Agostini S, Biard C (2017) Nest-box temperature affects clutch size, incubation initiation, and nestling health in great tits. Behavioral Ecology 28: 793-802.

Brody S. (1945) Bioenergetics and Growth. Hafner Publ. Comp., New York, NY, USA.

Brown W.P., Eggermont P., LaRiccia V., Roth R.R. (2007) Are parametric models suitable for estimating avian growth rates? Journal of Avian Biology 38: 495-506.

Brunner N., Kühleitner M., Nowak W.G., Renner-Martin K., Scheicher K. (2019) Comparing growth patterns of three species: Similarities and differences. PLoS ONE 14, published online: DOI 10.1371/journal.pone.0224168.

Calder WA III (1985) Size, Function, and Life History. Harvard Univ. Press, Cambridge, MA, USA.

Corsini M., Dubiec A., Marrot P., Szulkin M. (2017) Humans and Tits in the City: Quantifying the Effects of Human Presence on Great Tit and Blue Tit Reproductive Trait inVariation. Frontiers in Ecology and Evolution 5, published online: DOI 10.3389/fevo.2017.00082.

Corsini M., Marrot P., Szulkin M. (2019) Quantifying human presence in a heterogeneous urban landscape. Behavioral Ecology30: 1632-1641.

Corsini M., Schöll E.M., Di Lecce I., Chatelain, M., Dubiec A., Szulkin M. (2020) Growing in the city: urban evolutionary ecology of avian growth rates. Evolutionary Applications, published online: DOI 10.1111/eva.13081.

Da Silva A., Samplonius J.M., Schlicht E., Valcu M., Kempenaers B. (2014) Artificial night lighting rather than traffic noise affects the daily timing of dawn and dusk singing in common European songbirds. Behavioural Ecology 25: 1037-1047.

Francis C.D. (2015) Vocal traits and diet explain avian sensitivities to anthropogenic noise. Global Change Biology 21: 1809-1820.

Garcia O. (1983) A stochastic differential equation model for the height growth of forest stands. Biometrics 39: 1059-1072.

Gladalski M., Banbura M., Kalinski A., Markowski M., Skwarska J., Wawrzyniak J., Zielinski P., Cyzewska I., Mankowska D., Banbura J.(2016) Effects of human-related disturbance on breedingsuccess of urban and non-urban blue tits (Cyanistes caeruleus). Urban Ecosystems 19, published online: DOI 10.1007/s11252-016-0543-3.

Gompertz B. (1832) On the nature of the function expressive of the law of human mortality, and on a new mode of determining the value of life contingencies. Philos. Trans. R. Soc. London 123: 513-585.

Grossman M. (1988) Multiphasic analysis of growth curves in chicken. Poultry Science 67: 33-42.

Halfwerk W., Holleman L.J., Lessells C.K., Slabbekoorn H. (2011) Negative impact of traffic noise on avian reproductive success. Journal of Applied Ecology 48: 210-219.

Hollander M.A., Wolfe D.E. (1999) Nonparametric Statistical Methods, Wiley, Chichester, UK.

Hsing Y. (2005) Economic growth and income inequality: the case of the US. International Journal of Social Economics 32: 639-647.

Jacobs J., Rockne R.C., Hawkins-Daarud A.J., Jackson P.R., Johnston S.K., Kinahan P., Swanson K.R. (2019) Improved model prediction of glioma growthutilizing tissue-specific boundary effects. Mathematical Biosciences 312: 59-66.

Katsanevakis S., Maravelias C.D. (2008) Modelling fish growth: multi-model inferenceas a better alternative to a priori using von Bertalanffy equation. Fish and Fisheries9:178-187.

Kirschel A.N., Blumstein D.T., Cohen R.E., Buermann W., Smith T.B., Slabbekoorn H. (2009) Birdsong tuned to the environment: green hylia song varies with elevation, tree cover, and noise. Behavioral Ecology 20: 1089-1095.

Kuhi H.D., Porter T., López S., Kebreab E., Strathe A., Dumas A., France J. (2010) A review of mathematical functions for the analysis of growth in poultry. Worlds Poultry Science Journal 66: 227-240.

Loibel, S., Andrade M.G., do Val J.B.R., de Freitas A.R. (2010) Richards growth model and viability indicators for populations subject to interventions. Anais da Academia Brasileira de Ciências 82, published online: DOI 10.1590/S0001-37652010000400028.

Maceina M.J. (2007) Use of piecewise nonlinear models to estimate variable size-relatedmortality rates. North American Journal of Fisheries Management 27:971-977.

Manabe A., Yamakawa T., Ohnishi S., Akamine T., Narimatsu Y., Tanaka H., Funamoto T., Ueda Y., Yamamoto T. (2018) A novel growth function incorporating the effects of reproductive energy allocation. PLoS ONE 13, published online: DOI 10.1371/journal.pone.0199346

Marusic M., Bajzer Z. (1993) Generalized Two-Parameter Equations of Growth. Journal of Mathematical Analysis and Applications 179: 446-462.

Naef-Daenzer B., Keller L.K. (1999) The foraging performance of great and blue tits (Parus major and P. caeruleus) in relation to Caterpillar development, and its consequences for nestling growth and fledging weight. Journal of Animal Ecology 68: 708-718.

Ndithia H., Bakari S., Matson K., Muchai M., Tieleman I. (2017) Geographical and temporal variation in environmental conditions affects nestling growth but not immune function in a year-round breeding equatorial lark. Frontiers in Zoology 14, published online: DOI 10.1186/s12983-017-0213-1.

Ohnishi S., Yamakawa T., Akamine T. (2014) On the Analytical Solution for the Pütter-Bertalanffy Growth Equation. Journal of Theoretical Biology 343: 174-177.

Pauly D., Cheung W.W.L. (2018) Sound physiological knowledge and principles in modeling shrinking of fishes under climate change. Global Change Biology 24, published online: DOI 10.1111/gcb.13831.

Pinheiro J.C., Bates D.M. (2000) Mixed-Effects Models in S and S-Plus. Springer Publishing, New York, USA.

Pütter A. (1920) Studien über physiologische Ähnlichkeit. VI. Wachstumsähnlichkeiten. Pflügers Archiv für die Gesamte Physiologie des Menschen und der Tiere 180: 298-340.

Ricklefs R.E. (1967) A graphical method of fitting equations to growth curves. Ecology 48: 978-983.

Ricklefs R.E. (1984) The Optimization of Growth Rate in Altricial Birds. Ecology 65: 1602-1616.

Remacha C., Delgado J.A., Bulaic M., Pérez-Tris J. (2016) Human disturbance during early life impairs nestling growth in birds inhabiting a nature recreation area. PLoS ONE 11, published online: DOI 10.1371/journal.pone.0166748.

Remeš V. (2007) Avian growth and development rates and age‐ specific mortality: the roles of nest predation and adult mortality. Evolutionary Biology 20: 320-325.

Renner-Martin K., Brunner N., Kühleitner M., Nowak W.G., Scheicher K. (2018) On the Exponent in the Von Bertalanffy Growth Model. PeerJ 6, published online:pmid 29312827.

Renner-Martin K., Brunner N., Kühleitner M., Nowak W.G., Scheicher K. (2019) Best-fitting Growth Curves of the Von Bertalanffy-Pütter Type. Poultry Science 98: 3587-3592.

Shi P.J., Ishikawa T., Sandhu H.S., Hui C., Chakraborty A., Jin X.S., Tachihara K., Li B.L. (2014). On the 3/4-exponent van Bertalanffy equation for ontogenetic growth. Ecological Modelling 276:23-28.

Serroyen J., Molenberghs G., Verbek G., Davidian M. (2009) Non-linear Models for Longitudinal Data. American Statistician 63: 378-388.

Stewart J., Robbins W.D., Rowling K., Hegarty A., Gould A. (2013) A multifaceted approach to modelling growth of the Australian bonito, Sarda australis (Family Scombridae), with some observations on its reproductive biology. Marine and Freshwater Research 64: 671-678.

Strathe A.B., Danfær A., Sørensen H., Kebreab E. (2010) A multilevel nonlinear mixed effects approach to model growth in pigs. Journal of Animal Science 88:638-649.

Szulkin M., Garroway C.J., Corsini M., Kotarba A.Z., Dominoni D. (2020) How to quantify urbanization when testing for urban evolution? In: Szulkin M., Munshi-South J., Charmantier A. (Eds.), Urban Evolutionary Biology. Oxford University Press, in print.

Tjørve K.M.C., Tjørve E. (2010) Shapes and functions of bird-growth models: how to characterize chick postnatal growth. Zoology 113: 326-333.

Tjørve K.M.C., Tjørve E. (2017) Modelling avian growth with the Unified-Richards: As exemplified by wader-chick growth. Journal of Avian Biology 48: 770-784.

Verhulst P.F. (1838) Notice sur la loi que la population suit dans son accroissement. Curr. Math. Phys. 10:113-121.

Vidal R.V.V. (1993) Applied Simulated Annealing. In: Lecture notes in economics and mathematical systems. Berlin: Springer-Verlag.

Vrána J., Remeš V., Matysioková B., Tjørve K.M.C., Tjørve E. (2019) Choosing the right sigmoid growth function using the unified-models approach. Ibis 161: 13-26.

West G.B., Brown J.H., Enquist B.J. (2001) A general model for ontogenetic growth. Nature 413:628-631.

West G.B., Brown J.H., Enquist B.J. (2004) Growth models based on first principles or phenomenology? Functional Ecology 18: 188-196.

White C.R. (2010) Physiology: there is no single p. Nature 464: 691-693.

Tables

Table 1. Range of the environmental data for the nest-box neighborhoods.

Indicator	BT-F			BT-M			GT-F			GT-M		
	Minimum	Median	Maximum	Minimum	Median	Maximum	Minimum	Median	Maximum	Minimum	Median	Maximum
Hatching day[1]	31 d	38 d	43 d	36 d	38 d	60 d	33 d	36 d	45 d	33 d	38 d	45 d
Hatchlings[2]	5	9	12	5	7	12	4	7	8	4	7	8
Human activity[3]	0.1	1.1	2.1	0	1.05	2.1	0	1.1	3.35	0	0.75	3.35
ISA[4]	0.57%	4.59%	26.26%	0.57%	4.49%	26.26%	5.12%	10.38%	21.94%	5.12%	9.07%	21.94%
Light[5]	2399 lx	3160 lx	11,463 lx	2384 lx	2852 lx	11,463 lx	2539 lx	8588 lx	13,848 lx	2539 lx	4907 lx	13,848 lx
NDVI[6]	0.49	0.76	0.81	0.49	0.75	0.81	0.65	0.69	0.82	0.65	0.71	0.82
Path[7]	6 m	14.3 m	108.5 m	4.3 m	11.1 m	31.5 m	3.8 m	5.55 m	48.3 m	3.8 m	6.9 m	48.3 m
Road[8]	2 m	85.4 m	256.3 m	2 m	165.1 m	256.3 m	32 m	63.7 m	152.8 m	32 m	106.9 m	152.8 m
Sound[9]	62.06 db	65.44 db	74.63 db	62.06 db	65.93 db	74.63 db	62.46 db	65.41 db	74.85 db	62.46 db	66.98 db	74.85 db
Temperature[10]	10.37°C	10.86°C	12.03°C	10.37°C	10.86°C	14.16°C	10.62°C	11.04°C	12.96°C	10.62°C	11.08°C	12.96°C
Tree cover[11]	3.26%	28.13%	51.94%	2.29%	19.07%	51.94%	22.84%	30.46%	59.12%	12.47%	24.90%	59.12%

Notes: All numbers were rounded to the shown decimal. Environmental indicators in alphabetic order: [1] count of days from the start of the field work to hatching; [2] initial count of hatchlings in each nest; [3] index of human activity from Corsini et al. (2017); [4] percentage of impervious area around the nest-box; [5] light pollution at the nest-box site (in lx); [6] index for the vegetation cover around the nest-box; [7] minimal (Euclidean) distance to the next path (in m); [8] minimal (Euclidean) distance from the nest-box to (the center of) the next road (in m); [9] sound pollution at the nest-box site (in db); [10] average of the morning temperatures at the eight visits (in °C); [11] tree cover around the nest-box (percent).

Table 2. Range of the model parameters for the sample of 81 birds.

Parameter	BT-F			BT-M			GT-F			GT-M		
	Minimum	Median	Maximum	Minimum	Median	Maximum	Minimum	Median	Maximum	Minimum	Median	Maximum
Exponent *a*	0	0.495	1.61	0.05	0.63	1.52	0.01	0.815	1.24	0.04	0.835	1.6
Exponent *b*	0.01	2.28	23.53	0.44	2.31	18.25	1.33	3.155	7.42	1.17	3.69	11.28
Initial mass *c*	0.80 g	1.01 g	1.51 g	0.80 g	1.10 g	1.79 g	1.00 g	1.50 g	1.77 g	1.00 g	1.26 g	1.57 g
Parameter *p*	0.32	0.71	20.79	0.31	0.67	2.05	0.34	0.91	1.52	0.30	0.96	8.38
Parameter *q*	1.15E-22	0.01	20.01	1.59E-18	0.01	1.54	4.59E-09	0.002	1.04	1.56E-13	6.79E-05	8.14
SSLE	0.0018	0.0107	0.0481	0.0010	0.0087	0.1638	0.0049	0.0113	0.0221	0.0008	0.0111	0.0258
Difference b–a	0.01	1.8	23.21	0.01	1.67	18	0.14	2.51	7.41	0.01	3.29	10.82
Mass m_{max}	6.290 g	9.225 g	45.848 g	8.451 g	10.190 g	1.41E+114 g	13.39 g	15.00 g	18.26 g	13.93 g	15.63 g	19.29 g
Time t_{infl}	0 d	4.804 d	6.867 d	0	5.075 d	7.32 d	3.35 d	5.03 d	7.46 d	3.59 d	5.38 d	8.67 d
Mass m_{infl}	0 g	4.809 g	7.169 g	0	5.337 g	7.17 g	5.48 g	7.20 g	9.68 g	6.63 g	8.29 g	10.73 g
Ratio m_{infl}/m_{max}	0	0.51	0.83	0	0.50	0.79	0.41	0.46	0.67	0.39	0.53	0.74

Note: All numbers rounded to the shown decimals. Parameter *q* and mass m_{max}: x.xxEy means x.xx times 10^y. Time t_{infl}: Day 1 of the data means time $t = 0$ of the model; $t_{infl} = 0$ means no inflection point. Mass m_{infl}: 0 means no inflection point.

Table 3. Spearman rho and significance.

Indicator	Comparing indicator with inflection-point-mass over asymptotic mass				Comparing indicator with the exponent-difference			
	BT-F (26)	BT-M (31)	GT-F (12)	GT-M (12)	BT-F (26)	BT-M (31)	GT-F (12)	GT-M (12)
Hatching day	not significant	not significant	not significant	not significant	0.48 (1.394%)	not significant	–0.59 (4.236%)	not significant
Hatchlings	–0.59 (0.152%)	not significant	–0.86 (0.033%)	–0.78 (0.269%)	not significant	not significant	not significant	not significant
Human activity	not significant	not significant	not significant	not significant	not significant	not significant	not significant	not significant
ISA	–0.61 (0.091%)	–0.49 (0.537%)	not significant	not significant	not significant	not significant	0.71 (0.899%)	not significant
Light	–0.60 (0.133%)	–0.55 (0.134%)	not significant	not significant	–0.61 (0.0935%)	–0.43 (1.514%)	not significant	not significant
NDVI	not significant	not significant	not significant	0.70 (1.106%)	not significant	not significant	not significant	not significant
Path	not significant	not significant	not significant	not significant	not significant	0.42 (2.024%)	–0.82 (0.123%)	–0.65 (2.292%)
Road	not significant	not significant	not significant	not significant	not significant	not significant	not significant	not significant
Sound	–0.48 (1.321%)	not significant	not significant	not significant	not significant	not significant	not significant	not significant
Temperature	not significant	not significant	not significant	not significant	not significant	not significant	not significant	not significant
Tree cover	not significant	not significant	not significant	not significant	not significant	not significant	not significant	not significant

Note: Indicators are explained in Table 1. Entries “x (y%)” inform about the value x of Spearmann rho and the P-value y for the Spearman rank test, whereby the second row informs about the considered sample and the first column informs about the indicator that was compared with m_{infl}/m_{max} and $b–a$, respectively; “not significant” indicates a P-value y > 5%.

Table 4. Comparing the medians of the environmental indicators for different groups of birds.

Indicator	High-ratio (H) compared to low-ratio (L) birds				Near-diagonal (N) compared to far-diagonal (F) birds			
	BT-F (15 H, 11 L)	BT-M (19 H, 12 L)	GT-F (3 H, 9 L)	GT-M (7 H, 5 L)	BT-F (13 N, 13 F)	BT-M (15 N, 16 F)	GT-F (5 N, 7 F)	GT-M (5 N, 7 F)
Hatching day	not significant	not significant	not significant	not significant	36 d< 38 d (0.199%)	Medians 38 d; mean 40.2 d > 40.19 d (4.5%)	36 > 33 (4.794%)	not significant
Hatchlings	9 < 11 (0.346%)	not significant	4 < 8 (2.35%)	4 < 8 (2.536%)	not significant	not significant	not significant	not significant
Human activity	not significant	not significant	not significant	not significant	not significant	1.05 < 1.8 (1.607%)	0 < 1.1 (4.528%)	not significant
ISA	4.5% < 11.7% (0.663%)	3.6% < 8.9% (1.137%)	not significant	not significant	not significant	5.5% > 3.6% (3.671%)	5.1% < 11.7% (0.457%)	not significant
Light	not significant	2852 lx < 3160 lx (2.0%)	not significant	not significant	4420 lx > 2826 lx (1.426%)	not significant	4907 lx < 9988 lx (4.528%)	not significant
NDVI	not significant	not significant	not significant	0.78 > 0.69 (4.728%)	not significant	not significant	not significant	not significant
Path	not significant	not significant	not significant	not significant	not significant	6 m < 19 m (0.067%)	48.3 m > 3.8 m (0.267%)	not significant
Road	not significant	not significant	not significant	not significant	not significant	not significant	106.9 m > 44.2 m (1.951%)	not significant
Sound	64.0 db < 68.3 db (0.565%)	not significant	74.9 db > 65.4 db (0.783%)	not significant	not significant	not significant	62.5 db < 65.4 db (3.01%)	not significant
Temperature	not significant	not significant	not significant	not significant	not significant	not significant	not significant	not significant
Tree cover	not significant	not significant	22.8% < 30.5% (1.354%)	not significant	not significant	not significant	59.1% > 30.5% (1.951%)	not significant

Note: x<y (z%) informs about the median of the indicator values x for H (N), y for L (F) and about the P-value of the Mann-Whitney test to for a significant difference between the medians.

Table 5. Comparing the medians of ratios and exponent-differences of two groups of birds defined by ambient conditions above or below the median indicator value.

Indicator	BT-F			BT-M		
	counts	Medians: ratio	Medians: exp. diff.	counts	Medians: ratio	Medians: exp. diff.
Hatching day	H: 7, L: 19	not significant	not significant	H: 7, L: 24	not significant	not significant
Hatchlings	H: 9, L: 17	unbalanced	not significant	H: 14, L: 17	not significant	not significant
Human activity	H: 12, L: 14	not significant	not significant	H: 15, L: 16	not significant	3 > 0.22 (2.815%)
ISA	H: 13, L: 13	0.467 < 0.529 (0.868%)	not significant	H: 15, L: 16	0.45 < 0.561 (0.962%)	not significant
Light	H: 12, L: 14	0.459 < 0.544 (1.231%)	0.155 < 5.48 (0.184%)	H: 15, L: 16	0.483 < 0.529 (2.303%)	not significant
NDVI	H: 12, L: 14	not significant	not significant	H: 15, L: 16	not significant	not significant
Path	H: 11, L: 15	not significant	not significant	H: 15, L: 16	not significant	not significant
Road	H: 12, L: 14	not significant	4.835 > 0.185 (3.705%)	H: 13, L: 18	not significant	not significant
Sound	H: 13, L: 13	0.467 < 0.529 (3.068%)	not significant	H: 12, L: 19	unbalanced	not significant
Temperature	H: 12, L: 14	not significant	not significant	H: 12, L: 19	not significant	not significant
Tree cover	H: 10, L: 16	not significant	not significant	H: 13, L: 18	not significant	not significant
**Indicator**	**GT-F**			**GT-M**		
	counts	Medians: ratio	Medians: exp. diff.	counts	Medians: ratio	Medians: exp. diff.
Hatching day	H: 3, L: 9	not significant	not significant	H: 6, L: 6	not significant	not significant
Hatchlings	H: 5, L: 7	0.433 < 0.481 (0.577%)	not significant	H: 5, L: 7	0.45 < 0.544 (2.299%)	not significant
Human activity	H: 3, L: 9	not significant	not significant	H: 4, L: 8	not significant	not significant
ISA	H: 6, L: 6	not significant	5.025 > 0.245 (1.307%)	H: 5, L: 7	not significant	not significant
Light	H: 6, L: 6	not significant	5.025 > 0.245 (1.307%)	H: 5, L: 7	not significant	not significant
NDVI	H: 5, L: 7	not significant	0.23 < 4.56 (2,299%)	H: 6, L: 6	0.592 > 0.454 (4,533%)	not significant
Path	H: 6, L: 6	not significant	0.245 < 5.025 (0.824%)	H: 5, L: 7	not significant	0.14 < 4.5 (1.168%)
Road	H: 6, L: 6	not significant	0.245 < 5.025 (0.824%)	H: 5, L: 7	not significant	not significant
Sound	H: 4, L: 8	not significant	not significant	H: 5, L: 7	not significant	not significant
Temperature	H: 3, L: 9	not significant	not significant	H: 6, L: 6	not significant	not significant
Tree cover	H: 4, L: 8	not significant	0.21 < 4.355 (0.847%)	H: 5, L: 7	not significant	not significant

Note: counts "H: x, L: y" are the number x of birds, with H (the nest size had an indicator value larger than the median), and the number y of birds with L (the indicator was lower than or equal to the median), whereby the median of Table 1 was used (different medians of each indicator for species and sex); unbalanced indicates a significant difference, but the sample sizes for the above and below median birds differed by more than 2; x < y (z) informs about the medians of the ratios m_{infl}/m_{max} and the exponent-difference b–a, respectively, of the H and L groups, respectively, and z is the P-value of the Mann-Whitney test.

Figure captions

Figure 1: Weather data during May and June of 2016, showing minimal and maximal daily temperatures (°C) in blue and red, using the left axis, maximal daily precipitation rate (mm/h) in grey, using the right axis; source: open data from EPWA weather station (Warsaw Chopin airport), retrieved using Mathematica software.

Figure 2: Mass at days 1, 3, ..., and 15 plotted side-by-side; from left BT-F (red), BT-M (blue), GT-F (orange), and GT-M (cyan).

Figure 3: Growth trajectories of logistic growth and an alternative growth model and the inflection points. The growth curves had the parameters $a = 1$, $b = 2$, $c = 0.1$, $p = q = 1$ (blue: logistic growth) and $a = 1$, $b = 4$, $c = 0.1$, $p = q = 0.7526$ (red: alternative growth). The asymptotic limit was 1 (both curves) and the inflection point coordinates (gray dots) were (2.197, 0.5) for logistic growth and (2.572, 0.63) for the alternative curve.

Figure 4: Best-fit exponent-pairs for 81 nestlings (color code as in Figure 2) and yellow lines ($b = a$ and $b = a+1.5$) to indicate near-diagonal exponent-pairs.

Figure1

Click here to access/download;Figure;Figure1_Weather.tif

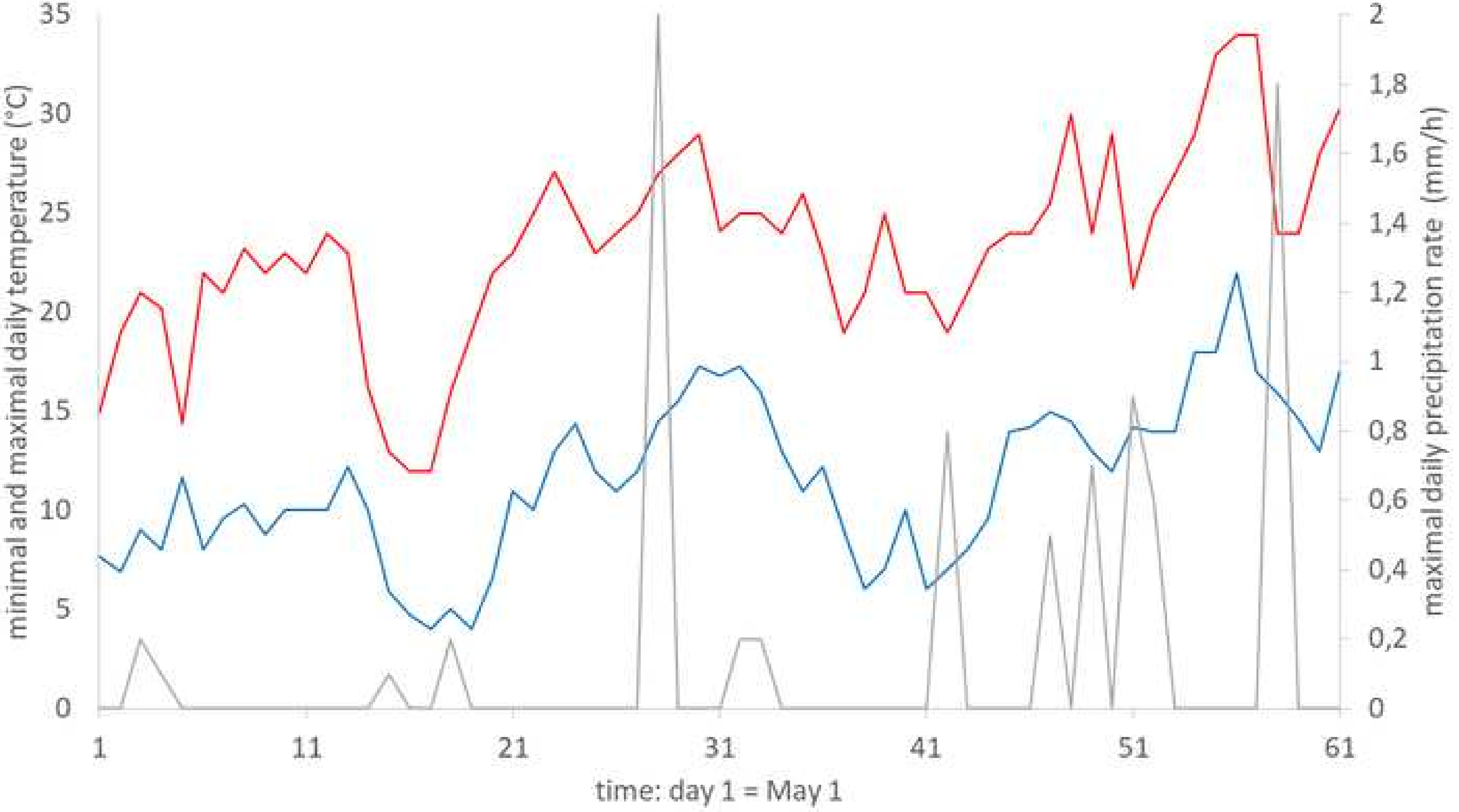

Figure2

Click here to access/download;Figure;Figure2_data.tif

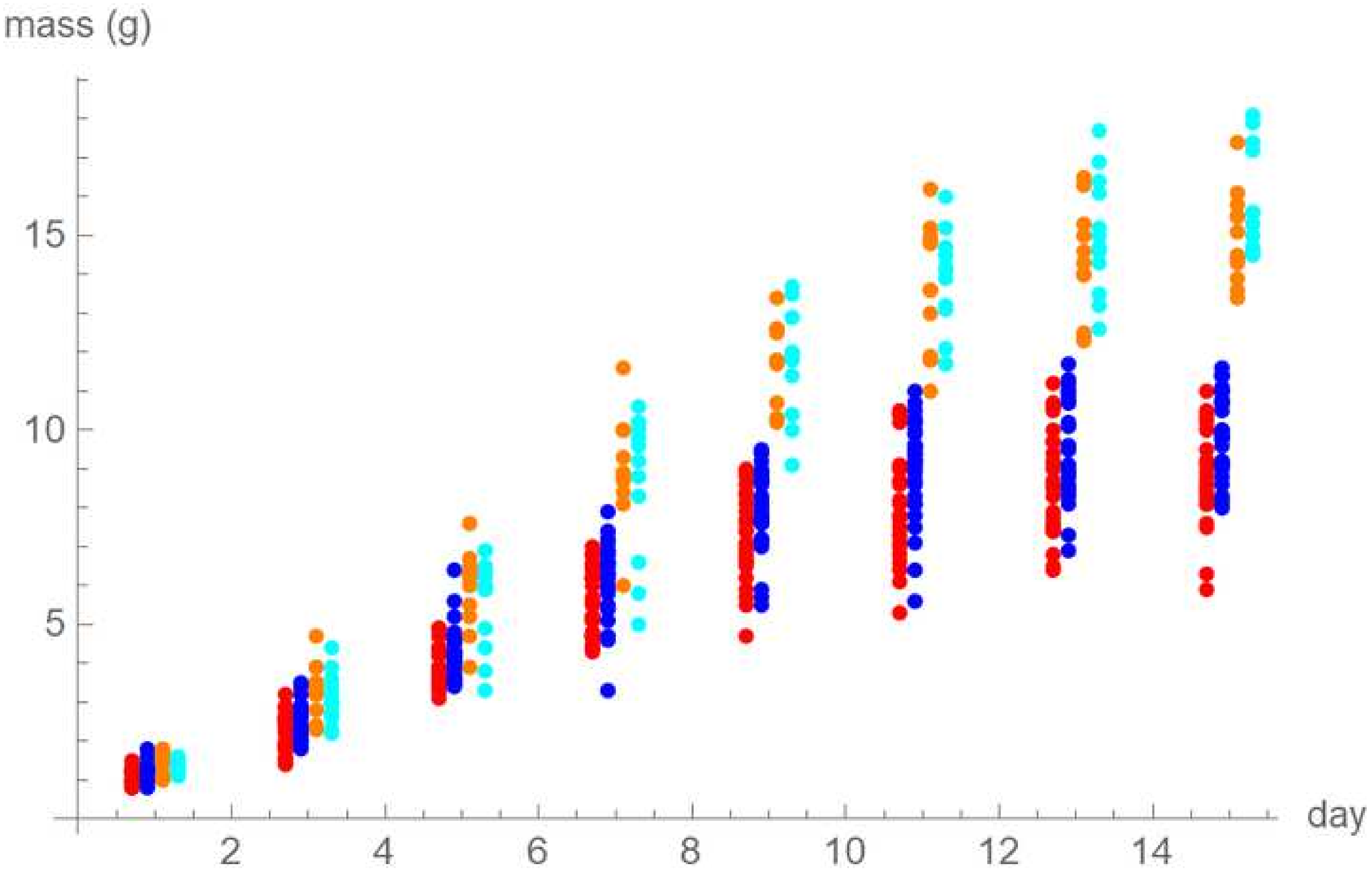

Figure3

Click here to access/download;Figure;Figure3_comparison.tif

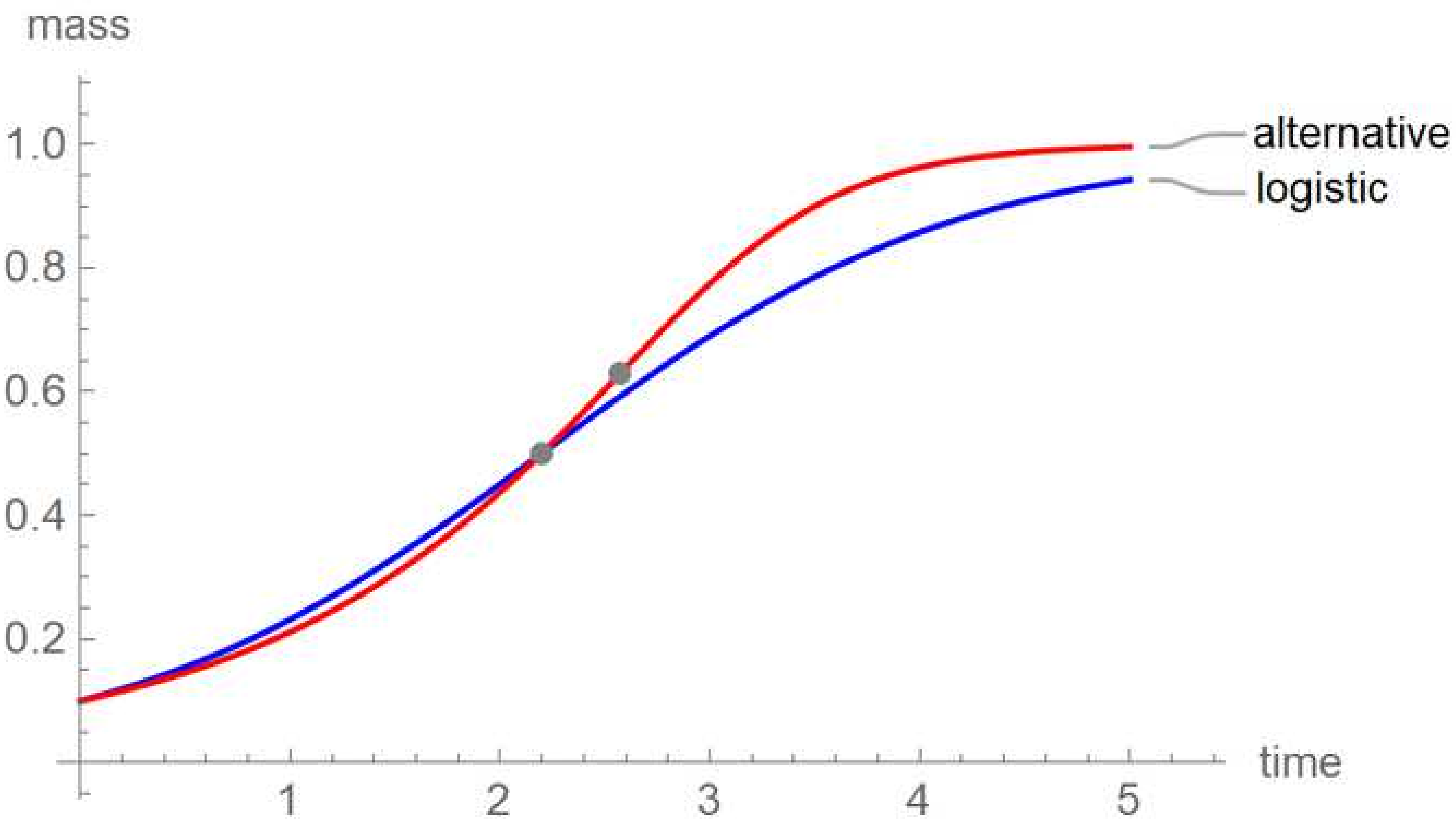

Click here to access/download;Figure;Figure4_Exponents.tif

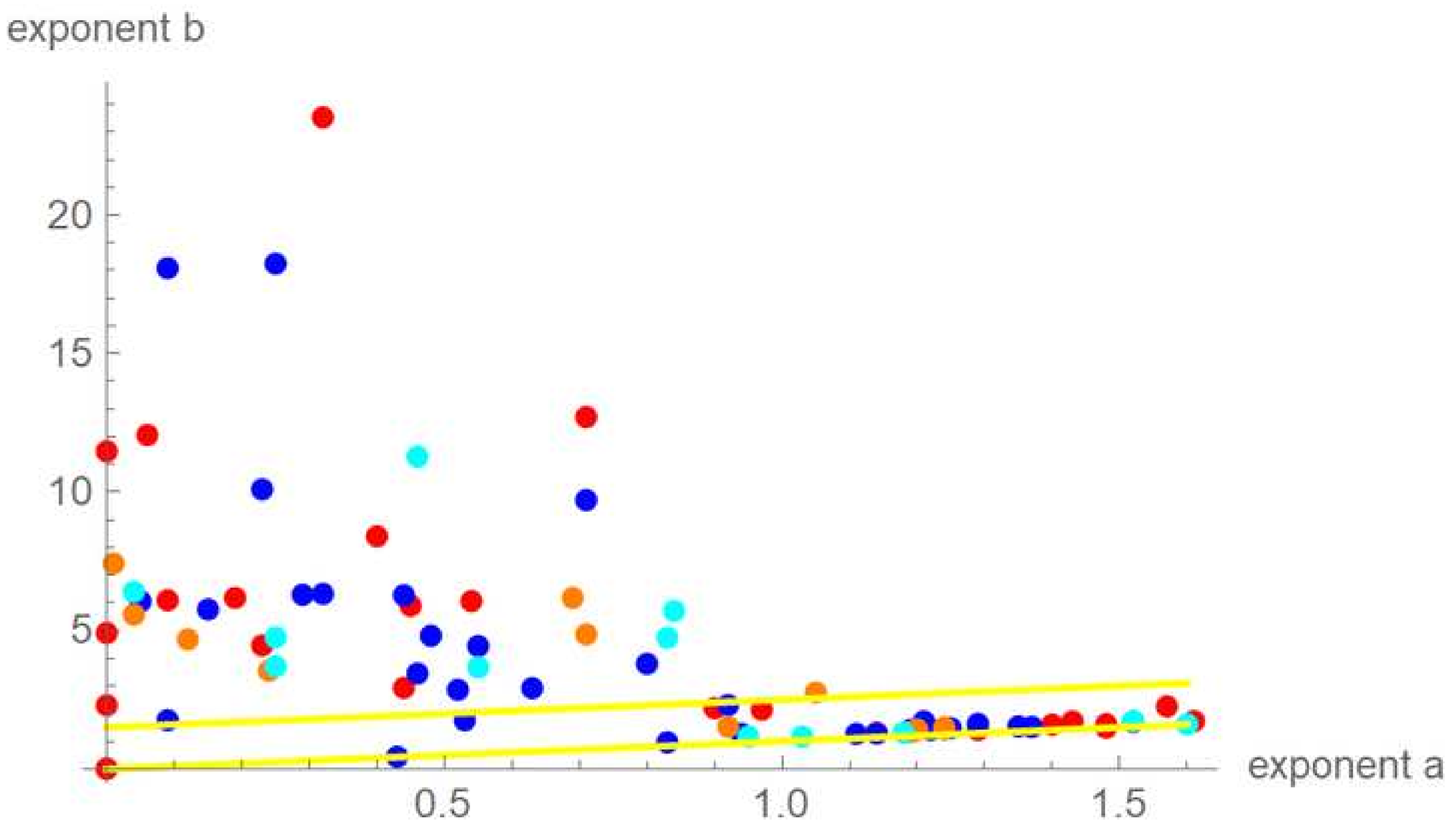

Data

Click here to access/download
Supporting Information - Compressed/ZIP File Archive
Data_for_internal_use_only.xlsx
