# Supplementary material for: Bertalanffy-Pütter models for avian growth
Source: PLoS One. 2021 Apr 26;16(4):e0250515. doi: 10.1371/journal.pone.0250515 (PMC8075225; doi:10.1371/journal.pone.0250515)
Supplement: Attachment — Submitted filename: PONE-D-20-31677_R1_reviewerSAO.pdf [file pone.0250515.s005.pdf]

PLOS ONE

Bertalanffy-Pütter models for avian growth

--Manuscript Draft--

**Manuscript Number:**	PONE-D-20-31677R1
**Article Type:**	Research Article
**Full Title:**	Bertalanffy-Pütter models for avian growth
**Short Title:**	Models for avian growth
**Corresponding Author:**	Manfred Kühleitner<br>University of natural resources and life sciences<br>Vienna, AUSTRIA
**Keywords:**	Cyanistes caeruleus; Parus Major; Bertalanffy-Pütter model; sum of squared logarithmically transformed errors
**Abstract:**	This paper explores the ratio of the mass in the inflection point over asymptotic mass for 81 nestlings of blue tits and great tits from an urban parkland in Warsaw, Poland (growth data from literature). The observed ratios ranged from 0 (non-sigmoidal growth for six birds) to 0.835. We computed the ratios using the Bertalanffy-Pütter model, because for other models there were a-priori restrictions on their possible range: For 19 birds the ratio was between 0.1 and 0.368 (lowest ratio attainable for the Richards model) and for 48 birds the ratio was above 0.5 (fixed ratio of logistic growth). With these ratios we were able to detect small variations in avian growth due to slight differences in the environment: For blue tits, a higher light pollution and a higher percentage of impervious area was associated to a lower ratio, which meant a slower growth.
**Order of Authors:**	Manfred Kühleitner
	Norbert Brunner
	Katharina Renner-Martin
**Response to Reviewers:**	See the attached File:<br><br>Response_to_Reviews_4
**Additional Information:**	
**Question**	**Response**
**Financial Disclosure**<br><br>Enter a financial disclosure statement that describes the sources of funding for the work included in this submission. Review the submission guidelines for detailed requirements. View published research articles from *PLOS ONE* for specific examples.<br><br>This statement is required for submission and **will appear in the published article** if the submission is accepted. Please make sure it is accurate.	The author(s) received no specific funding for this work.

Powered by Editorial Manager® and ProduXion Manager® from Aries Systems Corporation

**Unfunded studies**<br>Enter: *The author(s) received no specific funding for this work.*<br><br>**Funded studies**<br>Enter a statement with the following details:<br>• Initials of the authors who received each award<br>• Grant numbers awarded to each author<br>• The full name of each funder<br>• URL of each funder website<br>• Did the sponsors or funders play any role in the study design, data collection and analysis, decision to publish, or preparation of the manuscript?<br>• **NO** - Include this sentence at the end of your statement: *The funders had no role in study design, data collection and analysis, decision to publish, or preparation of the manuscript.*<br>• **YES** - Specify the role(s) played.<br><br>* typeset	
**Competing Interests**<br><br>Use the instructions below to enter a competing interest statement for this submission. On behalf of all authors, disclose any competing interests that could be perceived to bias this work—acknowledging all financial support and any other relevant financial or non-financial competing interests.<br><br>This statement **will appear in the published article** if the submission is accepted. Please make sure it is accurate. View published research articles from *PLOS ONE* for specific examples.	The authors have declared that no competing interests exist.

**NO authors have competing interests**<br><br>Enter: *The authors have declared that no competing interests exist.*<br><br>**Authors with competing interests**<br><br>Enter competing interest details beginning with this statement:<br><br>*I have read the journal's policy and the authors of this manuscript have the following competing interests: [insert competing interests here]*<br><br>* typeset	
**Ethics Statement**<br><br>Enter an ethics statement for this submission. This statement is required if the study involved:<br><br>• Human participants<br>• Human specimens or tissue<br>• Vertebrate animals or cephalopods<br>• Vertebrate embryos or tissues<br>• Field research<br><br>Write "N/A" if the submission does not require an ethics statement.<br><br>General guidance is provided below. Consult the submission guidelines for detailed instructions. **Make sure that all information entered here is included in the Methods section of the manuscript.**	N/A

**Format for specific study types**<br><br>**Human Subject Research (involving human participants and/or tissue)**<br>• Give the name of the institutional review board or ethics committee that approved the study<br>• Include the approval number and/or a statement indicating approval of this research<br>• Indicate the form of consent obtained (written/oral) or the reason that consent was not obtained (e.g. the data were analyzed anonymously)<br><br>**Animal Research (involving vertebrate animals, embryos or tissues)**<br>• Provide the name of the Institutional Animal Care and Use Committee (IACUC) or other relevant ethics board that reviewed the study protocol, and indicate whether they approved this research or granted a formal waiver of ethical approval<br>• Include an approval number if one was obtained<br>• If the study involved *non-human primates*, add *additional details* about animal welfare and steps taken to ameliorate suffering<br>• If anesthesia, euthanasia, or any kind of animal sacrifice is part of the study, include briefly which substances and/or methods were applied<br><br>**Field Research**<br><br>Include the following details if this study involves the collection of plant, animal, or other materials from a natural setting:<br>• Field permit number<br>• Name of the institution or relevant body that granted permission	
**Data Availability**<br><br>Authors are required to make all data underlying the findings described fully available, without restriction, and from the time of publication. PLOS allows rare exceptions to address legal and ethical concerns. See the PLOS Data Policy and FAQ for detailed information.	Yes - all data are fully available without restriction

Powered by Editorial Manager® and ProduXion Manager® from Aries Systems Corporation

A Data Availability Statement describing where the data can be found is required at submission. Your answers to this question constitute the Data Availability Statement and **will be published in the article**, if accepted.<br><br>**Important:** Stating 'data available on request from the author' is not sufficient. If your data are only available upon request, select 'No' for the first question and explain your exceptional situation in the text box.<br><br>Do the authors confirm that all data underlying the findings described in their manuscript are fully available without restriction?	
**Describe where the data may be found in full sentences. If you are copying our sample text, replace any instances of XXX with the appropriate details.**<br><br>• If the data are **held or will be held in a public repository**, include URLs, accession numbers or DOIs. If this information will only be available after acceptance, indicate this by ticking the box below. For example: *All XXX files are available from the XXX database (accession number(s) XXX, XXX.*).<br>• If the data are all contained **within the manuscript and/or Supporting Information files**, enter the following: *All relevant data are within the manuscript and its Supporting Information files.*<br>• If neither of these applies but you are able to provide **details of access elsewhere**, with or without limitations, please do so. For example:<br><br>*Data cannot be shared publicly because of [XXX]. Data are available from the XXX Institutional Data Access / Ethics Committee (contact via XXX) for researchers who meet the criteria for access to confidential data.*<br><br>*The data underlying the results presented in the study are available from (include the name of the third party*	The data underlying the results presented in the study are available in the Supporting Information File S1File.xlsx.

and contact information or URL).<br>• This text is appropriate if the data are owned by a third party and authors do not have permission to share the data.<br><br>* typeset	
Additional data availability information:	

Powered by Editorial Manager® and ProduXion Manager® from Aries Systems Corporation

Manuscript

Click here to access/download;Manuscript;GrowthBirds_resubmitted_v4.doc

Bertalanffy-Pütter models for avian growth

Norbert Brunner[1], Manfred Kühleitner[1*], and Katharina Renner-Martin[1]

1 University of Natural Resources and Life Sciences (BOKU), Department of Integrative Biology and Biodiversity Research (DIBB), A-1180 Vienna, Austria

* Corresponding author

E-Mail: manfred.kuehleitner@boku.ac.at

All authors contributed equally to this work.

Abstract

This paper explores the ratio of the mass in the inflection point over asymptotic mass for 81 nestlings of blue tits and great tits from an urban parkland in Warsaw, Poland (growth data from literature). The observed ratios ranged from 0 (non-sigmoidal growth for six birds) to 0.835. We computed the ratios using the Bertalanffy-Pütter model, because for other models there were a-priori restrictions on their possible range: For 19 birds the ratio was between 0.1 and 0.368 (lowest ratio attainable for the Richards model) and for 48 birds the ratio was above 0.5 (fixed ratio of logistic growth). With these ratios we were able to detect small variations in avian growth due to slight differences in the environment: For blue tits, a higher light pollution and a higher percentage of impervious area was associated to a lower ratio, which meant a slower growth.

Key words and abbreviations: *Cyanistes caeruleus*, *Parus major*, sum of squared logarithmically transformed errors (*SSLE*), Bertalanffy-Pütter model (BP-model)

Introduction

This paper uses nonlinear regression models to study the growth of passerine birds, blue tits (*Cyanistes caeruleus*) and great tits (*Parus major*). Ricklefs [1] was amongst the first to recommend simple regression models for exploring the growth patterns of nestlings. For example, the S-shaped (sigmoidal) growth curves of the logistic trend model [2-3] were used to relate growth to predation [4-5], to environmental variation [6-8], to annual adult mortality rates, and to incubation period duration [9]. Literature considered various environmental factors that may affect growth, such as: tree cover [10], impervious surface [11-12], pollution by light [13] and sound [14-16] or nest interference by humans and pets [17]. All these factors are known to affect the breeding success, too. When clearly distinct environments were compared (example: forests and urban parklands), then significant differences in the breeding success of blue tits [17] and in the growth of nestlings [8] could be established by logistic growth and other simple models.

In this paper we aimed at demonstrating that less distinct environmental variation and the ongoing adaption of birds to urban environments [18] at a small scale could potentially be detected with more complex models. We decided to use the five-parameter Bertalanffy-Pütter

regression model (BP-model) for this purpose, because it generalizes the above-mentioned three-parameter regression models and ~~because it~~ provides a common biophysical interpretation for them (explained in the methods below). The growth function $m(t)$ of the BP-model describes mass (m) at time (t) using the following differential equation of Pütter [19].

$$m'(t) = p \cdot m(t)^a - q \cdot m(t)^b \quad (1)$$

The model parameters of equation (1) are to be determined from fitting the model to mass-at-age data: Four parameters are displayed in the equation, namely the non-negative exponent-pair $a < b$ and the scaling constants p and q. An additional parameter is the initial value (intuitively the hatching mass), i.e. $m(0) = c > 0$. This model was recently recommended in epidemiology [20-22] and it was also proposed [23] and applied for studies of animal growth, where the BP-model (1) achieved significant improvements over the logistic model and other simpler three-parameter models with respect to the fit of the data [24-27]. However, the benefits of its use remained little known and insufficiently evaluated.

Our study was based on data from [12, 28-30] ~~that informed about~~ the development of 81 nestlings of blue tits and great tits, and ~~about~~ the environmental characteristics of their nest sites (nest-boxes) in a public park of the Warsaw city center. ~~(Details about the data are explained in the methods.)~~

For the growth data of each nestling we identified the best-fit parameters for the BP-model~~.~~ ~~We used it to smoothen the growth data of the individual birds by means of the model curves. Note that the BP-model~~ (necessarily) ~~has~~ a better fit ~~to the data~~ than the traditional three-parameter models~~,~~ ~~but this~~ improved fit was not the reason for using this model~~. (~~As [31] argued, a better fit alone would not justify the added efforts of more complex models.~~)~~ Rather, we used the BP-model as it allowed to compute certain shape-parameters (related to the shape of the growth trajectory) ~~that could be defined for smooth curves, only,~~ such as the inflection point and its ratio over the asymptotic size, ~~and~~ because it was flexible with respect to the

possible values of these parameters. For, a major concern about logistic growth and other simple three-parameter models was the inflexibility with respect to the shape of the growth curve [32]. Using statistical analysis, we then explored whether the shape-parameter ratio, as derived from the BP-model, could reveal potential impacts of the environment on avian growth that were unobserved with other models.

Methods

BP and related models

In temporal order the most common three-parameter growth models in avian research [33] are the Gompertz [34] model, the logistic model of Verhulst [2], the monomolecular model (bounded exponential growth) of Brody [35], the von Bertalanffy [36-37] model, and the more recent West model [38-39]. There are also simpler trend models, such as power-laws between size and age [40], and more complex models explaining growth in relation to food consumption [41] or describing spatial characteristics of growth by partial differential equations [42].

The BP-model generalizes several common models in the following sense. If an exponent-pair (a, b) is preset, then equation (1) defines a model $BP(a, b)$ with three parameters (c, p and q). It is a special case of the general BP-model. The above-mentioned models of Bertalanffy and West are $BP(2/3, 1)$ and $BP(3/4, 1)$, respectively, monomolecular growth of Brody is $BP(0, 1)$, and logistic growth is $BP(1, 2)$. The Gompertz model is the limit case $BP(1, 1)$ with a distinct differential equation, where b converges to $a = 1$ from above [43]. Common four-parameter models fit into this scheme, too: [37] suggested the BP-model with $b = 1$ ("generalized Bertalanffy model" with free parameters $0 \leq a < 1$, p, q, and the initial value), Richards [44] model is the BP-model with $a = 1$ (free parameters $b > 1$, p, q, and the initial value).

Assuming exponents $a = 1$ or $b = 1$, as for the above-mentioned models, then the differential equation (1) could be solved by means of elementary functions [37, 44-46]. For general exponent-pairs the solution of differential equation (1) was expressed in terms of the Gaussian hypergeometric function ${}_2F_1$ [43, 47], which is not an elementary function [48].

[37] interpreted equation (1) as a model of ontogenetic growth, where the body would utilize resources at a metabolic rate ($p \cdot m^a$) for growth, except for the resources allocated to the operation and maintenance of existing tissue ($q \cdot m^b$). Different biophysical explanations for growth translated into different exponent-pairs. (Literature refers to them as “metabolic exponents”.) Thus, [36] proposed the exponents $a = 2/3$, as lung surface (assumed to be proportional to the 2/3rd power of mass) would delimit oxygen uptake, and $b = 1$, as cell count (assumed to be proportional to mass) would determine the resources needed for maintenance. This model is common in fisheries analysis, where the length-at-age of fish is described by *BP*(0, 1); assuming an allometric relation (mass is proportional to the third power of length) this model for length is equivalent to *BP*(2/3, 1) for mass growth [37]. [39] developed a different argument in support of the exponents $a = 3/4$ and $b = 1$ for avian growth. This model was often used for mammalian growth [49]. Other authors stressed that the metabolic exponents would also depend on the environment and not merely on biophysics [50-51]. Hence, it would be meaningful to identify the best-fit exponent-pair.

Shape-parameter

A main advantage of using the BP-model over the “named” three-parameter models was its flexibility due to two additional parameters (the variable exponent-pair). Owing to the larger pool of available shapes of the growth curves we did expect better estimates of the inflection point. For solutions of equation (1), the asymptotic mass m_{max} and the mass m_{infl} at the inflection point (peak growth, reached at time t_{infl}) are computed from equation (2).

$$m_{max} = \left(\frac{p}{q}\right)^{\frac{1}{b-a}}, \quad m_{infl} = \left(\frac{a}{b}\right)^{\frac{1}{b-a}} \cdot m_{max} \quad (2)$$

For $q = 0$ asymptotic mass is infinite and there is no inflection point (examples: linear growth $a = 0$, unbounded exponential growth $a = 1$). For $a = 0$ there is no inflection point, either (example: Brody model). For birds without inflection point we recorded $m_{infl} = 0$ and $t_{infl} = 0$. For the general model (1) the age t_{infl} at the inflection point could only be determined numerically (solving $m(t) = m_{infl}$ for t). Note that an infinite (or unreasonably large) asymptotic mass did not always mean a poor fit of the growth curve to the data. Rather, the growth process might have been truly unbounded, as suggested for *Drosophila* larvae [37], or the data could have been representative for the initial (exponential) phase of growth, only.

This paper explores the ratio m_{infl}/m_{max}, whence flexible models allowing to fit these parameters to the data were preferable for us. For example, in view of equation (2) the ratio did depend on the exponent-pair, only. Therefore, for any given three-parameter models $BP(a, b)$ with a preset exponent-pair, the ratio was fixed, irrespective of the data to which the model was fitted. For instance, logistic growth always displays the ratio $m_{infl}/m_{max} = 0.5$ (unless $q = 0$). The four-parameter Richards-model ($a = 1$, b variable) was more flexible. However, for this model the ratio (dependent solely on b) was bounded from below by $1/e$ (ca. 0.368), whereas [37] argued that for vertebrates the true ratio would be significantly smaller, namely around 1/3. (For the generalized Bertalanffy model with $b = 1$ and variable $a < b$, $1/e$ was the upper bound for the ratios.) The higher flexibility was our motive to use the general BP-model, for which any ratio between 0 and 1 could be realized.

The parameters m_{max}, m_{infl}, and t_{infl} are known to have a biological meaning. Asymptotic mass was linked to adult mass [52]. The inflection point was related to a change in diet, from spiders with much keratin (for the growth of feathers) to protein-rich caterpillars [53]. Further,

the maximal growth rate, i.e. the derivative $m'(t_{infl})$, was suggested as a proxy for the basal metabolic rate [54].

The ratio, too, might be biologically relevant, as it was defined from two biologically meaningful parameters. When we compared two BP-growth curves with the same initial value and the same asymptotic limit (example: Figure 1), then the curve with a higher ratio had a higher inflection point, whence (from its t_{infl} on) it was sooner close to the asymptotic limit. Thus, the ratio indicated, how fast the asymptotic size was approached finally. When we compared the ratio with the environmental indicators, we interpreted Figure 1 as follows and sought for supporting evidence: Initially the growth trajectories differed slightly, but at a later phase birds from disadvantaged neighborhoods felt the environmental deterioration (e.g., their parents did not find enough food) and their growth was inhibited, explaining a lower ratio.

Bird and nest-box data

~~This paper is based on~~ life history data from [28-30] and environmental data from [12] that were collected between March and June of 2016. [30] investigated the development of nestlings of blue tits and great tits that grew up at various sites in and around Warsaw, Poland. [12] complemented these growth data by environmental data from the nest-box sites. We received from these authors data about 429 nestlings from 56 nest-boxes. For each bird, data included the ID of the bird (ring number) and of the nest-box, the species (blue tits or great tits), the hatching date, the number of hatchlings at the first visit, mass in gram (eight repeated weighing), sex (F or M), and fledging success. To distinguish birds prior to ringing, they were marked at the first measurement.

To ensure comparability, we studied only a fraction of the 429 nestlings. First, to exclude irregular growth patterns from runts, we selected only birds whose sex could be determined (F or M) and that finally ~~became~~ fledged. ~~(We used these criteria as a proxy for healthy birds.)~~

Second, we selected only birds that were weighed at the same eight-odd numbered days 1 (hatching), 3, 5, 7, … 15. ~~We thereby considered only nestlings that survived that long. (The others were for instance, early fledging, runts, or predated.) As we wished to compare the shapes of the growth curves, we did not consider birds that were weighed at other days, even if they survived.~~ Third, we selected only hatchlings from the first brood. ~~They grew up during May and June. (According to open data from Warsaw airport, the month of May was dry with cool nights and June was warm and humid.)~~ This reduced the count of nestlings to 95 birds in 24 nests at two study sites: Kampinos national park (4 nest-boxes with 14 nestlings) and Pole Mokotowskie park close to the city center (20 nest-boxes with 81 nestlings). ~~Fourth~~, for a statistical comparison of growth in relation to the environment, we removed the 14 Kampinos data. ~~Otherwise, a rural-urban comparison would depend on merely four rural nests. Further, as for our data rural hatching occurred later, it was likely that food availability and composition differed, but there were no data about the diet.~~

Thus, our study was based on 81 Pole Mokotowskie nestlings. ~~As is evident from a plot of the data (Figure 2),~~ species and sex affected body mass, ~~whence~~ we controlled for these factors: There were 26 female and 31 male blue tits and 12 female and 12 male great tits.

To study the association between environmental variables and growth we used the following information about the nest-boxes ~~that the above-cited authors had retrieved from geo-information systems or collected at the eight visits. The latter data measured~~ human activity [28], air temperature at the nest-box site (average of the morning temperatures in °C at the eight visits), and light and sound pollution at the nest-box site (in ~~lx and db). The former were~~ distances to paths and roads (minimal Euclidean distance in m from the nest-box to the center of the next path or road), percentage of impervious surface around the nest (ISA), tree cover in percent around the nest, and NDVI (normalized difference vegetation index). Methodology details can be found in [12, 28-30].

In addition, we considered the association of growth to brood-specific indicators, namely the above-mentioned hatching day, the count of hatchlings at the first visit (4-12 birds/nest at day 1), the count at the last visit (1-9 birds/nest at day 15), and the difference between these counts. This difference may have been caused amongst others by early fledging, by runts (e.g. adverse impact from the environment) or by predation. In the latter case, the likely predators were woodpeckers, as they did not harvest all nestlings at once. (Nests that were destroyed, e.g. by martens, were removed from the sample.)

Calibration

There are different techniques to fit growth curves to data and to analyze them further. A large body of literature used the method of least squares and variants of it. Other approaches used spline-interpolation [55-56], time series methods [57], or stochastic (partial) differential equations [58]. More recently, mixed-effect models became popular [59-61], but to remain viable they were based on growth curves that could be parameterized by means of elementary functions such as for the Verhulst model [62] or the Richards model [63].

We decided to use a variant of the method of least squares that had worked well for chicken and other dinosaurs [25-26]: We fitted logarithms of BP-growth curves to the logarithmically transformed data. Thereby, given the growth data, we aimed at finding parameters that minimized *SSLE* of equation (3). If $m(t)$ was a solution of equation (1), using certain exponents $a < b$ and parameters p, q, c, and if (t_i, m_i) were the mass-at-age data (for our data: $n = 8$), then *SSLE* was defined by equation (3):

$$SSLE = \sum_{i=1}^{n} \Big(\ln(m_i) - \ln\big(m(t_i)\big)\Big)^2 \quad (3)$$

SSLE was related to a method of weighted least squares using $1/m(t_i)^2$ as weights [64], since its residual, $\ln(m_i)–\ln(m(t_i))$, approximated the relative error, $(m_i–m(t_i))/m(t_i)$.

The logarithmic transformation of the data in equation (3) is an instance of the Box & Cox [65] power transformations that aim at stabilizing variance, making data-fitting less sensitive to higher variances for the higher masses. As can be seen from Figure 2, for our data the spread of the weights of birds of the same species and gender increased with time. Further, shortly before fledging the body mass of birds may reach a peak and then go down or begin to fluctuate [66], whence we expected also for this reason higher variances for higher masses. [5] suggested to truncate the data at the peak, but we did not truncate our data as this could leave not enough points of time to determine the model-parameters. For our data, the fit remained reasonable also for finally decreasing growth trajectories, as illustrated by Figure 3. (The plotted BP-model curve did not deviate much from the data: maximal error 0.37 g. This curve was best fitting with respect to *SSLE*.)

To justify the logarithmic transformation, we checked for each bird, if its data $\ln(m_i)$ were normally distributed (lognormal distribution of body mass) and if the residuals in equation (3) were normally distributed (*SSLE* as a maximum likelihood estimation). We used the Anderson-Darling test (see below).

Data fitting

For the common three-parameter models there are various software solutions for determining the best-fitting parametric growth curves [67-68]. However, literature reported difficulties with data fitting for the generalized Bertalanffy model [69] and for the Richards model [70]; both models require the optimization of four parameters. For the general five-parameter BP-model (1), standard optimization tools in general failed to find the best-fit parameters, even if they started close to them [24]. ~~As for an example of a failure from literature [20], the Levenberg-Marquart algorithm did not converge for the five-parameter BP-model. ([20] reported the following fits for Covid-19 in Shanghai: R-squared for logistic growth was~~

~~88.1%, while it was only 86.7% for the general BP-model. This was obviously false, as the BP-model is more general and therefore it necessarily improves upon logistic growth.)~~

To simplify data-fitting, several authors suggested to reduce the number of free parameters by not optimizing certain parameters. We did not apply this strategy. For instance, many authors defined the parameter $c = m(0)$ by the hatching mass (the first weighing at day 1 corresponded to the age $t = 0$), as this reduced the number of optimized parameters by one [41]. In contrast, we used hatching mass as an estimate for the initial value but allowed the optimization to identify a better value of c to find a lower *SSLE*. [52] recommended to identify asymptotic mass with the average adult age of the considered group of birds. We did not use this extrapolation, as after fledging and prior to attaining adult mass there might have occurred another growth phase, as was observed for farmed birds [71]. Thus, adult mass might overestimate the asymptotic mass of the considered growth phase.~~ (We could not observe an additional growth phase for wild birds, as after leaving the nest-boxes they did not return for weighing.)~~

To resolve these difficulties, for our data we ~~used a~~ grid-search combined with a custom-made variant of the method of simulated annealing [72] for data fitting. Thereby, we defined a grid of exponent-pairs and for each grid-point exponent-pair (a, b) we identified the best-fit growth curve for the three-parameter model $BP(a, b)$ by means of simulated annealing (redesigned to generate positive parameter-values, only). Amongst these growth-curves we then identified the overall best fitting one. We used Mathematica 12 ~~of~~ Wolfram Research for our computations; for details of our approach to optimization and (explanations of) the Mathematica-code we refer to [25-26].

Model comparison

To assess the goodness of fit across different datasets (nestlings), we considered an analogue of the coefficient of determination for *SSLE*, namely RL-squared of equation (4). It assesses the relative improvement of *SSLE* for the best-fit BP-model in comparison to the best-fitting constant model (geometric mean of the masses):

$$RL^2 = 1 - \frac{SSLE(\text{BP model})}{SSLE(\text{constant})} \quad (4)$$

In view of certain limitations of R-squared [73] that apply also to RL^2, we did not draw further conclusions about model selection, as RL-squared would not be sufficiently selective for such a purpose. However, model selection per se was not an issue of this paper, because for conceptual reasons (flexible ratio m_{infl}/m_{max}) we decided to use the five-parameter BP-model, even if there was a danger of overfitting (meaning that for some data simple models, such as logistic growth, might have been more parsimonious [74]).

Utilizing the information from optimization, we aimed at quantifying the possible consequences of overfitting. For each bird we identified those grid-point exponent-pairs (a, b), where the corresponding three-parameter model, $BP(a, b)$, had a good fit to the data in terms of RL-squared. For each of these models we computed the parameter values of interest. We then evaluated the spread by means of the difference between the 95%-quantile and the 5%-quantile of the parameter-values that were computed for this set of models. (For our data, defining a good fit by $RL^2 \geq 95\%$, in the median about 43,000 models per bird ~~were used.)~~

Statistical computations

We used the standard statistical tests of the Mathematica software. For all statistical tests we decided at 95% significance (P-value 5%). We preferred non-parametric methods suitable for small samples [75], such as the one-sided sign test to estimate confidence intervals for the

medians, the Mann-Whitney test to check for significant stochastic differences, or the Spearman rank correlation (rho) together with the Spearman rank test. In case we needed distribution assumptions, we used the Anderson & Darling [76] test to verify them.

We identified the best-fit BP-growth curves for each individual bird. This approach avoided the pooling of masses across individuals (fitting growth curves to average-size-at-age). While pooling would be computationally easier (one fit for all females and males of each species) and the variations of the averages might be smaller (resulting in better fits), the aggregate data may miss biologically important differences between individuals (averaging treats them as equal). For the further analysis of the shape-parameters, we controlled for species and gender. This stratification defined four samples (female and male blue tits, female and male great tits) of moderate sample sizes (12-31 individuals). To keep sample sizes large enough for statistically significant conclusions we did not control for nest sizes~~. (Instead, we~~ used resampling techniques to reduce this influence~~,~~ see below.)

The search for significant associations between the shape-parameter (ratio) and the 13 (brood-specific and environmental) indicators applied the Spearman rho and the Spearman rank test for each of the four samples. This resulted in 52 tests, whence spurious outcomes were to be expected, where one or more of the tests falsely reported a significant correlation. Thereby, we expected at most five spurious outcomes (P-value 4.45% for six or more false reports of “significant”, assuming a binomial distribution with 52 trials and a chance of 5% for errors). To identify the valid associations, we focused on the highly significant ones. For them, we sought for additional supporting evidence by testing the same associations under two different aspects.

For the first additional aspect we asked: Did variations in the local environments (at or around the nest-sites) lead to different shapes of the growth curves of the nestlings? We tested the hypothesis that nestlings from inferior environments (e.g., higher pollution) were inhibited in

their growth. Thereby, we diagnosed a slower growth for these birds if they had stochastically lower ratios than the birds of the other neighborhoods (Mann-Whitney test). As thresholds between inferior and superior environments we used the medians of Table 1 for each indicator and group of birds. Thus, we divided each of the four samples into two groups of approximately equal sizes, namely birds that grew up in an ambience with a high parameter value (equal or larger than the sample median) and the other birds. For these classes we tested for significant differences in the location of the ratio. As for great tits each comparison involved a class with only 3-6 birds, which barely could be representative, we focused on blue tits, only.

For our second additional aspect we explored, if and to what extent the model-parameters did allow to draw inferences about the “social background” of birds: Did birds with distinct growth-patterns (shapes of the growth-curves, ratios) origin from different environments? We defined subsamples of the four groups of birds in terms of the ratio: A bird was high-ratio, if $m_{infl}/m_{max} \geq 0.5$ for the best-fit BP-model growth-curve (the threshold 0.5 comes from logistic growth), and otherwise it was low-ratio. Now, using the Mann-Whitney test we compared the medians of the environmental indicators for the subsamples of high-ratio and low-ratio birds. Thus, we compared the indicator values for the nests of 15 high-ratio female blue-tits with the indicator values for 11 low-ratio female blue tits; the same for 19 high-ratio and 12 low-ratio male tits. Again, the so defined samples for great tits were too small (three high-ratio vs. nine low-ratio female great tits, seven high-ratio vs. five low-ratio male great tits), whence we did not consider them.

Finally, we explored, if the outcomes were affected by correlations between birds of the same nest. There were potentially negative correlations and positive ones (examples: competition for food, common traits inherited from the parents). Further, assuming that growth was affected by the ability of the parents to supply each nestling with adequate food, then this

ability was influenced by nest-size and not only by environmental factors. We used resampling to reduce such dependencies. We selected one bird per nest and repeated the previous analysis of correlations for this small sample (11 female and 11 male blue tits; great tits were not considered). ~~As~~ for these small samples a merely moderate significance could be expected, we gathered evidence by comparing different selection strategies, deterministic (selecting representative birds) or stochastic (bootstrap-sampling).

Results

Best-fit parameters

We first observed that for most birds the logarithmic transformation stabilized variance. This confirmed the suitability of *SSLE*. For each bird we tested if its masses (m_i) were log-normally distributed. The Anderson-Darling test did refute this assumption for six (7.4% of 81) birds (*p*-values below 5%). Further, we tested for each bird, if the fit-residuals for *SSLE* were normally distributed; ~~these residuals were $\ln(m_i)–\ln(m(t_i))$ for the eight data (t_i, m_i) and the best-fit BP-model-curve $m(t)$.~~ Here, the Anderson-Darling test refuted this distribution assumption for five (6.2% of 81) birds. For three birds both distribution assumptions were refuted. We did not discard of these data, as there were only few exceptional birds and for them the fit to the BP-growth curve was still acceptable, as is illustrated by Figure 3.

Next, for each bird we screened the growth curve of the BP-model with optimized parameters to assess its fit to the data. In general, the five-parameter BP-model achieved an excellent fit with median-RL^2 of 99.77%; for 70 of 81 data RL-squared was above 99.5%. However, one bird was exceptional (K7V3278), as the best-fit BP-model did not achieve RL-squared above 95% (namely $RL^2 = 94.2\%$). For this bird, asymptotic mass was excessive. We therefore asked, if overall the data captured both the initial rapid growth and the final flattening of growth. To this end we analyzed the quotient of the asymptotic mass over the maximal

observed mass (in general, mass at day 15): It ranged from 0.89 to $1.4 \cdot 10^{13}$ for the exceptional bird. However, the median was 1.006 with a narrow confidence interval. (Lower and upper confidence limits 0.998 and 1.016, both with P-values 2.24% for one-sided sign tests, adding up to $p = 4.5\%$ or 95.5% confidence for the interval.)

~~Figure 4 displays the best-fit exponent-pairs. (Note that another calibration, such as ordinary least squares, would result in different exponent-pairs.)~~ The pairs appeared to be scattered at random, whereby the exponent *b* was much more variable than the exponent *a*, and we could not observe a concentration near any of the exponent-pairs of the "traditional" three-parameter models (Brody, Bertalanffy, Gompertz, West, Verhulst). ~~A notable observation was the concentration close to the diagonal:~~ 47% of the birds were near-diagonal (exponent-difference smaller than 1.5). For six birds the exponent-pairs were on the line $a = 0$ (no inflection point).

~~We stratified the birds by species and by gender and marked their exponent-pairs by different colors in Figure 4. The medians of the exponent-pairs were (0.495, 2.28), (0.63, 2.31), (0.815, 3.155), and (0.835, 3.69) for female and male blue tits, and female and male great tits, respectively.~~ ~~Again,~~ there was no apparent pattern. The proportion of near-diagonal birds was almost equal amongst all groups of birds: 50%, 48%, 42%, and 42% for female and male blue tits, and female and male great tits, respectively.

~~Using equation (2), we computed the best-fit ratios, m_{infl}/m_{max}, from the best-fit exponents. For our data,~~ the best-fit ratios were large, when compared to the claim [37] that for vertebrates the ratios would be around 1/3. The ratios ranged from 0 ($a = 0$) to 0.835, whereby 0.1 was the least ratio for a sigmoidal bird (meaning one with a sigmoidal best-fit growth curve: $a > 0$). For 21 birds (19 of them sigmoidal) the ratio was smaller than $1/e \approx 0.368$ (the lower bound for the ratios of Richards model and the upper bound for the ratios of the generalized Bertalanffy model) and for 48 birds it was above 0.5 (ratio of logistic growth).

For all four groups of birds, the ratios were significantly greater than Bertalanffy's claim of 1/3 (one-sided sign test for sigmoidal birds: P-value below 1.5% for each group). The median ratios (and 95% confidence bounds) were 0.511 (0.467–0.59) for female blue tits, 0.503 (0.467–0.57) for male blue tits, 0.459 (0.42–0.59) for female great tits, and 0.528 (0.42–0.65) for male great tits. (The confidence limits were computed for the sigmoidal birds using one-sided sign-tests, whose P-values added up to a value between 3.9% and 5.2%.)

Identification of the ratio from the shape

We asked, how closely the ratio, a shape-parameter, was related to the shape: For given growth-data, could the ratio be reconstructed from the shape of their growth trajectory, and if so, how accurate? Note, that we deliberately did not define the term "shape". (Literature used the term shape-parameter without defining "shape" [77]). Rather, we assumed that growth curves of the BP-family with a good fit to the data ought to have about the same shape, and we expected that the ratios of curves with similar shapes should have about the same values.

To study the variability of the ratio, we first identified for each bird the exponent-pairs that defined BP-models with "good" fits, meaning e.g. $RL^2 \geq 95\%$. The green area in Figure 5 plots the exponent-pairs from the initial search grid (yellow), whose three-parameter models $BP(a, b)$ achieved $RL^2 \geq 95\%$ for 80 of 81 data. (For one bird, only a lower RL^2 could be achieved.) Except for Brody, all traditional models achieved this fit, whereby logistic growth was outstanding with RL-squared above 98% for 78 data (red area).

Figure 5 cautions that we could expect only a fuzzy correspondence between the shape and the shape-parameters. For, unless we require an extremely good fit, the variability of the ratio might be high and our expectation about its identification from the inspection of the shape of the growth trajectory might be overly optimistic. For, given any of the 80 birds that allowed for a good fit ($RL^2 \geq 95\%$), there was a large area of exponent-pairs (the green area and

beyond, depending on the bird) that represented alternative models, whose growth curves had a good fit to the growth-data and that therefore had about the same shape. ~~Given any bird and its best-fit BP-model growth-curve, even a significant change in two parameters (the exponent-pair) could almost be offset by changes in the other three parameters without changing the good fit (and thus the shape) of the growth curve significantly.~~

However, the ratio turned out to be somewhat resilient against this variability. Considering models with $RL^2 \geq 95\%$, the spread of the ratio was at least 0.495, but for models with $RL^2 \geq 99.5\%$ the minimal spread was reduced to 0.06. ~~Figure 5 explains this: Requiring a higher RL-squared made the area of the exponent-pairs with a good fit smaller (compare the red and green areas), but not in the same proportion in all directions: It became narrower in the direction of the diagonal, which meant a smaller variability of the ratio (c.f. the plotted contour lines of the ratio).~~

Moreover, the best-fit ratio could be estimated by the median of the ratios of the growth-curves of the models with a "very good" fit: There were 66 birds with an inflection point, whose best-fit model satisfied $RL^2 \geq 99.5\%$. For 47 (=71% of 66) birds the relative error between the best-fit ratio and the median of the ratios for reasonably fitting growth curves (meaning $RL^2 \geq 99.5\%$) was below 10%, and for 47 birds the absolute difference between the best-fit ratio and the median was below 0.05.

Correlations with the ratio

In view of the difficulties with parameter reconstruction, we explored the possible biological meaning of the ratio indirectly: We interpreted the ratio as random variable that was defined for mass-at-age data. (Thus, the ratio was defined as a function on the set of time series. We therefore considered also non-sigmoidal trajectories, setting their ratios zero.) We sought for statistically significant associations between this random variable and factors that literature

considered as relevant for growth. Hence, we searched systematically for significant associations between the ratio, four brood-specific indicators (hatching day, count of hatchlings at the first visit, count at the last visit, and difference between these counts), and nine environmental indicators (human activity, ISA, light pollution, NDVI, distance to a path, distance to a road, sound pollution, morning temperature, and tree cover). We studied four groups of birds independently: female/male blue/great tits.

~~First, we checked for correlations between the ratio, m_{infl}/m_{max}, and the brood-specific and environmental indicators.~~ We found eleven significant correlations (P-value below 5%, ~~using the~~ Spearman rank test) and we expected that at most five of them were spurious (see the methods). For the brood-specific indicators ~~we found~~ five highly significant ~~correlations with the ratio.~~ Initial nest-sizes were negatively correlated with the ratio for female blue tits, female great tits, and male great tits (Spearman rank correlation coefficients and P-values $\rho = -0.517$ with $p = 0.68\%$, $\rho = -0.86$ with $p = 0.03\%$, and $\rho = -0.781$ with $p = 0.27\%$, respectively). The count of lost siblings (nest-size difference) was negatively correlated with the ratio for female and male blue tits ($\rho = -0.555$ with $p = 0.32\%$ and $\rho = -0.814$ with $p = 0.13\%$, respectively). For the environmental indicators, we found six significant correlations, the following four highly significant: For blue tits, a higher percentage of impervious area around the nest-box (ISA) and a higher level of light pollution was negatively correlated to a lower ratio. (For ISA, $\rho = -0.54$ with $p = 0.4\%$ for females and $\rho = -0.48$ with $p = 0.69\%$ for males, and for light pollution $\rho = -0.51$ with $p = 0.35\%$ for males and $\rho = -0.64$ with $p = 0.04\%$ for females.) Two associations were of a lower significance, namely a positive correlation with NDVI (more is better) for male great tits ($\rho = 0.701$ with $p = 1.1\%$) and a negative one with sound pollution for female blue tits ($\rho = -0.43$ with $p = 2.8\%$). The other comparisons were not significant.

The highly significant correlations of the ratio with initial nest size for three groups of birds gave rise to the question if the four highly significant associations of the ratios of blue tits with environmental indicators were insofar artificial, as birds in larger nests might have dominated the computations. We therefore considered resampling, defining subsamples with one bird for each nest. In view of the smaller sample sizes (11 male and female birds, each), we did not expect low P-values.

First, we selected the birds deterministically. If for each nest we selected the bird (of the required gender) with "typical growth" (ratio nearest to the nest-median), then for both groups of female and male blue tits the ratio had a significant negative correlation with light pollution and ISA (P-values below 3%). If we grouped together the fastest-growing birds from each nest (highest ratio), then for females we obtained a significant negative correlation of light pollution with ratio. If we grouped together the birds with the lowest ratio of their nests, then both for females and for males we obtained significant correlations between ratio and ISA and for females also between ratio and light pollution.

Next, we used bootstrap simulations: Given the groups of female and male blue tits, for each group we selected one bird per nest at random and for this sample we tested the associations between the environmental indicators and the ratio. We recorded the Spearman rho together with its P-value and repeated the simulation thousand times. Both for female and male blue tits, and for the tests for a significant correlation between the ratio and ISA or light pollution, Spearman rho was negative for all simulations. Further, for all groups and indicators, with one exception the median of the thousand P-values was below 5%. The exception was the association between ratio and ISA for female blue tits (median P-value 6.3%). The strongest association with the ratio was observed for females with respect to light pollution: $\rho < -0.5$ and $p < 12\%$ for thousand simulations.

Thus, resampling confirmed the significant correlations between growth and the indicators impervious area (ISA) and light pollution. It thereby suggested a stronger evidence for a potential impact from light pollution on growth and a weaker one from ISA.

Further associations with the ratio for blue tits

~~To add supporting evidence for the potential impact of light pollution and impervious area on the growth of blue tits, and check for possible additional associations, we added tests for different aspects of the associations.~~

~~Was the ratio stochastically lower (Mann-Whitney test) in highly polluted environments, than in lowly polluted ones? We asked this for all 13 indicators for the two groups of female and male blue tits.~~ We found five significant associations, at most four of them spurious. (This was based on a P-value 3.3% for a binomial distribution assuming 26 comparisons and a 5% chance of a false significance.) Nestlings of blue tits that grew up in environments with high percentage of impervious area (ISA) or a high level of light pollution had stochastically lower ratios, whereby one test was highly significant. (In detail, for birds from nests with high and low ISA, the median ratios were 0.473 and 0.595, respectively, for males and 0.467 and 0.529, respectively, for females, with $p = 0.316\%$ for males and $p = 3.49\%$ for females. For high and low light pollution the median ratios were $0.493 < 0.576$ for females and $0.491 < 0.599$ for males with $p = 4.15\%$ for females and $p = 1.57\%$ for males.) Further, male blue tits from nests with a higher NDVI (better environment) had a significantly higher ratio.

Was the level of environmental pollution stochastically lower near the nests of high-ratio birds than near those of low-ratio birds? We identified seven significant associations, at most four of them spurious (see above). High-ratio blue tits likely grew up in environments with less light pollution and a lower ISA. (Specifically, the medians of ISA were $4.54\% < 13.33\%$ with $p = 1.18\%$ for high-ratio and low-ratio females, and $3.58\% < 8.88\%$ with $p = 1.14\%$ for

high-ratio and low-ratio males. For light pollution we observed the medians 2852 lx < 3160 lx with p = 2.0% for males and 2896 lx <5937 lx with p = 4.1% for females.) Further, high-ratio females originated likely from smaller nests (median initial sizes 9 and 10.5, p = 1.2%) with less sound pollution (medians 64 db < 68 db, p = 0.87%) and fewer siblings lost (median nest-size differences 3 and 6 with p =0.43%).

Related issue

The various brood-specific and environmental indicators were not independent. In particular, nest-size difference (between the initial and final counts) was associated to several environmental indicators.

Testing for 12 indicators, we observed 18 significant correlations, of them expecting at most six spurious ones (P-value 3.2% for six or more false tests amongst 48 ones, each with a 5% chance of errors). Blue tits displayed highly significant negative correlations (P-values close to zero) of nest-size difference with the final count and significant positive correlations with the initial count and the hatching day. With respect to the environmental indicators, for blue tits there were highly significant positive correlations of nest-size difference with ISA ($\rho = 0.565$ with p = 0.26% for females, $\rho = 0.412$ with p = 2.1% for males) and with light pollution (females only: $\rho = 0.548$ with p = 0.37%). However, for great tits, the correlations with ISA were negative, significantly for females ($\rho = -0.676$ with p = 1.6%). Further, nest-size difference was positively correlated for blue tits to sound pollution ($\rho = 0.854$ for females and $\rho = 0.826$ for males with both P-values close to zero), temperature ($\rho = 0.681$ with p = 0.012% for females, $\rho = 0.369$ with p = 4.1% for males), it was negatively correlated for male blue tits to human activity ($\rho = -0.386$ with p = 3.2%) and NDVI ($\rho = -0.529$ with p = 0.22%), and it was positive correlated for females to the distance to a path ($\rho = -0.445$ with p = 2.3% for blue tits, $\rho = 0.676$ with p = 1.6% for great tits).

Discussion and conclusions

We studied environmental indicators from avian literature that potentially affect the growth of birds. ~~Previously such findings were based on comparisons of large samples from clearly distinct environments, such as the urban and rural dichotomy [8, 17]. In our context, the sample size was comparably small, and we expected that the environmental variation across the urban study site (Pole Mokotowskie park) would be gradual. Could we nevertheless identify significant differences in the growth parameters?~~ To find any potential environmental impact, we tested 13 indicators, but ~~this immediately gave rise to the~~ concern of spurious outcomes ~~amongst 52 tests for four groups of birds. We responded to this risk by a three-step testing-strategy. First,~~ we used three different ~~tests for the same associations that utilized different aspects, namely~~ a) correlation tests, b) location tests for the shape-parameter (ratio) of birds from clearly distinct environments, and c) location tests of the environmental parameters for birds with clearly distinct shapes of the growth curves (high ratio vs. low ratio birds). If the same association was supported by three different tests, ~~then it was less likely that it was spurious. Second, we only considered associations that were~~ highly significant for at least one of the three tests, as such associations a priori were less likely to be spurious. ~~And third,~~ extending ~~the idea of multiple support, we focused~~ on associations that were significant for the females and the males of the same species or that were even shared by different species. ~~For such associations we speculated with a biological reason, although it was not the purpose of the paper to detect any causations.~~

For blue tits, we first concluded that ~~most likely~~ there were valid significant statistical associations between some environmental variables and the ratio, m_{infl}/m_{max}. ~~For,~~ the count of the significant associations exceeded the likely count of spurious outcomes in each testing-round ~~(outlined in the results-section)~~ and several associations were highly significant. ~~Thereby, we identified two environmental indicators as outstanding: impervious area (ISA)~~

~~and light pollution.~~ For all three testing approaches, a higher level of urbanization (impervious area, light pollution) was associated to a lower ratio, which in turn indicated a finally slower growth towards the adult (asymptotic) mass. These findings remained stable, when we eliminated the possible dependency on nest-size by resampling with one bird from each nest.

~~Avian literature confirms that~~ these findings were biologically plausible. For instance, impervious area around the nest could inhibit the success of ~~fetching food~~ [11] and this clearly would affect the growth of the nestlings. ~~Further, it has long been known that~~ light pollution affects birds [13]. ~~But now we wondered: Why did only these two environmental differences matter and why did we not observe similar associations with indicators that are known to have strong impacts? We conjecture that environmental factors may have masked certain associations and enhanced, others. Sound pollution might be an example. It~~ is known to affect certain passerine species adversely, as for instance in noisy environments female sparrows reduced the provisioning rate for their brood [16]. For our data, sound pollution had a highly significant positive correlation with nest-size difference, but the evidence for an impact of sound pollution on the growth of the nestlings was weak. ~~(For female blue tits, only, we observed a significant negative correlation of sound pollution with the ratio and high-ratio birds were likely to have come from less noisy nests.) We speculate that sound pollution might have affected breeding success adversely (higher nest-size difference) and this in turn might have mitigated the impact on growth. (Assuming, that a shrinking nest-size might increase the per capita food supply for the nestlings, the reduction of nest-size could have saved the surviving birds from starvation.)~~

~~As to a more technical issue, we observed that~~ for about half of the birds the best-fit exponent-pair was close to the diagonal. ~~Was there a biological reason for this? This question was the starting point of the present paper, but~~ in view of the high variability (see Figure 5) of the exponent-difference (*b*–*a*) we ~~did not arrive at conclusive answers~~. ~~(For blue tits, the~~

~~potential impact of light pollution was supported again: significant negative correlation with the exponent-difference.) We therefore sought for a function on the exponent-pairs with a lower variability; this was the~~ ratio.

We conclude, irrespective of the biological interpretations of our results, ~~that we could verify that computing~~ the ratio ~~by means~~ of the BP-model was a suitable instrument to detect small variations in avian growth due to ~~slight differences in the environment.~~ As the ratio is meaningful for any model, researchers interested in studying the relation of the ratio to environmental indicators are not confined to the BP-model. ~~Yet, as noted in the introduction and pointed out also by [78], the used~~ model must be flexible with respect to the ratio, ~~whence~~ the more traditional models (logistic growth) are not suitable for this task~~, even if their fit might be good (as for the logistic model for most of the present data).~~ It is of interest, if other classes of models that are flexible with respect to the ratio provide similar outcomes.

Learning from the limitations of the present study, sample size was an issue in several aspects. First, to allow for more refined stratifications much larger samples of birds would be desirable. For great tits, the above described second and third round of tests required further stratifications, but then the sample sizes became too small to draw meaningful conclusions and consequently we could not identify differences between the species in their responses to environmental challenges. Owing to the interventions by predators, one would require a huge sample at the beginning (for the present research, the data of 81 birds were used, but five times as many nestlings were observed, initially) and lots of computing power (with commercial computers, the data fit lasted for up to a week per bird). Second, part of the high variability of the size-parameters was caused by the relatively small number of time-points used. Weighing the birds daily rather than at every second day would have reduced the size of the green region in Figure 5.

Acknowledgements

This paper originated from conversations with Michela Corsini and Eva Maria Schöll, who explained to us the methodology of the papers [12, 28-30]. The data for these papers were collected under projects directed by Marta Szulkin as the principal investigator. She gave us the permission to publish the data as a supporting information (part of S1File) for the present paper. As this research was ethically approved, there occurred no ethical issues for this paper.

The authors are grateful to Michela Corsini and Júlio Neto, who provided insightful comments on preliminary versions of our paper. Further, the authors appreciate the extensive comments by Steve Oswald and Tales J. Fernandes, the two reviewers of this journal. They helped to improve the paper substantially.

Supporting Information

S1File.xlsx: Excel file with explanations of the columns in rows 1-2. Columns A-D provide the bird IDs together with general information, E-Q provide the brood-specific and environmental data for each nestling (and nest), R-Y list the bird-mass at days 1 to 15 (odd days, only), and AA-AH provide for each bird the best-fit parameters of its five-parameter BP-model together with additional information about the model.

References

[1] Ricklefs R.E. A graphical method of fitting equations to growth curves. Ecology 1967, 48: 978-983.
[2] Verhulst P.F. Notice sur la loi que la population suit dans son accroissement. Curr. Math. Phys. 1838, 10:113-121.
[3] Pearl R., Reed L.J. On the rate of growth of the population of the United States since 1790 and its mathematical representation. Proceedings of the National Academy of Sciences USA 1920, 6: 275-288.
[4] Ricklefs R.E.) The Optimization of Growth Rate in Altricial Birds. Ecology 1984, 65: 1602-1616.
[5] Remeš V., Martin T.E. Environmental influences on the evolution of growth and developmental rates in passerines. Evolution 2002, 56: 2505–2518.
[6] Bleu J, Agostini S, Biard C Nest-box temperature affects clutch size, incubation initiation, and nestling health in great tits. Behavioral Ecology 2017, 28: 793-802.
[7] Ndithia H., Bakari S., Matson K., Muchai M., Tieleman I. Geographical and temporal variation in environmental conditions affects nestling growth but not immune function in a year-round breeding equatorial lark. Frontiers in Zoology 2017, 14, published online: DOI 10.1186/s12983-017-0213-1.
[8] Remacha C., Delgado J.A., Bulaic M., Pérez-Tris J. Human disturbance during early life impairs nestling growth in birds inhabiting a nature recreation area. PLoS ONE 2016, 11, published online: DOI 10.1371/journal.pone.0166748.
[9] Remeš V. Avian growth and development rates and age- specific mortality: the roles of nest predation and adult mortality. Evolutionary Biology 2007, 20: 320-325.
[10] Kirschel A.N., Blumstein D.T., Cohen R.E., Buermann W., Smith T.B., Slabbekoorn H. Birdsong tuned to the environment: green hylia song varies with elevation, tree cover, and noise. Behavioral Ecology 2009, 20: 1089-1095.
[11] Juárez R., Chacón‐ Madrigal E., Sandoval L. Urbanization has opposite effects on the territory size of two passerine birds. Avian Research 2020, published online: DOI 10.1186/s40657-020-00198-6.

[12] Szulkin M., Garroway C.J., Corsini M., Kotarba A.Z., Dominoni D. How to quantify urbanization when testing for urban evolution? In: Szulkin M., Munshi-South J., Charmantier A. (Eds.), Urban Evolutionary Biology. Oxford University Press 2020, in print.

[13] Da Silva A., Samplonius J.M., Schlicht E., Valcu M., Kempenaers B. Artificial night lighting rather than traffic noise affects the daily timing of dawn and dusk singing in common European songbirds. Behavioural Ecology 2014, 25: 1037-1047.

[14] Francis C.D. Vocal traits and diet explain avian sensitivities to anthropogenic noise. Global Change Biology 2015, 21: 1809-1820.

[15] Halfwerk W., Holleman L.J., Lessells C.K., Slabbekoorn H. Negative impact of traffic noise on avian reproductive success. Journal of Applied Ecology 2011, 48: 210-219.

[16] Schroeder J., Nakagawa S., Cleasby I.R., Burke T. Passerine birds breeding under chronic noise experience reduced fitness. PloS ONE 2012, 7, published online: DOI 10.1371/journal.pone.0039200.

[17] Gladalski M., Banbura M., Kalinski A., Markowski M., Skwarska J., Wawrzyniak J., Zielinski P., Cyzewska I., Mankowska D., Banbura J. Effects of human-related disturbance on breeding success of urban and non-urban blue tits (Cyanistes caeruleus). Urban Ecosystems 2016, 19, published online: DOI 10.1007/s11252-016-0543-3.

[18] Bańbura J., Bańbura M. Blue tits Cyanistes caeruleus and great tits Parus major as urban habitat breeders. Inter Studies Sparrows 2012, 36: 66-72.

[19] Pütter A. Studien über physiologische Ähnlichkeit. VI. Wachstumsähnlichkeiten. Pflügers Archiv für die Gesamte Physiologie des Menschen und der Tiere 1920, 180: 298-340.

[20] Wu K., Darcet D., Wang Q., Sornette D. Generalized logistic growth modeling of the COVID-19 outbreak in 29 provinces in China and in the rest of the world. Nonlinear Dynamics 2020, published online: DOI 10.1007/s11071-020-05862-6.

[21] Zreiq R, Kamel S, Boubaker S, Al-Shammary AA, Algahtani FD, Alshammari F. Generalized Richards model for predicting COVID-19 dynamics in Saudi Arabia based on particle swarm optimization algorithm. AIMS Public Health 2020, 7: 828-843.

[22] Brunner N., Kühleitner M. Forecasting the final disease size: comparing calibrations of Bertalanffy–Pütter models. Epidemiology and Infection 2021, 149, published online: DOI 10.1017/S0950268820003039.

[23] Pauly D., Cheung W.W.L. Sound physiological knowledge and principles in modeling shrinking of fishes under climate change. Global Change Biology 2018, 24, published online: DOI 10.1111/gcb.13831.

[24] Renner-Martin K., Brunner N., Kühleitner M., Nowak W.G., Scheicher K. On the Exponent in the Von Bertalanffy Growth Model. PeerJ 2018, 6, published online: DOI 10.7717/peerj.4205.

[25] Renner-Martin K., Brunner N., Kühleitner M., Nowak W.G., Scheicher K. Best-fitting Growth Curves of the Von Bertalanffy-Pütter Type. Poultry Science 2019, 98: 3587-3592.

[26] Brunner N., Kühleitner M., Nowak W.G., Renner-Martin K., Scheicher K. Comparing growth patterns of three species: Similarities and differences. PLoS ONE 2019, 14, published online: DOI 10.1371/journal.pone.0224168.

[27] Brunner N., Kühleitner M. The growth of domestic goats and sheep: A meta study with Bertalanffy-Pütter models. Veterinary and Animal Science 2020, 10, published online: DOI 10.1016/j.vas.2020.100135.

[28] Corsini M., Dubiec A., Marrot P., Szulkin M. Humans and Tits in the City: Quantifying the Effects of Human Presence on Great Tit and Blue Tit Reproductive Trait in Variation. Frontiers in Ecology and Evolution 2017, 5, published online: DOI 10.3389/fevo.2017.00082.

[29] Corsini M., Marrot P., Szulkin M. Quantifying human presence in a heterogeneous urban landscape. Behavioral Ecology 2019, 30: 1632-1641.
[30] Corsini M., Schöll E.M., Di Lecce I., Chatelain, M., Dubiec A., Szulkin M. Growing in the city: urban evolutionary ecology of avian growth rates. Evolutionary Applications 2020, published online: DOI 10.1111/eva.13081.
[31] Zach R. Growth-curve analysis: A critical reevaluation. Auk 1988, 105: 208-210.
[32] Tjørve K.M.C., Tjørve E. Shapes and functions of bird-growth models: how to characterize chick postnatal growth. Zoology 2010, 113: 326-333.
[33] Kuhi H.D., Porter T., López S., Kebreab E., Strathe A., Dumas A., France J. A review of mathematical functions for the analysis of growth in poultry. Worlds Poultry Science Journal 2010, 66: 227-240.
[34] Gompertz B. On the nature of the function expressive of the law of human mortality, and on a new mode of determining the value of life contingencies. Philos. Trans. R. Soc. London 1832, 123: 513-585.
[35] Brody S. Bioenergetics and Growth. Hafner Publ. Comp. 1945, New York, NY, USA.
[36] Bertalanffy L.v. Problems of organic growth. Nature 1949, 163: 156-158.
[37] Bertalanffy L.v. Quantitative laws in metabolism and growth. Quarterly Revues in Biology 1957, 32: 217-231.
[38] West G.B., Brown J.H., Enquist B.J. A general model for ontogenetic growth. Nature 2001, 413:628-631.
[39] West G.B., Brown J.H., Enquist B.J. Growth models based on first principles or phenomenology? Functional Ecology 2004, 18: 188-196.
[40] Katsanevakis S., Maravelias C.D. Modelling fish growth: multi-model inference as a better alternative to a priori using von Bertalanffy equation. Fish and Fisheries 2008, 9:178-187.
[41] Manabe A., Yamakawa T., Ohnishi S., Akamine T., Narimatsu Y., Tanaka H., Funamoto T., Ueda Y., Yamamoto T. A novel growth function incorporating the effects of reproductive energy allocation. PLoS ONE 2018, 13, published online: DOI 10.1371/journal.pone.0199346
[42] Jacobs J., Rockne R.C., Hawkins-Daarud A.J., Jackson P.R., Johnston S.K., Kinahan P., Swanson K.R. Improved model prediction of glioma growth utilizing tissue-specific boundary effects. Mathematical Biosciences 2019, 312: 59-66.
[43] Marusic M., Bajzer Z. Generalized Two-Parameter Equations of Growth. Journal of Mathematical Analysis and Applications 1993, 179: 446-462.
[44] Richards F.J. A flexible growth function for empirical use. Journal of Experimental Botany 1959, 10: 290-300.
[45] Tjørve K.M.C., Tjørve E. Modelling avian growth with the Unified-Richards: As exemplified by wader-chick growth. Journal of Avian Biology 2017, 48: 770-784.
[46] Fernandes F.A., Silva E.M., Lima K.P., Jane S.A., Fernandes T.J., Muniz J.A. Parameterizations of the von Bertalanffy model for description of growth curves. Revista Brasileira de Biometrica 2020, 38: 369-384.
[47] Ohnishi S., Yamakawa T., Akamine T. On the Analytical Solution for the Pütter-Bertalanffy Growth Equation. Journal of Theoretical Biology 2014, 343: 174-177.
[48] Erdélyi A., Magnus, W., Oberhettinger F., Tricomi F.G. Higher transcendental functions I. McGraw-Hill Book Company 1953, New York, USA.
[49] Fernandes F.A., Fernandes T.J., Pereira A.A., Meirelles S.L.C., Costa A.C. Growth curves of meat-producing mammals by von Bertalanffy's model. Pesquisa Agropecuária Brasileira 2019, 54, published online: DOI 10.1590/s1678-3921.pab2019.v54.01162.
[50] Stewart J., Robbins W.D., Rowling K., Hegarty A., Gould A. A multifaceted approach to modelling growth of the Australian bonito, Sarda australis (Family Scombridae), with

some observations on its reproductive biology. Marine and Freshwater Research 2013, 64: 671-678.
[51] White C.R. Physiology: there is no single p. Nature 2010, 464: 691-693.
[52] Austin S.H., Robinson T.R., Robinson W.D., Ricklefs R.D. Potential biases in estimating the rate parameter of sigmoid growth functions. Methods in Ecology and Evolution 2011, 2: 43-51.
[53] Naef-Daenzer B., Keller L.K. The foraging performance of great and blue tits (Parus major and P. caeruleus) in relation to caterpillar development, and its consequences for nestling growth and fledging weight. Journal of Animal Ecology 1999, 68: 708-718.
[54] Calder WA III Size, Function, and Life History. Harvard Univ. Press 1985, Cambridge, MA, USA.
[55] Aggrey S.E. Comparison of three nonlinear and spline regression models for describing chicken growth curves. Poultry Science 2002, 81:1782-1788.
[56] Maceina M.J. Use of piecewise nonlinear models to estimate variable size-related mortality rates. North American Journal of Fisheries Management 2007, 27:971-977.
[57] Hsing Y. Economic growth and income inequality: the case of the US. International Journal of Social Economics 2005, 32: 639-647.
[58] Garcia O. A stochastic differential equation model for the height growth of forest stands. Biometrics 1983, 39: 1059-1072.
[59] Pinheiro J.C., Bates D.M. Mixed-Effects Models in S and S-Plus. Springer Publishing 2000, New York, USA.
[60] Serroyen J., Molenberghs G., Verbek G., Davidian M. Non-linear Models for Longitudinal Data. American Statistician 2009, 63: 378-388.
[61] Strathe A.B., Danfær A., Sørensen H., Kebreab E. A multilevel nonlinear mixed effects approach to model growth in pigs. Journal of Animal Science 2010, 88: 638-649.
[62] Sofaer H.R., Chapman P.L., Sillett T.S., Ghalambor C.K. Advantages of nonlinear mixed models for fitting avian growth curves. Journal of Avian Biology 2013, 44: 469-478.
[63] Svagelj W.S., Laich A.G., Quintana F. Richards's equation and nonlinear mixed models applied to avian growth: why use them? Journal of Avian Biology 2019, 50, published online: DOI 10.1111/jav.01864.
[64] Gustavsson S., Fagerberg B., Sallsten G., Andersson E.M. Regression models for log-normal data: comparing different methods for quantifying the association between abdominal adiposity and biomarkers of inflammation and insulin resistance. International Journal of Environmental Research and Public Health 2014, 11: 3521-3539.
[65] Box G.E.P., Cox, D.R. An analysis of transformations. Journal of the Royal Statistical Society 1964, B26: 211-252.
[66] Brown W.P., Eggermont P., LaRiccia V., Roth R.R. Are parametric models suitable for estimating avian growth rates? Journal of Avian Biology 2007, 38: 495-506.
[67] Ogle D.H. Introductory Fisheries Analyses with R. CRC Press 2016, Boca Raton, FL, USA.
[68] Oswald S.A., Nisbet I.C.T., Chiaradia A., Arnold J.M. FLEXPARAMCURVE: R package for flexible fitting of nonlinear parametric curves. Methods in Ecology and Evolution 2012, 3: 1073-1077.
[69] Shi P.J., Ishikawa T., Sandhu H.S., Hui C., Chakraborty A., Jin X.S., Tachihara K., Li B.L. On the 3/4-exponent van Bertalanffy equation for ontogenetic growth. Ecological Modelling 2014, 276: 23-28.
[70] Loibel S., Andrade M.G., do Val J.B.R., de Freitas A.R. Richards growth model and viability indicators for populations subject to interventions. Anais da Academia Brasileira de Ciências 2010, 82, published online: DOI 10.1590/S0001-37652010000400028.

[71] Grossman M. Multiphasic analysis of growth curves in chicken. Poultry Science 1988, 67: 33-42.

[72] Vidal R.V.V. Applied Simulated Annealing. In: Lecture notes in economics and mathematical systems. Springer Verlag 1993, Berlin, Germany.

[73] Spiess A.N., Neumeyer N. An evaluation of R^2 as an inadequate measure for nonlinear models in pharmacological and biochemical research: a Monte Carlo approach. BMC Pharmacology 2010, 10, published online: DOI 10.1186/1471-2210-10-6.

[74] Burnham K.P., Anderson D.R. Multi-model inference. Understanding AIC and BIC in model selection. Sociological Methods and Research 2004, 33: 261-304.

[75] Hollander M.A., Wolfe D.E. Nonparametric Statistical Methods, Wiley 1999, Chichester, UK.

[76] Anderson T.W., Darling D.A. Asymptotic theory of certain "goodness-of-fit" criteria based on stochastic processes. Annals of Mathematical Statistics 1952, 23: 193-212.

[77] Brisbin Jr.I.L., Collins C.T., White G.C., McCallum D.A. A new paradigm for the analysis and interpretation of growth data: The shape of things to come. Auk 1987, 104: 552-554.

[78] Vrána J., Remeš V., Matysioková B., Tjørve K.M.C., Tjørve E. Choosing the right sigmoid growth function using the unified-models approach. Ibis 2019, 161: 13-26.

Table

Table 1. Medians of the environmental data for each group of birds and counts of above/below median birds.

Indicator	Female blue tits	count[12]	Male blue tits	count[12]	Female great tits	Male great tits
Hatching day[1]	38 d	A: 15, B: 11	38 d	A: 24, B: 7	36 d	38 d
Hatchlings initially[2]	9	A: 20, B: 6	7	A: 25, B: 6	7	7
Hatchlings finally[2]	6	A: 15, B: 11	5	A: 17, B: 14	5	4
Nest-size difference[2]	5	A: 17, B: 9	1	A: 23, B: 8	1	1
Human activity[3]	1.1	A: 15, B: 11	1.05	A: 20, B: 11	1.1	0.75
ISA[4]	4.59%	A: 13, B: 13	4.49%	A: 20, B: 11	10.38%	9.07%
Light[5]	3160 lx	A: 14, B: 12	2852 lx	A: 20, B: 11	8588 lx	4907 lx
NDVI[6]	0.76	A: 14, B: 12	0.75	A: 17, B: 14	0.69	0.71
Path[7]	14.3 m	A: 11, B: 15	11.1 m	A: 17, B: 14	5.55 m	6.9 m
Road[8]	85.4 m	A: 15, B: 11	165.1 m	A: 18, B: 13	63.7 m	106.9 m
Sound[9]	65.44 db	A: 13, B: 13	65.93 db	A: 16, B: 15	65.41 db	66.98 db
Temperature[10]	10.86°C	A: 17, B: 9	10.86°C	A: 20, B: 11	11.04°C	11.08°C
Tree cover[11]	28.13%	A: 14, B: 12	19.07%	A: 16, B: 15	30.46%	24.90%

Notes: All numbers were rounded to the shown decimal. [1] count of days from the start of the field work to hatching; [2] initial and final counts, respectively, of hatchlings in each nest, and difference of these counts; [3] index of human activity from Corsini et al. (2017); [4] percentage of impervious area around the nest-box; [5] light pollution at the nest-box site (in lx); [6] index for the vegetation cover around the nest-box; [7] minimal (Euclidean) distance to the (center of the) next path (in m); [8] minimal (Euclidean) distance from the nest-box to (the center of) the next road (in m); [9] sound pollution at the nest-box site (in db); [10] average of the morning temperatures at the eight visits (in °C); [11] tree cover around the nest-box (percent); [12] count of birds of the given type (first row, left column), whose nest characteristics was above (A)/below (B) the median (displayed in the left column). Counts for great tits are not displayed, because for 12 birds per group the subsamples were too small to be representative.

Figure captions

Figure 1: Growth trajectories of logistic growth and an alternative growth model together with the inflection points. The growth curves had the parameters a = 1, b = 2, c = 0.1, p = q = 1 (blue: logistic growth) and a = 1, b = 4, c = 0.1, p = q = 0.7526 (red). The asymptotic limit was 1 (both curves) and the inflection point coordinates (gray dots) were (2.197, 0.5) for logistic growth and (2.572, 0.63) for the alternative curve.

Figure 2: Mass at days 1, 3, ..., and 15 plotted side-by-side; from left blue tits-F (red), blue tits-M (blue), great tits-F (orange), and great tits-M (cyan).

Figure 3: Mass of bird K7V3513 at days 1, 3, ..., and 15 (plotted at t = 0, 1, ..., 14) and best-fitting BP-model growth curve (parameters a = 0.8, b = 3.8, c = 1.01, p = 0.4, q = 0.0007) with SSLE = 0.006.

Figure 4: Best-fit exponent-pairs for 81 nestlings ~~(color code as in Figure 1)~~ and yellow lines (b = a and b = a+1.5) to indicate near-diagonal exponent-pairs.

Figure 5: Exponent-pairs of named models (blue), exponent-pairs of models with RL > 98% for 78 data (red), exponent-pairs of models with RL > 95% for 80 data (green), remaining exponent-pairs of the search grid (yellow), diagonal a = b (grey) and contour lines (thick and black) of the ratios 0.2, 0.3, ..., 0.6 (from left to right), using equation (2) for the ratio.

Figure1

Click here to access/download;Figure;Figure1new_comparison.tif

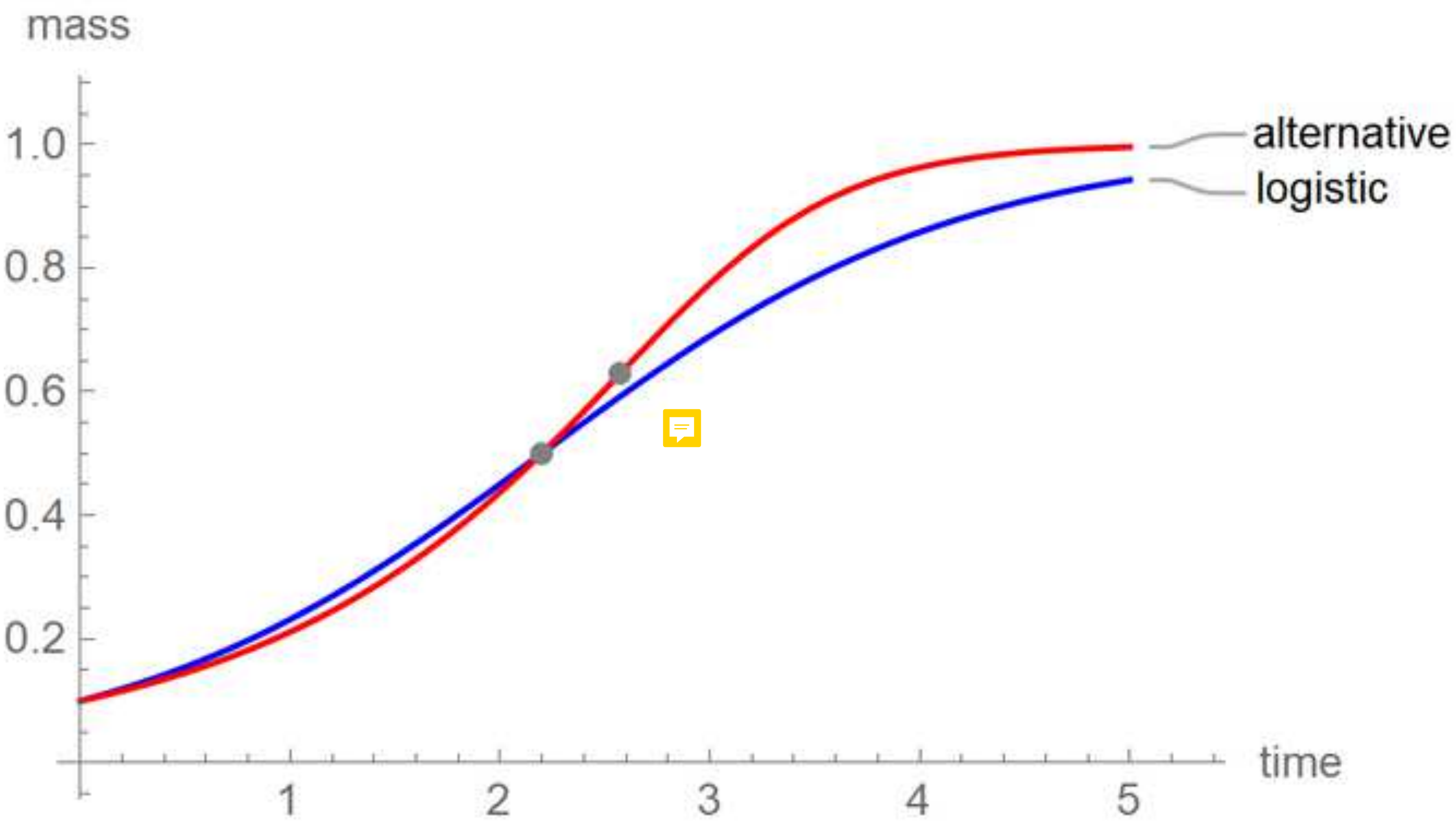

Figure2 Click here to access/download;Figure;Figure2new_data.tif

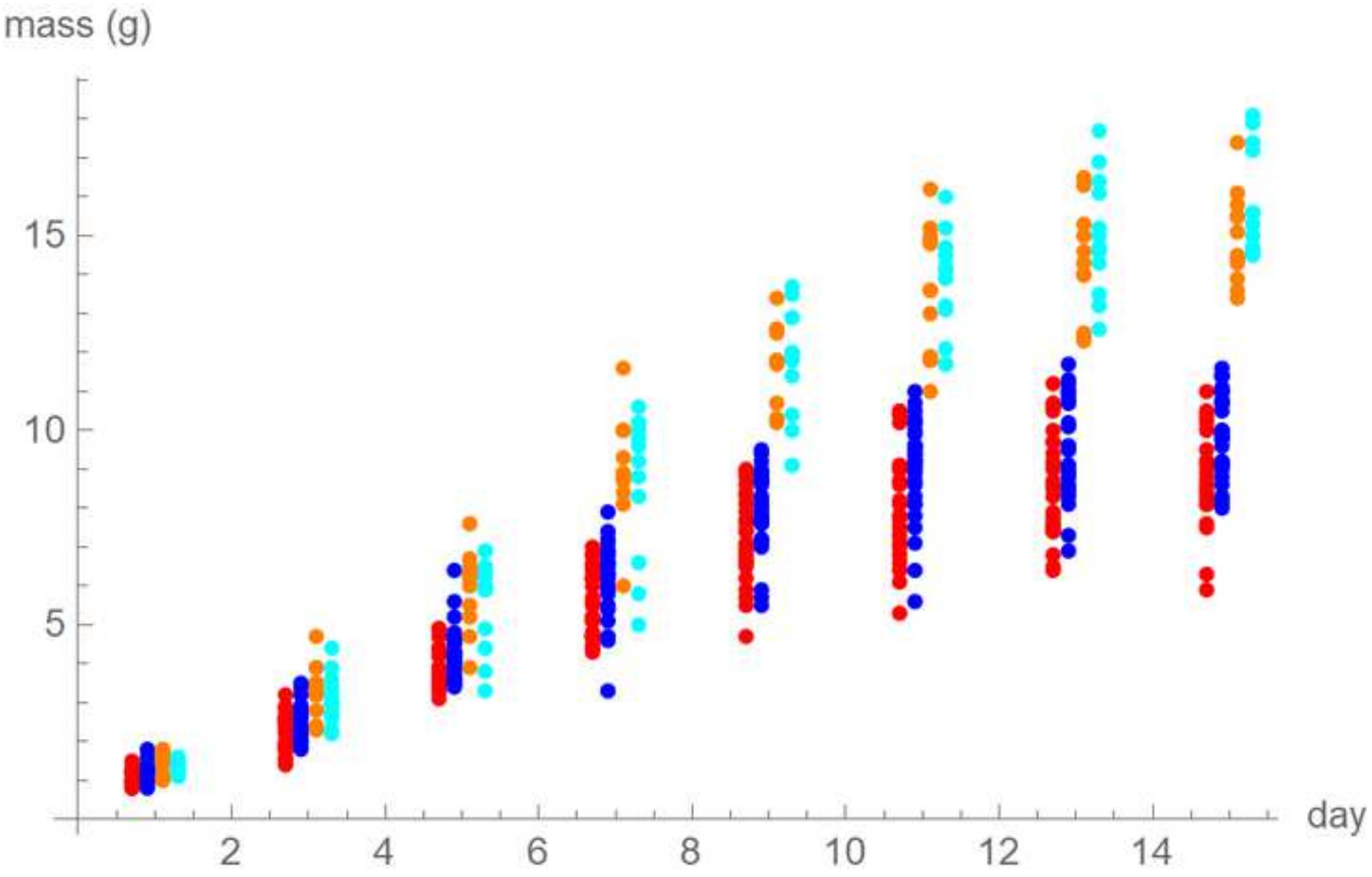

Figure3

Click here to access/download;Figure;Figure3new_DataBestfit.tiff

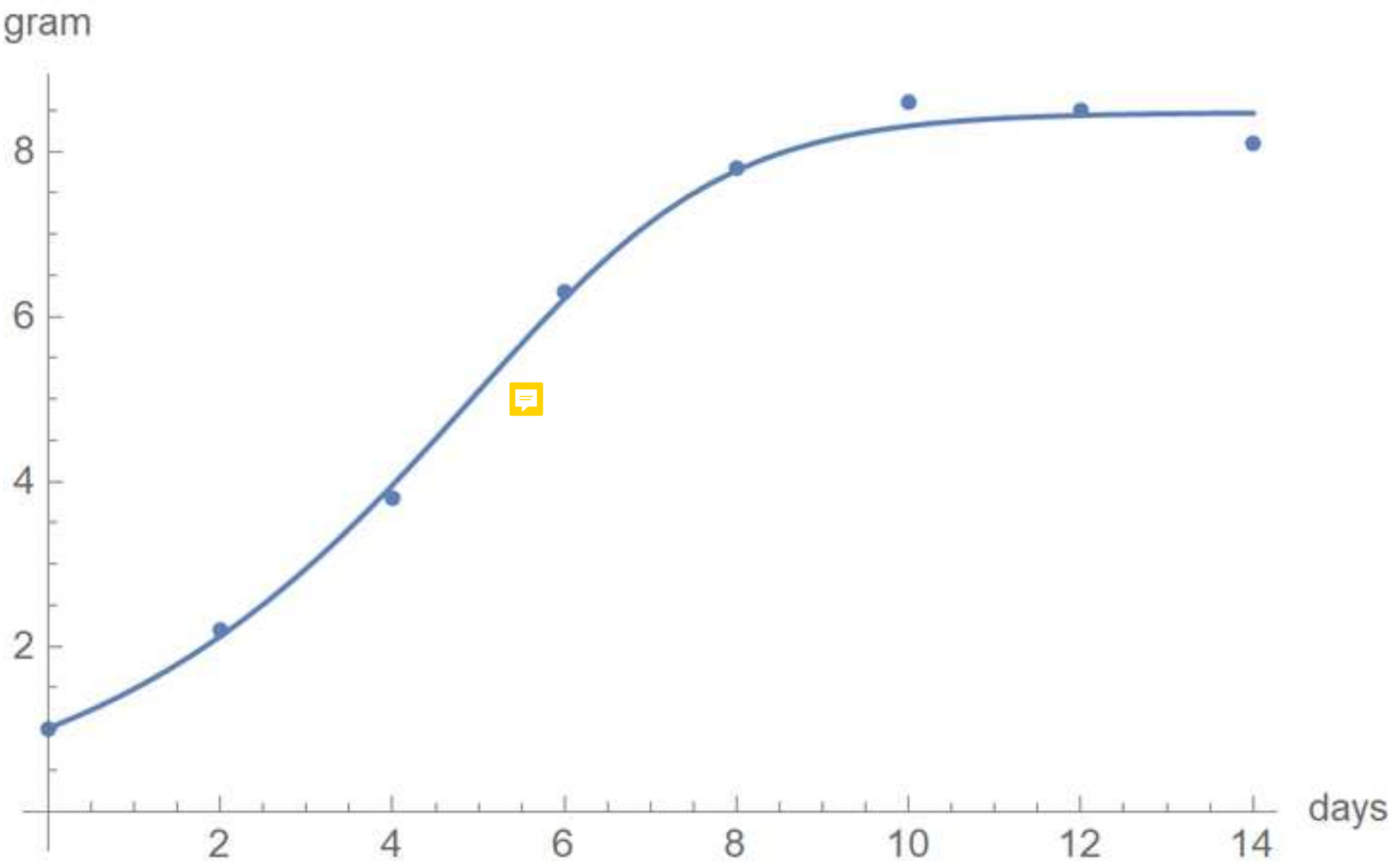

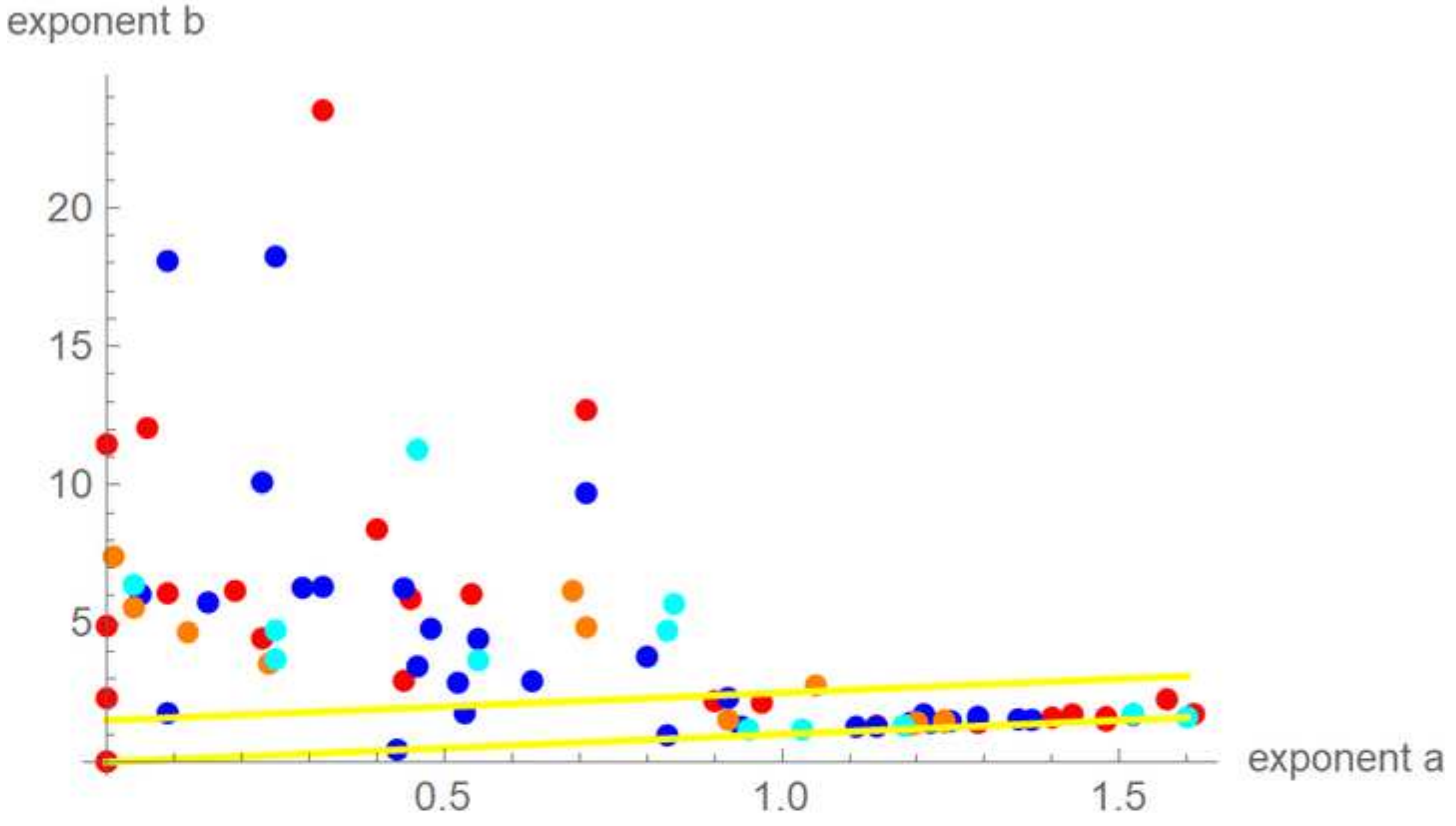
exponent b
20
15
10
5
0.5
1.0
1.5
exponent a

Figure5

Click here to access/download;Figure;Figure5new_AcceptableModels.tif

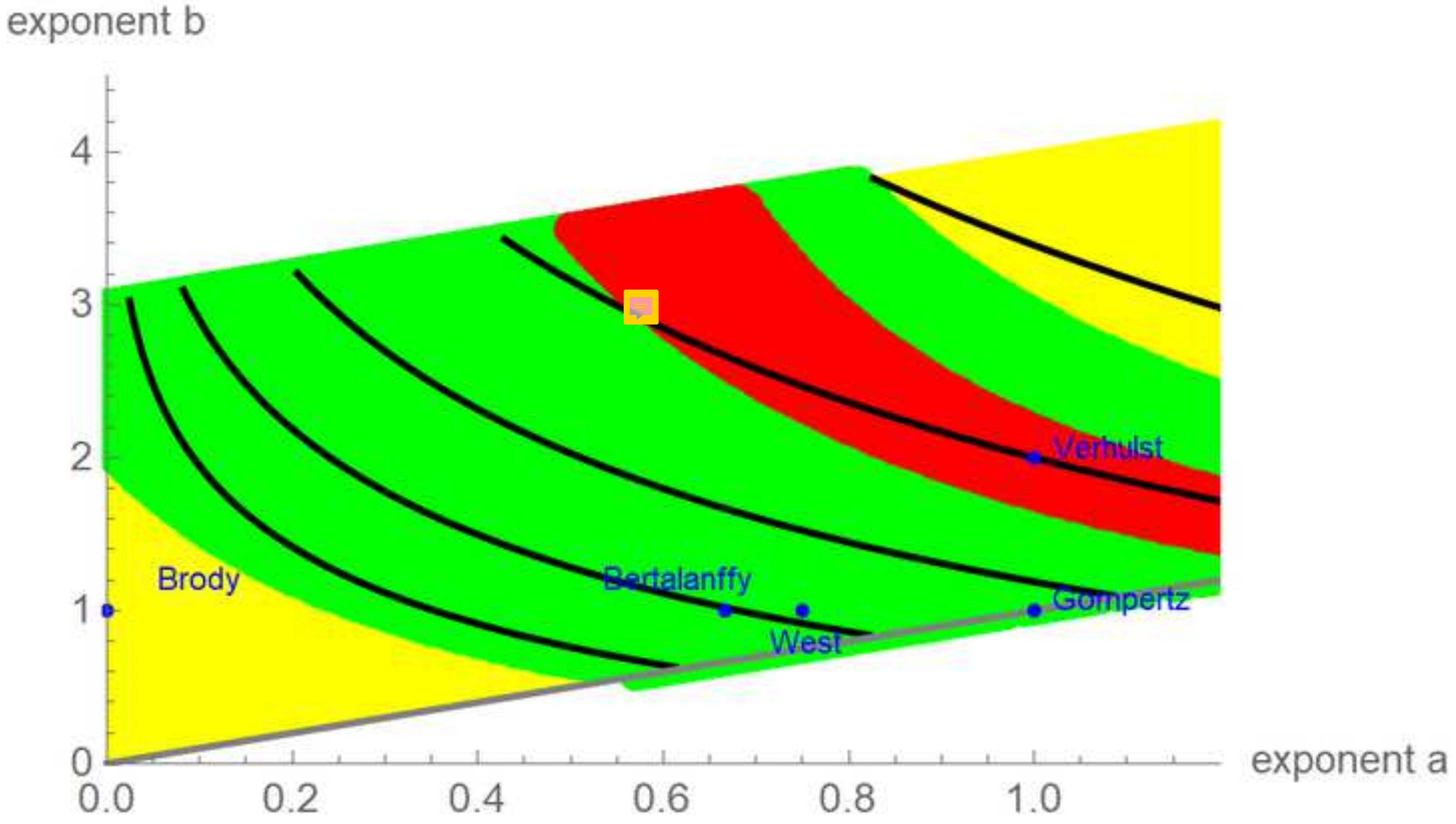

Click here to access/download
Supporting Information
S1File.xlsx

Title: Bertalanffy-Pütter models for avian growth

Style Definition: Heading 1: Font: 18 pt

Style Definition: Heading 2: Font: 16 pt, Bold, Not Italic, Line spacing: 1.5 lines

Style Definition: No Spacing: Font: Arial Narrow, 9 pt

Formatted: Heading 1, Centered, Line spacing: Double

Short title: Models for avian growth

Authors:

Norbert BrunnerBrunner[1], Manfred KühleitnerKühleitner[1]*, and Katharina Renner-MartinMartin[1]

Formatted: German (Austria)

Formatted: Line spacing: Double

Formatted: German (Austria)

Formatted: German (Austria)

Formatted: German (Austria)

Affiliation of all authors:

1 University of Natural Resources and Life Sciences (BOKU),

Department of Integrative Biology and Biodiversity Research (DIBB),

A-1180 Vienna, Austria

Formatted: Normal, Indent: Left: 0", Hanging: 0.49"

E-mail of all authors:

norbert.brunner@boku.ac.at, manfred.kuehleitner@boku.ac.at, kathi.renner-martin@gmx.de

ORCID of authors:

NB: 0000-0001-6487-5752, MK: 0000-0002-0217-7550, KRM: 0000-0002-5733-2546

*

* Corresponding author: Manfred Kühleitner

Institute of Mathematics, DIBB, BOKU

Gregor Mendel Strasse 33, A-1180 Vienna, Austria

Formatted: Normal, Indent: Left: 0", Hanging: 0.49"

Formatted: Font: Times New Roman, Not Bold

E-Mail: manfred.kuehleitner@boku.ac.at

Formatted: Font: Times New Roman, English (United States)

Formatted: Font: Times New Roman, English (United States)

Formatted: No underline, English (United States)

Formatted: English (United States)

Formatted: Line spacing: Double

Models for avian growth: page2

Bertalanffy-Pütter models for avian growth

All authors contributed equally,Abstract. The logistic model is widely used in avian growth literature, but it is inflexible with respect to this work.

Formatted: Font: Not Bold

Abstract

This paper explores the ~~shape~~ratio of the ~~growth curve. For instance, it assumes a constant ratio 0.5 of the size of~~ mass in the inflection point over ~~the~~ asymptotic ~~size. The Bertalanffy-Pütter (BP) model is more versatile in this respect, as it has two additional parameters. We explored whether certain shape parameters for the growth curves of the BP-model (e.g. the above-mentioned ratio) could be used to detect life history impacts from environmental variation. To this end we used literature data for the growth of~~ mass for 81 nestlings of blue tits ~~(BT)~~ and great tits ~~(GT) from nestlings~~ from an urban parkland in Warsaw, Poland~~. We identified the best-fit BP-models to these data and found significant associations~~ (growth data from literature). The observed ratios ranged from 0 (non-sigmoidal growth for six birds) to 0.835. We computed the ratios using the Bertalanffy-Pütter model, because for other models there were a-priori restrictions on their possible range: For 19 birds the ratio was between ~~the shape-parameters (computed from the best-fit models~~0.1 and 0.368 (lowest ratio attainable for the Richards model) and ~~the environmental condition at the nests~~for 48 birds the ratio was above 0.5 (fixed ratio of logistic growth). With these ratios we were able to detect small variations in avian growth due to slight differences in the environment: For blue tits, a higher light pollution and a higher percentage of impervious area was associated to a lower ratio, which meant a slower growth.

Key words~~: passerine birds,~~ and abbreviations: *Cyanistes caeruleus*, *Parus major*,~~metabolic exponents, life history, growth model, Bertalanffy-Pütter differential equation, simulated annealing~~

~~**Abbreviations**: *BP model*: Bertalanffy-Pütter model; *BT*: blue tits; *F*: female; *GT*: great tits; *M* male; *SSLE*:~~ sum of squared logarithmically transformed errors (*SSLE*), Bertalanffy-Pütter model (BP-model)

Formatted: Line spacing: single

Formatted: Font: Not Bold

Formatted: Font: Italic

Formatted: Font: Italic

Formatted: No Spacing

Introduction

Formatted: Line spacing: Double

This paper uses nonlinear regression models to study the growth of passerine birds~~. In temporal order, the most common three-parameter growth models in avian research (Kuhi et al., 2010) are the Gompertz (1832) model, the logistic model of Verhulst (1838), the monomolecular model (bounded exponential growth) of Brody (1945), the von Bertalanffy (1949) model,~~, blue tits (*Cyanistes caeruleus*) and ~~the more recent model by West et al. (2001). There are also simpler trend models, such as power-laws between size and age (Katsanevakis & Maravelias, 2008), and more complex models aiming at explaining growth in relation to e.g. food consumption (Manabe et al., 2018).~~

great tits (*Parus major*). Ricklefs ~~(1967)~~[1] was amongst the first to recommend simple regression models for exploring the growth patterns of nestlings. For example, the S-shaped (sigmoidal) growth curves of the logistic trend model ~~was~~[2-3] were used to relate growth to predation ~~(Ricklefs, 1984),~~[4-5], to environmental variation [6-8], to annual adult mortality rates, and to incubation period duration ~~(Remeš, 2007), and to environmental variation (Bleu et al., 2017; Ndithia et al., 2017; Remacha et al., 2016). Thereby, various authors~~[9]. Literature considered ~~e.g.~~various environmental factors that may affect growth, such as: tree cover ~~(Kirschel et al., 2009),~~[10], impervious surface [11-12], pollution by light [13] and sound ~~(Da Silva et al, 2014; Francis, 2015; Halfwerk et al, 2011)~~[14-16] or nest interference by humans and pets ~~(Gladalski et al., 2016).~~[17]. All these factors are known to affect the breeding success~~ of birds~~, too. When clearly distinct environments were compared~~, e.g.~~ (example: forests and urban parklands~~,~~), then significant differences in the breeding success of ~~BT (Gladalski et al., 2016)~~blue tits [17] and in the growth of ~~BT~~ nestlings ~~(Remacha et al., 2016)~~[8] could be established by logistic growth and other simple models.

In this paper we aimed at demonstrating that less distinct environmental variation and the ongoing adaption of birds to urban environments ~~(Bańbura & Bańbura, 2012)~~[18] at a small scale could potentially be detected with more complex models. We decided to use the five-parameter Bertalanffy-Pütter regression model (BP-model) for this purpose, because it generalizes the above-mentioned three-parameter regression models and because it provides a

Formatted: Line spacing: Double

common biophysical interpretation for them (explained in the methods below). The growth function $m(t)$ of the BP-model describes mass (m) at time (t) using the following differential equation of Pütter [19].

There are many different types of complex growth models, such as (incomplete list) spline-interpolation (Aggrey, 2002; Maceina, 2007), GARCH and similar time series methods (Hsing, 2005), stochastic (partial) differential equations (Garcia, 1983; Jacobs et al., 2019) or mixed-effect models (Pinheiro & Bates, 2000; Serroyenet al., 2009; Strathe et al., 2010). We decided to use the five-parameter Bertalanffy-Pütter regression model (BP-model) because it generalizes the above-mentioned commonly used three-parameter regression models and because it provides a common biophysical interpretation (see below) for them. The growth function $m(t)$ of the BP-model describes mass (m) at time (t) using the following differential equation (Pütter, 1920). It can be solved analytically, but in general not by means of elementary functions (Ohnishi et al., 2014).

$$(1) \quad m'(t) = p \cdot m(t)^a - q \cdot m(t)^b$$

$$m'(t) = p \cdot m(t)^a - q \cdot m(t)^b \quad (1)$$

The model parameters of equation (1) are to be determined from fitting the model to mass-at-age data: Four parameters are displayed in the equation, namely the non-negative exponent-pair $a < b$ (literature refers to them as "metabolic exponents") and the theand the scaling constants p and q. An additional parameter is the initial value (intuitively the hatching mass), i.e. $m(0) = c > 0$. This model was recently recommended in epidemiology [20-22] and it was also proposed [23] and applied for studies of animal growth, where the BP-model (1) achieved significant improvements over the logistic model and other simpler three-parameter models with respect to the fit of the data [24-27]. However, the benefits of its use remained little known and insufficiently evaluated.

Our study was based on data from [12, 28-30] that informed about the development of 81 nestlings of blue tits and great tits, and about the environmental characteristics of their nest sites (nest-boxes) in a public park of the Warsaw city center. (Details about the data are explained in the methods.)

For the growth data of each nestling we identified the best-fit parameters for the BP-model. We used it to smoothen the growth data of the individual birds by means of the model curves. Note that the BP-model (necessarily) has a better fit to the data than the traditional three-parameter models, but this improved fit was not the reason for using this model. (As [31] argued, a better fit alone would not justify the added efforts of more complex models.) Rather, we used the BP-model as it allowed to compute certain shape-parameters (related to the shape of the growth trajectory) that could be defined for smooth curves, only, such as the inflection point and its ratio over the asymptotic size, and because it was flexible with respect to the possible values of these parameters. For, a major concern about logistic growth and other simple three-parameter models was the inflexibility with respect to the shape of the growth curve [32]. Using statistical analysis, we then explored whether the shape-parameter ratio, as derived from the BP-model, could reveal potential impacts of the environment on avian growth that were unobserved with other models.

Methods

Formatted: Line spacing: Double

~~Each~~BP and related models

In temporal order the most common three-parameter growth models in avian research [33] are the Gompertz [34] model, the logistic model of Verhulst [2], the monomolecular model (bounded exponential growth) of Brody [35], the von Bertalanffy [36-37] model, and the more recent West model [38-39]. There are also simpler trend models, such as power-laws between size and age [40], and more complex models explaining growth in relation to food consumption [41] or describing spatial characteristics of growth by partial differential equations [42].

The BP-model generalizes several common models in the following sense. If an exponent-pair (a, b) is preset, then equation (1) defines a ~~distinct~~ model $BP(a, b)$ with three parameters (c, p

Formatted: Line spacing: Double

and q) as). It is a special case: of the general BP-model. The above-mentioned models of Bertalanffy and West are $BP(2/3, 1)$ and $BP(3/4, 1)$, respectively, monomolecular growth of Brody is $BP(0, 1)$, and logistic growth is $BP(1, 2)$. The Gompertz model is the limit case $BP(1, 1)$ with a distinct differential equation, where b converges to a = 1 from above (Marusic & Bajzer, 1993 = 1 from above [43]. Common four-parameter models fit into this scheme, too: [37] suggested the BP-model with $b = 1$ ("generalized Bertalanffy model" with free parameters $0 \leq a < 1$, p, q, and the initial value). Richards [44] model is the BP-model with $a = 1$ (free parameters $b > 1$, p, q, and the initial value).

Bertalanffy (1957)Assuming exponents $a = 1$ or $b = 1$, as for the above-mentioned models, then the differential equation (1) could be solved by means of elementary functions [37, 44-46]. For general exponent-pairs the solution of differential equation (1) was expressed in terms of the Gaussian hypergeometric function ${}_2F_1$ [43, 47], which is not an elementary function [48].

[37] interpreted equation (1) as a model of ontogenetic growth, where the body would utilize resources at a metabolic rate ($p{\cdot}m^a$) for growth, except for the resources allocated to the operation and maintenance of existing tissue ($q{\cdot}m^b$). Different biophysical explanations for growth translated into different exponent-pairs. (Literature refers to them as "metabolic exponents.".) Thus, Bertalanffy (1949)[36] proposed the exponents a = = 2/3, as lung surface (assumed to be proportional to the 2/3th3rd power of mass) would delimit oxygen uptake, and b = = 1, as cell count (assumed to be proportional to mass) would determine the resources needed for maintenance. West et al. (2004)This model is common in fisheries analysis, where the length-at-age of fish is described by $BP(0, 1)$; assuming an allometric relation (mass is proportional to the third power of length) this model for length is equivalent to $BP(2/3, 1)$ for mass growth [37]. [39] developed a different argument in support of the exponents a = = 3/4 and b = = 1 for modeling avian growth. This model was often used for mammalian growth

Formatted: Line spacing: Double

[49]. Other authors stressed that the metabolic exponents would also depend on the environment and not merely on biophysics ~~(e.g. Stewart et al., 2013; White, 2010), whence~~[50-51]. Hence, it would be meaningful to identify the best-fit exponent-pair.

~~In previous studies~~Shape-parameter

A main advantage of ~~animal growth,~~using the BP-model ~~(1) achieved significant improvements~~ over ~~e.g.~~ the ~~logistic model with respect to the fit of the data (e.g. Brunner et al., 2019; Renner-Martin et al., 2018 and 2019; Pauly & Cheung, 2018), but the benefits of its use remained little known and insufficiently evaluated. A major concern about simple~~"named" three-parameter models was ~~the inflexibility with respect to the shape of the growth curve (Tjørve & Tjørve, 2010). Thereby, a main advantage of using~~ its flexibility due to two additional parameters ~~was the added flexibility for estimating the location~~(the variable exponent-pair). Owing to the larger pool of available shapes of the growth curves we did expect better estimates of the inflection point~~ in relation to asymptotic size~~. For solutions of equation (1), the asymptotic mass m_{max} and the mass m_{infl} at the inflection point (peak growth, reached at time t_{infl}) are computed from equation (2).

$$m_{max} = \left(\frac{p}{q}\right)^{\frac{1}{b-a}}, \quad m_{infl} = \left(\frac{a}{b}\right)^{\frac{1}{b-a}} \cdot m_{max} \quad (2)$$

For q ~~=~~ = 0 asymptotic mass is infinite and ~~for $a = 0$~~ there is no inflection point.~~ Further, other than for the above-mentioned three-parameter models (Tjørve & Tjørve, 2017), for~~ (examples: linear growth $a = 0$, unbounded exponential growth $a = 1$). For $a = 0$ there is no inflection point, either (example: Brody model). For birds without inflection point we recorded $m_{infl} = 0$ and $t_{infl} = 0$. For the general model (1) the age t_{infl} at the inflection point could only be determined numerically (solving $m(t) = m_{infl}$ for t). Note that an infinite (or unreasonably large) asymptotic mass did not always mean a poor fit of the growth curve to the data. Rather, the growth process might have been truly unbounded, as suggested for

Formatted: Font: Not Italic, Not Superscript/ Subscript

Formatted: Font: Not Italic, Not Superscript/ Subscript

Formatted: Line spacing: Double

Drosophila larvae [37], or the data could have been representative for the initial (exponential) phase of growth, only.

$$(2)\quad m_{max} = \left(\frac{p}{q}\right)^{\frac{1}{b-a}}, \quad m_{infl} = \left(\frac{a}{b}\right)^{\frac{1}{b-a}} \cdot m_{max}$$

Both parameters have a biological meaning. The maximal growth rate, i.e. the derivative $m'(t_{infl})$, was suggested as a proxy for the basal metabolic rate (Calder, 1985). Further, theThis paper explores the ratio m_{infl}/m_{max}, whence flexible models allowing to fit these parameters to the data were preferable for us. For example, in view of equation (2) the ratio did depend on the exponent-pair, only. Therefore, for any given three-parameter models $BP(a, b)$ with a preset exponent-pair, the ratio was fixed, irrespective of the data to which the model was fitted. For instance, logistic growth always displays the ratio $m_{infl}/m_{max} = 0.5$ (unless $q = 0$). The four-parameter Richards-model ($a = 1$, b variable) was more flexible. However, for this model the ratio (dependent solely on b) was bounded from below by $1/e$ (ca. 0.368), whereas [37] argued that for vertebrates the true ratio would be significantly smaller, namely around 1/3. (For the generalized Bertalanffy model with $b = 1$ and variable $a < b$, $1/e$ was the upper bound for the ratios.) The higher flexibility was our motive to use the general BP-model, for which any ratio between 0 and 1 could be realized.

The parameters m_{max}, m_{infl}, and t_{infl} are known to have a biological meaning. Asymptotic mass was linked to adult mass [52]. The inflection point was related to a change in diet, from spiders with much keratin (for the growth of feathers) to protein-rich caterpillars (Naef-Daenzer & Keller, 1999). Asymptotic mass was often used to estimate the adult mass. However, we did not use this extrapolation, as after fledging and prior to attaining adult mass there might have occurred another growth phase, which we could not observe for wild birds (Grossmann, 1988.)[53]. Further, the maximal growth rate, i.e. the derivative $m'(t_{infl})$, was suggested as a proxy for the basal metabolic rate [54].

Formatted: Line spacing: Double

By equation (2), for logistic growth the size at the inflection point is always half of the asymptotic size (similar relations hold for the other above-mentioned three-parameter models), while the general model (1) is flexible. (It allows to realize any ratio between 0 and 1.) In this paper we argue that the ratio m_{infl}/m_{max} may have a biological meaning, whence models that are flexible with respect to this ratio would be preferable. To this end we used data from Corsini et al. (2020) on the development of 81 nestlings of blue tits (BT: *Cyanistes caeruleus*) and great tits (GT: *Parus major*) that grew up in a park of the Warsaw city center. For each nestling we identified the best-fit parameters for the BP-model to compute, for each bird, the individual ratio m_{infl}/m_{max}. (This approach differed from the above-mentioned papers that fitted one model to the aggregate data, namely average size-at-age, of many animals.) For further statistical analysis we used environmental data from Szulkin et al. (2020) to explore whether the ratio and other parameters derived from the BP-model could reveal impacts of the environment on avian growth that were unobserved with other models.

The ratio, too, might be biologically relevant, as it was defined from two biologically meaningful parameters. When we compared two BP-growth curves with the same initial value and the same asymptotic limit (example: Figure 1), then the curve with a higher ratio had a higher inflection point, whence (from its t_{infl} on) it was sooner close to the asymptotic limit. Thus, the ratio indicated, how fast the asymptotic size was approached finally. When we compared the ratio with the environmental indicators, we interpreted Figure 1 as follows and sought for supporting evidence: Initially the growth trajectories differed slightly, but at a later phase birds from disadvantaged neighborhoods felt the environmental deterioration (e.g., their parents did not find enough food) and their growth was inhibited, explaining a lower ratio.

Methods

Formatted: Line spacing: Double

Bird and nest-box data

This paper is based on life history data from Corsini et al. (2020)[28-30] and environmental data from Szulkin et al. (2020)[12] that were collected between March and June of 2016. [30] investigated the development of nestlings of blue tits and great tits that grew up at various sites in and around Warsaw, Poland. [12] complemented these growth data by environmental data from the nest-box sites. We received from these authors data about 429 nestlings from 56 nest-boxes at various study sites in and around Warsaw, Poland. . For each bird, data included

the ID of the bird (ring number) and of the nest-box, the species (~~BT~~blue tits or ~~GT~~great tits), the hatching date, the number of hatchlings at the first visit, mass in gram (eight repeated weighing), sex (F or M), and fledging success. To distinguish birds prior to ringing, they were marked at the first measurement.

To ensure comparability, we studied only a fraction of the 429 nestlings. First, to exclude irregular growth patterns from runts, we selected only birds whose sex could be determined (F or M) and that finally became fledged. (We used these criteria as a proxy for healthy birds.) Second, we selected only birds that were weighed at the same eight odd numbered days 1 (hatching), 3, 5, 7, … 15. We thereby considered only nestlings that survived that long~~ (the~~. (The others were for instance, early fledging, runts, or predated~~ by woodpeckers or martens).~~.) As we wished to compare the shapes of the growth curves, we did not consider birds that were weighed at other days, even if they survived. Third, we selected only hatchlings from the first brood. They grew up during May and June~~;~~. (According to open data from Warsaw airport, the month of May was dry with cool nights and June was warm and humid~~ (Figure 1).~~.) This reduced the count of nestlings to 95 birds in 24 nests at two study sites: Kampinos national park (4 nest-boxes with 14 nestlings) and Pole Mokotowskie park close to the city center (20 nest-boxes with 81 nestlings). Fourth, for a statistical comparison of growth in relation to the environment, we removed the 14 Kampinos data. Otherwise, a rural-urban comparison would depend on merely four rural nests. Further, as for our data rural hatching occurred later, it was likely that food availability and composition differed, but there were no data about the diet.

Thus, our study was based on 81 Pole Mokotowskie nestlings. As is evident from a plot of the data (Figure 2), species and sex affected body mass, whence we controlled for these factors: There were 26 female and 31 male blue tits and 12 female and 12 male great tits. ~~To keep sample sizes large enough for statistically significant conclusions we did not consider further stratifications.~~

To study the association between environmental variables and growth we used the following information about the nest-boxes that the above-cited authors had retrieved from geo-information systems or collected at the eight visits. The latter data measured human activity~~,~~ [28], air temperature at the nest~~,~~-box site (average of the morning temperatures in °C at the eight visits), and light and sound pollution~~, the~~ at the nest-box site (in lx and db). The former were ~~distance~~distances to paths and roads~~,~~ (minimal Euclidean distance in m from the nest-box to the center of the next path or road), percentage of impervious surface around the nest (ISA), tree cover in percent around the nest, and NDVI (normalized difference vegetation index). ~~Table 1 informs about the upper and lower observed values (and it adds further explanations about the indices).~~ Methodology details can be found in ~~Corsini et al. (2017), Corsini et al. (2019) and Szulkin et al. (2020).~~[12, 28-30].

~~Data fitting~~

~~Using~~In addition, we considered the association of growth to brood-specific indicators, namely the above-mentioned hatching day, the count of hatchlings at the first visit (4-12 birds/nest at day 1), the count at the last visit (1-9 birds/nest at day 15), and the difference between these counts. This difference may have been caused amongst others by early fledging, by runts (e.g. adverse impact from the environment) or by predation. In the latter case, the likely predators were woodpeckers, as they did not harvest all nestlings at once. (Nests that were destroyed, e.g. by martens, were removed from the sample.)

Calibration

There are different techniques to fit growth curves to data and to analyze them further. A large body of literature used the method of least squares and variants of it. Other approaches used spline-interpolation [55-56], time series methods [57], or stochastic (partial) differential equations [58]. More recently, mixed-effect models became popular [59-61], but to remain

viable they were based on growth curves that could be parameterized by means of elementary functions such as for the Verhulst model [62] or the Richards model [63].

We decided to use a variant of the method of least squares that had worked well for chicken and other dinosaurs [25-26]: We fitted logarithms of BP-growth curves to the logarithmically transformed data. Thereby, given the growth data, we aimed at finding parameters that minimized *SSLE*~~, the sum of squared errors for fitting the logarithmically transformed growth function to logarithmically transformed mass data.~~ of equation (3). If $m(t)$ ~~is~~was a solution of equation (1), using certain exponents $a < b$ and parameters p, q, c, and if (t_i, m_i) ~~are~~were the mass-at-age data (for our data: n ~~=~~ = 8), then *SSLE* ~~is~~was defined by equation (3):

~~$$(3)\quad SSLE = \sum_{i=1}^{n}\left(\ln(m_i) - \ln\left(m(t_i)\right)\right)^2$$~~

~~We used~~ $SSLE = \sum_{i=1}^{n}\left(\ln(m_i) - \ln\left(m(t_i)\right)\right)^2 \quad (3)$

SSLE ~~instead of the more common sum of squared errors for model comparison because this measure was~~was related to a method of weighted least squares using $1/m(t_i)^2$ as weights [64], since its residual, $\ln(m_i)–\ln(m(t_i))$, approximated the relative error, $(m_i–m(t_i))/m(t_i)$.

The logarithmic transformation of the data in equation (3) is an instance of the Box & Cox [65] power transformations that aim at stabilizing variance, making data-fitting less sensitive to higher variances for the higher masses. ~~For~~As can be seen from Figure 2, for our data the spread of the weights of birds of the same species and gender increased with time. Further, shortly before fledging the body mass of birds may reach a peak and then go down or begin to fluctuate ~~(Brown et al., 2007). The suitability of this approach has already been confirmed for chicken (Renner-Martin et al., 2019) and other dinosaurs (Brunner et al., 2019). As was explained there, the implicit statistical assumption for~~ [66], whence we expected also for this reason higher variances for higher masses. [5] suggested to truncate the data at the peak, but we did not truncate our data as this could leave not enough points of time to determine the

Formatted: Line spacing: Double

model-parameters. For our data, the fit remained reasonable also for finally decreasing growth trajectories, as illustrated by Figure 3. (The plotted BP-model curve did not deviate much from the data: maximal error 0.37 g. This curve was best fitting with respect to *SSLE* ~~is a~~ .)

To justify the logarithmic transformation, we checked for each bird, if its data ln(m_i) were normally distributed (lognormal distribution of ~~mass. (For this distribution, variance increases with mass.)~~body mass) and if the residuals in equation (3) were normally distributed (*SSLE* as a maximum likelihood estimation). We used the Anderson-Darling test (see below).

Formatted: Line spacing: Double

Data fitting

For the common three-parameter models there are various software solutions for determining the best-fitting parametric growth curves [67-68]. However, literature reported difficulties with data fitting for the generalized Bertalanffy model [69] and for the Richards model [70]; both models require the optimization of four parameters. For the ~~search of optimal parameters that minimized *SSLE*,~~general five-parameter BP-model (1), standard optimization tools in general failed to find the best-fit parameters, even if they started close to ~~the best-fit parameters (Renner-Martin et al., 2018; for related observations: Loibel et al., 2010; Shi et al., 2014). We~~ them [24]. As for an example of a failure from literature [20], the Levenberg-Marquart algorithm did not converge for the five-parameter BP-model. ([20] reported the following fits for Covid-19 in Shanghai: R-squared for logistic growth was 88.1%, while it was only 86.7% for the general BP-model. This was obviously false, as the BP-model is more general and therefore ~~used a grid-search combined with a custom-made variant of the method of simulated annealing (Vidal, 1993). The details of our approach to optimization were outlined elsewhere (Brunner et al., 2019, Renner-Martin et al., 2019). We did not use simplifications that are common in growth literature.~~it necessarily improves upon logistic growth.)

To simplify data-fitting, several authors suggested to reduce the number of free parameters by not optimizing certain parameters. We did not apply this strategy. For example, for the differential equation (1), instance, many authors defined the parameter $c = m(0)$ by the hatching mass (the first weighing at day 1 (hatching) corresponded to the age t = 0. Many authors defined the parameter c by the hatching mass $m(0) = c$, as then only the four remaining parameters needed to be = 0), as this reduced the number of optimized. parameters by one [41]. In contrast, we used hatching mass as an estimate for the initial value but allowed the optimization to identify a better value of c to find a lower *SSLE*. [52] recommended to identify asymptotic mass with the average adult age of the considered group of birds. We did not use this extrapolation, as after fledging and prior to attaining adult mass there might have occurred another growth phase, as was observed for farmed birds [71]. Thus, adult mass might overestimate the asymptotic mass of the considered growth phase. (We could not observe an additional growth phase for wild birds, as after leaving the nest-boxes they did not return for weighing.)

Formatted: Line spacing: Double

Computations and statistics

We used Mathematica 12.1 of Wolfram Research for computer algebra and statistical computations. Thereby, for data fitting we used the custom-made optimization method described above. The Mathematica code was explained elsewhere (Brunner et al., 2019, Renner-Martin et al., 2019) and it is available as a supporting information for the cited papers.

To resolve these difficulties, for our data we used a grid-search combined with a custom-made variant of the method of simulated annealing [72] for data fitting. Thereby, we defined a grid of exponent-pairs and for each grid-point exponent-pair (a, b) we identified the best-fit growth curve for the three-parameter model $BP(a, b)$ by means of simulated annealing (redesigned to generate positive parameter-values, only). Amongst these growth-curves we then identified the overall best fitting one. We used Mathematica 12 of Wolfram Research for our computations; for details of our approach to optimization and (explanations of) the Mathematica-code we refer to [25-26].

Model comparison

To assess the goodness of fit across different datasets (nestlings), we considered an analogue of the coefficient of determination for *SSLE*, namely RL-squared of equation (4). It assesses the relative improvement of *SSLE* for the best-fit BP-model in comparison to the best-fitting constant model (geometric mean of the masses):

$$RL^2 = 1 - \frac{SSLE\,(\text{BP model})}{SSLE\,(\text{constant})} \quad (4)$$

In view of certain limitations of R-squared [73] that apply also to RL^2, we did not draw further conclusions about model selection, as RL-squared would not be sufficiently selective for such a purpose. However, model selection per se was not an issue of this paper, because for conceptual reasons (flexible ratio m_{infl}/m_{max}) we decided to use the five-parameter BP-model, even if there was a danger of overfitting (meaning that for some data simple models, such as logistic growth, might have been more parsimonious [74]).

Utilizing the information from optimization, we aimed at quantifying the possible consequences of overfitting. For each bird we identified those grid-point exponent-pairs (a, b), where the corresponding three-parameter model, $BP(a, b)$, had a good fit to the data in terms of RL-squared. For each of these models we computed the parameter values of interest. We then evaluated the spread by means of the difference between the 95%-quantile and the 5%-quantile of the parameter-values that were computed for this set of models. (For our data, defining a good fit by $RL^2 \geq 95\%$, in the median about 43,000 models per bird were used.)

Statistical computations

We used the standard statistical tests of the Mathematica software. For all statistical tests we decided at 95% significance (P-value 5%). ~~Thereby, we~~We preferred non-parametric methods suitable for small samples ~~(Hollander & Wolfe 1999). For correlations we computed the~~

Spearman rho and used the Spearman rank test for assessing its significance. We used the [75], such as the one-sided sign test to estimate confidence intervals for the median. When medians, the Mann-Whitney test to check for significant stochastic differences, or the Spearman rank correlation (rho) together with the Spearman rank test. In case we needed distribution assumptions, we used the Anderson & Darling [76] test to verify them.

We identified the best-fit BP-growth curves for each individual bird. This approach avoided the pooling of masses across individuals (fitting growth curves to average-size-at-age). While pooling would be computationally easier (one fit for all females and males of each species) and the variations of the averages might be smaller (resulting in better fits), the aggregate data may miss biologically important differences between individuals (averaging treats them as equal). For the further analysis of the shape-parameters, we controlled for species and gender. This stratification defined four samples (female and male blue tits, female and male great tits) of moderate sample sizes (12-31 individuals). To keep sample sizes large enough for statistically significant conclusions we did not control for nest sizes. (Instead, we used resampling techniques to reduce this influence, see below.)

The search for significant associations between the shape-parameter (ratio) and the 13 (brood-specific and environmental) indicators applied the Spearman rho and the Spearman rank test for each of the four samples. This resulted in 52 tests, whence spurious outcomes were to be expected, where one or more of the tests falsely reported a significant correlation. Thereby, we expected at most five spurious outcomes (P-value 4.45% for six or more false reports of “significant”, assuming a binomial distribution with 52 trials and a chance of 5% for errors). To identify the valid associations, we focused on the highly significant ones. For them, we sought for additional supporting evidence by testing the same associations under two different aspects.

For the first additional aspect we asked: Did variations in the local environments (at or around the nest-sites) lead to different shapes of the growth curves of the nestlings? We tested the hypothesis that nestlings from inferior environments (e.g., higher pollution) were inhibited in their growth. Thereby, we diagnosed a slower growth for these birds if they had stochastically lower ratios than the birds of the other neighborhoods (Mann-Whitney test). As thresholds between inferior and superior environments we used the medians of Table 1 for each indicator and group of birds. Thus, we divided each of the four samples into two groups of approximately equal sizes, namely birds that grew up in an ambience with a high parameter value (equal or larger than the sample median) and the other birds. For these classes we tested for significant differences in the location of the ratio. As for great tits each comparison involved a class with only 3-6 birds, which barely could be representative, we focused on blue tits, only.

For our second additional aspect we explored, if and to what extent the model-parameters did allow to draw inferences about the "social background" of birds: Did birds with distinct growth-patterns (shapes of the growth-curves, ratios) origin from different environments? We defined subsamples of the four groups of birds in terms of the ratio: A bird was high-ratio, if $m_{infl}/m_{max} \geq 0.5$ for the best-fit BP-model growth-curve (the threshold 0.5 comes from logistic growth), and otherwise it was low-ratio. Now, using the Mann-Whitney test we compared the medians of different samples, we used the Mann-Whitney testthe environmental indicators for the subsamples of high-ratio and low-ratio birds. Thus, we compared the indicator values for the nests of 15 high-ratio female blue-tits with the indicator values for 11 low-ratio female blue tits; the same for 19 high-ratio and 12 low-ratio male tits. Again, the so defined samples for great tits were too small (three high-ratio vs. nine low-ratio female great tits, seven high-ratio vs. five low-ratio male great tits), whence we did not consider them.

Formatted: Line spacing: Double

Finally, we explored, if the outcomes were affected by correlations between birds of the same nest. There were potentially negative correlations and positive ones (examples: competition for food, common traits inherited from the parents). Further, assuming that growth was affected by the ability of the parents to supply each nestling with adequate food, then this ability was influenced by nest-size and not only by environmental factors. We used resampling to reduce such dependencies. We selected one bird per nest and repeated the previous analysis of correlations for this small sample (11 female and 11 male blue tits; great tits were not considered). As for these small samples a merely moderate significance could be expected, we gathered evidence by comparing different selection strategies, deterministic (selecting representative birds) or stochastic (bootstrap-sampling).

Results

Formatted: Line spacing: Double

~~Anderson-Darling test was used to test distribution assumptions, specifically the above assumption of lognormally distributed mass.~~

~~Results~~

Formatted: Line spacing: Double

Best-fit ~~model~~ parameters

We first ~~tested~~ observed that for most birds the ~~assumption of log-normal distributions of the masses.~~logarithmic transformation stabilized variance. This ~~assumption is of interest, because under this assumption the~~ confirmed the suitability of *SSLE* ~~parameters would be maximum likelihood estimates and therefore asymptotically~~ . For each bird we tested if its masses (m_i) were log-normally distributed. ~~Hence, statistics derived from *SSLE* (e.g. R-squared, F-ratio test or the Akaike information criterion) could be used to compare models and decide, if simpler models, such as logistic growth, would be preferable over the more general BP-model. For our data, the Anderson-Darling test~~ The Anderson-Darling test did refute this assumption for ~~only~~ six (7.4% of 81) birds~~. Nevertheless, we did not discuss model selection,~~

as (*p*-values below 5%). Further, we had decided to apply the BP model, whereby we perceived tested for each bird, if the fit-residuals for *SSLE* were normally distributed; these residuals were $\ln(m_i)$–$\ln(m(t_i))$ for the eight data (t_i, m_i) and the best-fit BP-model--curve as a smoothening of$m(t)$. Here, the Anderson-Darling test refuted this distribution assumption for five (6.2% of 81) birds. For three birds both distribution assumptions were refuted. We did not discard of these data, as there were only few exceptional birds and for them the fit to the BP-growth curve was still acceptable, as is illustrated by Figure 3.

Next, for each bird we screened the growth data.

curve of the BP-model with optimized parameters to assess its fit to the data. In general, the five-parameter BP-model achieved an excellent fit with median-RL^2 of 99.77%; for 70 of 81 data RL-squared was above 99.5%. However, one bird was exceptional (K7V3278), as the best-fit BP-model did not achieve RL-squared above 95% (namely $RL^2 = 94.2\%$). For this bird, asymptotic mass was excessive. We therefore asked, if overall the data captured both the initial rapid growth and the final flattening of growth. To this end weTable 2 gives an overview of the range of the best-fit model parameters and several statistics computed from them. (Details for each individual bird are provided as a supporting information.) In view of an excessively large asymptotic size for one bird, we first analyzed the quotient of the asymptotic mass over the maximal observed mass (in general, mass at day 15): It ranged from 0.89 to $1.4 \cdot 10^{13}$. for the exceptional bird. However, the median was 1.006 with a narrow confidence interval between . (Lower and upper confidence limits 0.998 and 1.016 (one, both with P-values 2.24% for one-sided sign tests: P-values 2.24%, i.e., adding up to $p = 4.5\%$ or 95.5% confidence). for the interval.)

The focus of this research was on the ratio m_{infl}/m_{max} of the mass at the inflection-point (peak growth) over the asymptotic mass. For five birds there was no inflection point, $a = 0$; for them we set $m_{infl} = 0$ and $t_{infl} = 0$. Mathematically, a higher/lower ratio was related to the shape of the BP-growth curves. This could be visualized by a specific example, the growth trajectories and the inflection points of logistic growth with ratio 0.5 and of an alternative growth curve with a higher ratio 0.63 (Figure 3). Both curves were defined to start at the same initial value

Formatted: Line spacing: Double

(c = 0.1), approach the same asymptotic limit 1, and intersect at the inflection point of the logistic growth curve. Consequently, for the alternative curve the parameters p and q were smaller than the same parameters for logistic growth. Further, the alternative curve had a higher inflection point that was reached later (higher t_{infl}) and that was sooner close to the asymptotic limit. This difference in the growth patterns may be interpreted as a faster/slower growth for a higher/lower ratio m_{infl}/m_{max}. (Note that in this example the final sizes for both growth curves were the same. However, there might be a biological advantage if the final size is approached earlier and the ratio explores this matter.)

The following associations demonstrate that a similar pattern was observed for the bird data: Using the full sample of 81 birds, with high significance a higher ratio m_{infl}/m_{max} was associated a) to a higher mass m_{infl} (Spearman rho 0.47; P-value close to zero for the Spearman rank test), b) to a lower asymptotic mass m_{max} (Spearman rho 0.35, P-value 0.1%), c) to a longer time span t_{infl} till maximal growth (Spearman rho 0.78, P-value close to zero), and d) to lower parameter values p and q (Spearman rho 0.57 and 0.52, respectively, with P-values close to zero). To study this pattern, we defined two classes of birds from their ratio m_{infl}/m_{max}: Using the ratio of logistic growth as a threshold, birds with ratios m_{infl}/m_{max} > 0.5 were high-ratio birds (54% of our sample) and the other low-ratio birds.

Further, for our data a higher ratio was associated to a higher exponent-difference b a (Spearman rho 0.5 with P-value close to zero). This was surprising, as a negative correlation would be expected: In equation (2), if the smaller exponent (a) was kept fixed while the larger one (b) increased, then the ratio decreased. This indicated a peculiarity of the distribution of Figure 4 displays the best-fit exponent-pairs (plot: Figure 4). The exponent-pairs. (Note that another calibration, such as ordinary least squares, would result in different exponent-pairs.) The pairs appeared to be scattered at random, but awhereby the exponent b was much more variable than the exponent a, and we could not observe a concentration near any of the exponent-pairs of the "traditional" three-parameter models (Brody, Bertalanffy, Gompertz, West, Verhulst). A notable observation was the concentration close to the diagonal: Defining a bird as near-diagonal, if its best-fit exponent pair satisfies b a < 1.5 (yellow lines in Figure 4), then 47% of the birds were near-diagonal (the other birds are far-diagonal).

Associations between environmental and model parameters

For this section we systematically searched for significant associations between the indicators of Table 1 and two model parameters for the shape of the growth curves, the ratio m_{infl}/m_{max} and the exponent-difference b a. The stratification of the 81 birds by species and gender defined four samples (BT-F, BT-M, GT-F, and GT-M). In the sequel this resulted in small to moderate sample sizes: 12-31 individuals for the samples used for Table 3 and at least three individuals in each of the subsamples for Table 4 and Table 5. We searched under three different perspectives.smaller than 1.5). For six birds the exponent-pairs were on the line a = 0 (no inflection point).

Formatted: Line spacing: Double

~~First, we~~ We stratified the birds by species and by gender and marked their exponent-pairs by different colors in Figure 4. The medians of the exponent-pairs were (0.495, 2.28), (0.63, 2.31), (0.815, 3.155), and (0.835, 3.69) for female and male blue tits, and female and male great tits, respectively. Again, there was no apparent pattern. The proportion of near-diagonal birds was almost equal amongst all groups of birds: 50%, 48%, 42%, and 42% for female and male blue tits, and female and male great tits, respectively.

Using equation (2), we computed the best-fit ratios, m_{infl}/m_{max}, from the best-fit exponents. For our data, the best-fit ratios were large, when compared to the claim [37] that for vertebrates the ratios would be around 1/3. The ratios ranged from 0 ($a = 0$) to 0.835, whereby 0.1 was the least ratio for a sigmoidal bird (meaning one with a sigmoidal best-fit growth curve: $a > 0$). For 21 birds (19 of them sigmoidal) the ratio was smaller than $1/e \approx 0.368$ (the lower bound for the ratios of Richards model and the upper bound for the ratios of the generalized Bertalanffy model) and for 48 birds it was above 0.5 (ratio of logistic growth).

For all four groups of birds, the ratios were significantly greater than Bertalanffy's claim of 1/3 (one-sided sign test for sigmoidal birds: P-value below 1.5% for each group). The median ratios (and 95% confidence bounds) were 0.511 (0.467–0.59) for female blue tits, 0.503 (0.467–0.57) for male blue tits, 0.459 (0.42–0.59) for female great tits, and 0.528 (0.42–0.65) for male great tits. (The confidence limits were computed for the sigmoidal birds using one-sided sign-tests, whose P-values added up to a value between 3.9% and 5.2%.)

Identification of the ratio from the shape

We asked, how closely the ratio, a shape-parameter, was related to the shape: For given growth-data, could the ratio be reconstructed from the shape of their growth trajectory, and if so, how accurate? Note, that we deliberately did not define the term "shape". (Literature used the term shape-parameter without defining "shape" [77]). Rather, we assumed that growth

curves of the BP-family with a good fit to the data ought to have about the same shape, and we expected that the ratios of curves with similar shapes should have about the same values.

To study the variability of the ratio, we first identified the for each bird the exponent-pairs that defined BP-models with “good” fits, meaning e.g. $RL^2 \geq 95\%$. The green area in Figure 5 plots the exponent-pairs from the initial search grid (yellow), whose three-parameter models $BP(a, b)$ achieved $RL^2 \geq 95\%$ for 80 of 81 data. (For one bird, only a lower RL^2 could be achieved.) Except for Brody, all traditional models achieved this fit, whereby logistic growth was outstanding with RL-squared above 98% for 78 data (red area).

Figure 5 cautions that we could expect only a fuzzy correspondence between the shape and the shape-parameters. For, unless we require an extremely good fit, the variability of the ratio might be high and our expectation about its identification from the inspection of the shape of the growth trajectory might be overly optimistic. For, given any of the 80 birds that allowed for a good fit ($RL^2 \geq 95\%$), there was a large area of exponent-pairs (the green area and beyond, depending on the bird) that represented alternative models, whose growth curves had a good fit to the growth-data and that therefore had about the same shape. Given any bird and its best-fit BP-model growth-curve, even a significant change in two parameters (the exponent-pair) could almost be offset by changes in the other three parameters without changing the good fit (and thus the shape) of the growth curve significantly.

However, the ratio turned out to be somewhat resilient against this variability. Considering models with $RL^2 \geq 95\%$, the spread of the ratio was at least 0.495, but for models with $RL^2 \geq 99.5\%$ the minimal spread was reduced to 0.06. Figure 5 explains this: Requiring a higher RL-squared made the area of the exponent-pairs with a good fit smaller (compare the red and green areas), but not in the same proportion in all directions: It became narrower in the direction of the diagonal, which meant a smaller variability of the ratio (c.f. the plotted contour lines of the ratio).

Moreover, the best-fit ratio could be estimated by the median of the ratios of the growth-curves of the models with a “very good” fit: There were 66 birds with an inflection point, whose best-fit model satisfied $RL^2 \geq 99.5\%$. For 47 (=71% of 66) birds the relative error between the best-fit ratio and the median of the ratios for reasonably fitting growth curves (meaning $RL^2 \geq 99.5\%$) was below 10%, and for 47 birds the absolute difference between the best-fit ratio and the median was below 0.05.

Correlations with the ratio

In view of the difficulties with parameter reconstruction, we explored the possible biological meaning of the ratio indirectly: We interpreted the ratio as random variable that was defined for mass-at-age data. (Thus, the ratio was defined as a function on the set of time series. We therefore considered also non-sigmoidal trajectories, setting their ratios zero.) We sought for statistically significant associations between this random variable and factors that literature considered as relevant for growth. Hence, we searched systematically for significant associations between the ratio, four brood-specific indicators (hatching day, count of hatchlings at the first visit, count at the last visit, and difference between these counts), and nine environmental indicators (human activity, ISA, light pollution, NDVI, distance to a path, distance to a road, sound pollution, morning temperature, and tree cover). We studied four groups of birds independently: female/male blue/great tits.

First, we checked for correlations between the ~~indicators and the two shape parameters). With respect to the ratio (left half of Table 3), most of the nine~~ ratio, m_{infl}/m_{max}, and the brood-specific and environmental indicators. We found eleven significant correlations ~~were highly significant~~ (P-value below ~~1~~5%, using the Spearman rank test) and ~~strong~~we expected that at most five of them were spurious (see the methods). For the brood-specific indicators we found five highly significant correlations with the ratio. Initial nest-sizes were negatively correlated with the ratio for female blue tits, female great tits, and male great tits (Spearman ~~rho with~~

absolute value 0.5 or higher). As a common observation for all groups, except male blue tits, a larger initial count of hatchlings was associated to a lower ratio m_{infl}/m_{max} (three correlations). Further, both for female and for malerank correlation coefficients and P-values $\rho = -0.517$ with $p = 0.68\%$, $\rho = -0.86$ with $p = 0.03\%$, and $\rho = -0.781$ with $p = 0.27\%$, respectively). The count of lost siblings (nest-size difference) was negatively correlated with the ratio for female and male blue tits ($\rho = -0.555$ with $p = 0.32\%$ and $\rho = -0.814$ with $p = 0.13\%$, respectively). For the environmental indicators, we found six significant correlations, the following four highly significant: For blue tits, a higher percentage of impervious area around the nest-box (ISA) and a higher level of light pollution was associated to a lower ratio m_{infl}/m_{max} (four correlations). There were eightnegatively correlated to a lower ratio. (For ISA, $\rho = -0.54$ with $p = 0.4\%$ for females and $\rho = -0.48$ with $p = 0.69\%$ for males, and for light pollution $\rho = -0.51$ with $p = 0.35\%$ for males and $\rho = -0.64$ with $p = 0.04\%$ for females.) Two associations were of a lower significance, namely a positive correlation with NDVI (more is better) for male great tits ($\rho = 0.701$ with $p = 1.1\%$) and a negative one with sound pollution for female blue tits ($\rho = -0.43$ with $p = 2.8\%$). The other comparisons were not significant.

The highly significant correlations between environmental indicators and the exponent-difference $b-a$ (right half of Table 3), in particular the following: For female and male blue tits, a higher of the ratio with initial nest size for three groups of birds gave rise to the question if the four highly significant associations of the ratios of blue tits with environmental indicators were insofar artificial, as birds in larger nests might have dominated the computations. We therefore considered resampling, defining subsamples with one bird for each nest. In view of the smaller sample sizes (11 male and female birds, each), we did not expect low P-values.

First, we selected the birds deterministically. If for each nest we selected the bird (of the required gender) with “typical growth” (ratio nearest to the nest-median), then for both groups

of female and male blue tits the ratio had a significant negative correlation with light pollution and ISA (P-values below 3%). If we grouped together the fastest-growing birds from each nest (highest ratio), then for females we obtained a significant negative correlation of light pollution with ratio. If we grouped together the birds with the lowest ratio of their nests, then both for females and for males we obtained significant correlations between ratio and ISA and for females also between ratio and light pollution.

Next, we used bootstrap simulations: Given the groups of female and male blue tits, for each group we selected one bird per nest at random and for this sample we tested the associations between the environmental indicators and the ratio. We recorded the Spearman rho together with its P-value and repeated the simulation thousand times. Both for female and male blue tits, and for the tests for a significant correlation between the ratio and ISA or light pollution, Spearman rho was negative for all simulations. Further, for all groups and indicators, with one exception the median of the thousand P-values was below 5%. The exception was the association between ratio and ISA for female blue tits (median P-value 6.3%). The strongest association with the ratio was observed for females with respect to light pollution: $\rho < -0.5$ and $p < 12\%$ for thousand simulations.

Thus, resampling confirmed the significant correlations between growth and the indicators impervious area (ISA) and light pollution. It thereby suggested a stronger evidence for a potential impact from light pollution on growth and a weaker one from ISA.

Further associations with the ratio for blue tits

To add supporting evidence for the potential impact of light pollution and impervious area on the growth of blue tits, and check for possible additional associations, we added tests for different aspects of the associations.

Was the ratio stochastically lower (Mann-Whitney test) in highly polluted environments, than in lowly polluted ones? We asked this for all 13 indicators for the two groups of female and male blue tits. We found five significant associations, at most four of them spurious. (This was based on a P-value 3.3% for a binomial distribution assuming 26 comparisons and a 5% chance of a false significance.) Nestlings of blue tits that grew up in environments with high percentage of impervious area (ISA) or a high level of light pollution was associated to a lower exponent-difference, and for female and male great tits a higher distance to a path was related to a lower exponent-difference. The maximal number of significant correlations in Table 3 was observed for the sample GT-F with respect to the ratio (4, compared to 1-2 for the other samples).had stochastically lower ratios, whereby one test was highly significant. (In detail, for birds from nests with high and low ISA, the median ratios were 0.473 and 0.595, respectively, for males and 0.467 and 0.529, respectively, for females, with $p = 0.316\%$ for males and $p = 3.49\%$ for females. For high and low light pollution the median ratios were $0.493 < 0.576$ for females and $0.491 < 0.599$ for males with $p = 4.15\%$ for females and $p = 1.57\%$ for males.) Further, male blue tits from nests with a higher NDVI (better environment) had a significantly higher ratio.

Formatted: Line spacing: Double

Formatted: Font color: Auto

Second, we complemented the study of individual correlations by a comparison at the population level. We compared the overall environmental situation between two complementary groups of birds that were defined from the shape parameters (high-ratio vs. other birds, and near-diagonal vs. other birds, respectively). Thus, for each indicator we computed its median for each of the two complementary groups of birds, and we tested, if the two medians differed significantly (Mann-Whitney test), resulting in 24 significant outcomes. Thereby, several of the 17 significant correlations of Table 3 could be explained by related differences in the environments of the nest sites that were chosen by (the parents of) these birds (Table 4): The negative correlation between nest size and the ratio m_{infl}/m_{max} was explained by the observation that for all samples (except BT-M) in the median the high-ratio birds had fewer siblings than the other birds. For blue tits, the negative correlation of the ratio with the percentage of impervious area was confirmed by the lower median of this percentage for high-ratio birds. Further, Table 4 identified significant differences in the environmental conditions, specifically for the comparison of near-diagonal and other birds, that were not detected from the correlations. The maximal number of significant associations in Table 4 was observed for the sample GT-F with respect to the exponent-difference (8, compared to 0-4 for the other samples).

Third, we tested the hypothesis that the shape of the growth curves might have been affected by favorable (or unfavorable) environments. To this end, for each indicator we divided our samples (i.e. BT-F, BT-M, GT-F, GT-M) into two groups of approximately equal sizes, namely birds that grew up in an ambience with a high parameter value (larger than the sample median) and the other birds. We then computed the medians of the shape parameters and used the Mann-Whitney test to check for stochastic differences. This resulted in 20 significant differences (Table 5). For example, for the two GT samples the ratio m_{infl}/m_{max} was smaller for birds that had more siblings than the median than for the other ones. (The same was observed for BT-F However, its division was unbalanced.) Further, as with the negative Spearman rho of Table 3, a higher percentage of impervious area and higher light pollution was associated to a significantly smaller median ratio for the blue tits from such neighborhoods. For light pollution, this observation was confirmed by the exponent-difference (which is correlated to the ratio). The maximal number of significant associations in Table 5 was observed for the sample GT-M with respect to the exponent-difference (6, compared to 1-4 for the other samples).

Was the level of environmental pollution stochastically lower near the nests of high-ratio birds than near those of low-ratio birds? We identified seven significant associations, at most four of them spurious (see above). High-ratio blue tits likely grew up in environments with less light pollution and a lower ISA. (Specifically, the medians of ISA were 4.54% < 13.33% with p = 1.18% for high-ratio and low-ratio females, and 3.58% < 8.88% with p = 1.14% for high-ratio and low-ratio males. For light pollution we observed the medians 2852 lx < 3160 lx with p = 2.0% for males and 2896 lx <5937 lx with p = 4.1% for females.) Further, high-ratio females originated likely from smaller nests (median initial sizes 9 and 10.5, p = 1.2%) with less sound pollution (medians 64 db < 68 db, p = 0.87%) and fewer siblings lost (median nest-size differences 3 and 6 with p =0.43%).

Related issue

The various brood-specific and environmental indicators were not independent. In particular, nest-size difference (between the initial and final counts) was associated to several environmental indicators.

Testing for 12 indicators, we observed 18 significant correlations, of them expecting at most six spurious ones (P-value 3.2% for six or more false tests amongst 48 ones, each with a 5%

chance of errors). Blue tits displayed highly significant negative correlations (P-values close to zero) of nest-size difference with the final count and significant positive correlations with the initial count and the hatching day. With respect to the environmental indicators, for blue tits there were highly significant positive correlations of nest-size difference with ISA ($\rho = 0.565$ with $p = 0.26\%$ for females, $\rho = 0.412$ with $p = 2.1\%$ for males) and with light pollution (females only: $\rho = 0.548$ with $p = 0.37\%$). However, for great tits, the correlations with ISA were negative, significantly for females ($\rho = -0.676$ with $p = 1.6\%$). Further, nest-size difference was positively correlated for blue tits to sound pollution ($\rho = 0.854$ for females and $\rho = 0.826$ for males with both P-values close to zero), temperature ($\rho = 0.681$ with $p = 0.012\%$ for females, $\rho = 0.369$ with $p = 4.1\%$ for males), it was negatively correlated for male blue tits to human activity ($\rho = -0.386$ with $p = 3.2\%$) and NDVI ($\rho = -0.529$ with $p = 0.22\%$), and it was positive correlated for females to the distance to a path ($\rho = -0.445$ with $p = 2.3\%$ for blue tits, $\rho = 0.676$ with $p = 1.6\%$ for great tits).

Discussion and conclusions

Formatted: Line spacing: Double

We studied environmental indicators from avian literature that potentially affect the growth of birds. ~~However, previously~~Previously such findings were based on comparisons of large samples from clearly distinct environments, ~~e.g.~~such as the urban and rural dichotomy~~.~~ [8, 17]. In our context, the sample size was comparably small, and we expected that the environmental variation across the urban study site (Pole Mokotowskie park) would be gradual. Could we nevertheless identify ~~significantly different growth patterns?~~significant differences in the growth parameters? To find any potential environmental impact, we tested 13 indicators, but this immediately gave rise to the concern of spurious outcomes amongst 52 tests for four groups of birds. We responded to this risk by a three-step testing-strategy. First, we used three different tests for the same associations that utilized different aspects, namely a)

correlation tests, b) location tests for the shape-parameter (ratio) of birds from clearly distinct environments, and c) location tests of the environmental parameters for birds with clearly distinct shapes of the growth curves (high ratio vs. low ratio birds). If the same association was supported by three different tests, then it was less likely that it was spurious. Second, we only considered associations that were highly significant for at least one of the three tests, as such associations a priori were less likely to be spurious. And third, extending the idea of multiple support, we focused on associations that were significant for the females and the males of the same species or that were even shared by different species. For such associations we speculated with a biological reason, although it was not the purpose of the paper to detect any causations.

For instance, it is plausible that the efforts of parents to feed their nestlings may not grow proportional to the number of nestlings, whence more siblings to feed may reduce the efforts per nestling and affect the shape of the nestlings' growth curves. Considering the small sample sizes, were the changes distinct enough so that statistical tests could detect them? Thus, it was not clear a priori, whether these changes would occur for all nests in about the same way. Further, why should the two considered shape parameters (ratio m_{infl}/m_{max} and exponent difference $b-a$) be sensitive to such changes? As was noted for Figure 4, a higher/lower ratio could be interpreted as faster/slower growth and the exponent-difference was correlated to the ratio, but there are many other shape parameters conceivable that could as well measure faster/slower growth. For another example, it is plausible that impervious area around the nest may inhibit the success of fetching food and affect the growth of the nestlings. However, why should this matter within the narrow confines of an urban park?

To overcome these difficulties, we increased the number of tests. In total, for each of the 11 environmental indicators we have applied six tests to each sample, resulting in 264 tests for significance (the entries in Tables 3 to 5). Amongst them, at most 19 outcomes would be spurious, recording a false significance (P-value 4.4% for a binomial distribution assuming a 5% chance for a false "significant"), while for our samples 61 outcomes were significant. Further, several of these outcomes were highly significant, or they were significant under two or three aspects (i.e. they occurred in 2-3 tables), or they were observed for two or more samples. For such outcomes there may have been indeed an association between the environmental indicator and the considered shape parameter. Thereby, we obtained the maximal number of significant associations for light pollution (11 of 24 tests were significant), followed by the impervious area (10 significant tests) and the count of hatchlings (9 significant tests); for the other indicators, between seven and none (temperature) of the tests were significant.

We conclude that despite the small sample size the BP-model could detect significant statistical association between some local environmental variables and certain shape

~~parameters of the BP-model, namely the ratio m_{infl}/m_{max} and the exponent difference $b-a$. Thereby the exponent difference was meaningful only for the BP-model, while the ratio would be meaningful for all other models. However, as noted in the introduction, for many common three-parameter models of avian literature the ratio is fixed (e.g. ratio 0.5 for logistic growth). We conclude that these more traditional three-parameter models are not suitable to explore and compare the shapes of growth curves. We recommend the BP-model for such a purpose.~~

~~Acknowledgements and declarations~~

For blue tits, we first concluded that most likely there were valid significant statistical associations between some environmental variables and the ratio, m_{infl}/m_{max}. For, the count of the significant associations exceeded the likely count of spurious outcomes in each testing-round (outlined in the results-section) and several associations were highly significant. Thereby, we identified two environmental indicators as outstanding: impervious area (ISA) and light pollution. For all three testing approaches, a higher level of urbanization (impervious area, light pollution) was associated to a lower ratio, which in turn indicated a finally slower growth towards the adult (asymptotic) mass. These findings remained stable, when we eliminated the possible dependency on nest-size by resampling with one bird from each nest.

Avian literature confirms that these findings were biologically plausible. For instance, impervious area around the nest could inhibit the success of fetching food [11] and this clearly would affect the growth of the nestlings. Further, it has long been known that light pollution affects birds [13]. But now we wondered: Why did only these two environmental differences matter and why did we not observe similar associations with indicators that are known to have strong impacts? We conjecture that environmental factors may have masked certain associations and enhanced, others. Sound pollution might be an example. It is known to affect certain passerine species adversely, as for instance in noisy environments female sparrows reduced the provisioning rate for their brood [16]. For our data, sound pollution had a highly significant positive correlation with nest-size difference, but the evidence for an impact of sound pollution on the growth of the nestlings was weak. (For female blue tits, only, we observed a significant negative correlation of sound pollution with the ratio and high-ratio

birds were likely to have come from less noisy nests.) We speculate that sound pollution might have affected breeding success adversely (higher nest-size difference) and this in turn might have mitigated the impact on growth. (Assuming, that a shrinking nest-size might increase the per capita food supply for the nestlings, the reduction of nest-size could have saved the surviving birds from starvation.)

As to a more technical issue, we observed that for about half of the birds the best-fit exponent-pair was close to the diagonal. Was there a biological reason for this? This question was the starting point of the present paper, but in view of the high variability (see Figure 5) of the exponent-difference (b–a) we did not arrive at conclusive answers. (For blue tits, the potential impact of light pollution was supported again: significant negative correlation with the exponent-difference.) We therefore sought for a function on the exponent-pairs with a lower variability; this was the ratio.

We conclude, irrespective of the biological interpretations of our results, that we could verify that computing the ratio by means of the BP-model was a suitable instrument to detect small variations in avian growth due to slight differences in the environment. As the ratio is meaningful for any model, researchers interested in studying the relation of the ratio to environmental indicators are not confined to the BP-model. Yet, as noted in the introduction and pointed out also by [78], the used model must be flexible with respect to the ratio, whence the more traditional models (logistic growth) are not suitable for this task, even if their fit might be good (as for the logistic model for most of the present data). It is of interest, if other classes of models that are flexible with respect to the ratio provide similar outcomes.

Learning from the limitations of the present study, sample size was an issue in several aspects. First, to allow for more refined stratifications much larger samples of birds would be desirable. For great tits, the above described second and third round of tests required further stratifications, but then the sample sizes became too small to draw meaningful conclusions

and consequently we could not identify differences between the species in their responses to environmental challenges. Owing to the interventions by predators, one would require a huge sample at the beginning (for the present research, the data of 81 birds were used, but five times as many nestlings were observed, initially) and lots of computing power (with commercial computers, the data fit lasted for up to a week per bird). Second, part of the high variability of the size-parameters was caused by the relatively small number of time-points used. Weighing the birds daily rather than at every second day would have reduced the size of the green region in Figure 5.

Acknowledgements

This paper originated from conversations with Michela Corsini, and Eva Maria Schöll and Irene Di Lecce, who provided and explained to us the raw datamethodology of the papers [12, 28-30]. The data for these papers were collected under projects directed by Marta Szulkin as the principal investigator. She gave us the permission to publish the data as a supporting information (part of S1File) for their paper Corsini et al. (2020). The the present paper. As this research was ethically approved, there occurred no ethical issues for this paper.

The authors are grateful to MichaelaMichela Corsini and Júlio Neto, who provided insightful comments on preliminary versions of our paper. Further, the authors appreciate the helpful remarksextensive comments by the editorsSteve Oswald and Tales J. Fernandes, the two reviewers of this journal. They helped to improve the paper substantially.

The authors declare no competing interests and there occurred no ethical issues, as the paper was based on literature data from ethically approved research.

This source also links to the repository for these data. The Mathematica code for optimization is stored in repositories for Brunner et al. (2019) and Renner-Martin et al. (2018, 2019). In addition, the authors have prepared supporting information. [Links, file names and descriptions will be added upon publication.]

Supporting Information

S1File.xlsx: Excel file with explanations of the columns in rows 1-2. Columns A-D provide the bird IDs together with general information, E-Q provide the brood-specific and environmental data for each nestling (and nest), R-Y list the bird-mass at days 1 to 15 (odd days, only), and AA-AH provide for each bird the best-fit parameters of its five-parameter BP-model together with additional information about the model.

References

[1] Aggrey SRicklefs R.E. (2002) ComparisonA graphical method of three nonlinear and spline regression models for describing chickenfitting equations to growth curves. Poultry Science 81:1782-1788Ecology 1967, 48: 978-983.

Formatted: Line spacing: single

Formatted: Heading 2

Formatted: List Paragraph, Indent: Left: 0", Numbered + Level: 1 + Numbering Style: 1, 2, 3, … + Start at: 1 + Alignment: Left + Aligned at: 0.25" + Indent at: 0.5"

Bańbura J., Bańbura M. (2012) Blue tits Cyanistes caeruleus and great tits Parus major as urban habitat breeders. Inter Studies Sparrows Verhulst P.F. Notice sur la loi que la population suit dans son accroissement. Curr. Math. Phys. 36: 66-72.

[2] BertalanffyL.v. (1949) 1838, 10:113-121.
[3] Pearl R., Reed L.J. On the rate of growth of the population of the United States since 1790 and its mathematical representation. Proceedings of the National Academy of Sciences USA 1920, 6: 275-288.

Ricklefs R.E.) The Optimization of Growth RateProblems of organic growth. Nature 163: 156-158.

[4] BertalanffyL.v. (1957) Quantitative laws in metabolism and Altricial Birds. Ecology 1984, 65: 1602-1616.
[5] Remeš V., Martin T.E. Environmental influences on the evolution of growth. Quarterly Revues and developmental rates in Biology 32: 217-231passerines. Evolution 2002, 56: 2505–2518.
[6] Bleu J, Agostini S, Biard C (2017) Nest-box temperature affects clutch size, incubation initiation, and nestling health in great tits. Behavioral Ecology 2017, 28: 793-802.

Brody S. (1945) Bioenergetics and Growth. Hafner Publ. Comp., New York, NY, USA.

[1] Brown W.P., Eggermont P., LaRiccia V., Roth R.R. (2007) Are parametric models suitable for estimating avian growth rates? Journal of Avian Biology 38: 495-506.

Brunner N., Kühleitner M., Nowak W.G., Renner-Martin K., Scheicher K. (2019) Comparing growth patterns of three species: Similarities and differences. PLoS ONE 14, published online: DOI 10.1371/journal.pone.0224168.

Calder WA III (1985) Size, Function, and Life History. Harvard Univ. Press, Cambridge, MA, USA.

[1] Corsini M., Dubiec A., Marrot P., Szulkin M. (2017) Humans and Tits in the City: Quantifying the Effects of Human Presence on Great Tit and Blue Tit Reproductive Trait inVariation. Frontiers in Ecology and Evolution 5, published online: DOI 10.3389/fevo.2017.00082.

Corsini M., Marrot P., Szulkin M. (2019) Quantifying human presence in a heterogeneous urban landscape. Behavioral Ecology30: 1632-1641.

Corsini M., Schöll E.M., Di Lecce I., Chatelain, M., Dubiec A., Szulkin M. (2020) Growing in the city: urban evolutionary ecology of avian growth rates. Evolutionary Applications, published online: DOI 10.1111/eva.13081.

Da Silva A., Samplonius J.M., Schlicht E., Valcu M., Kempenaers B. (2014) Artificial night lighting rather than traffic noise affects the daily timing of dawn and dusk singing in common European songbirds. Behavioural Ecology 25: 1037-1047.

Francis C.D. (2015) Vocal traits and diet explain avian sensitivities to anthropogenic noise. Global Change Biology 21: 1809-1820.

Garcia O. (1983) A stochastic differential equation model for the height growth of forest stands. Biometrics 39: 1059-1072.

Gladalski M., Banbura M., Kalinski A., Markowski M., Skwarska J., Wawrzyniak J., Zielinski P., Cyzewska I., Mankowska D., Banbura J.(2016) Effects of human-related disturbance on breedingsuccess of urban and non-urban blue tits (Cyanistes caeruleus). Urban Ecosystems 19, published online: DOI 10.1007/s11252-016-0543-3.

Gompertz B. (1832) On the nature of the function expressive of the law of human mortality, and on a new mode of determining the value of life contingencies. Philos. Trans. R. Soc. London 123: 513-585.

Grossman M. (1988) Multiphasic analysis of growth curves in chicken. Poultry Science 67: 33-42.

Halfwerk W., Holleman L.J., Lessells C.K., Slabbekoorn H. (2011) Negative impact of traffic noise on avian reproductive success. Journal of Applied Ecology 48: 210-219.

Hollander M.A., Wolfe D.E. (1999) Nonparametric Statistical Methods, Wiley, Chichester, UK.

Hsing Y. (2005) Economic growth and income inequality: the case of the US. International Journal of Social Economics 32: 639-647.

Jacobs J., Rockne R.C., Hawkins-Daarud A.J., Jackson P.R., Johnston S.K., Kinahan P., Swanson K.R. (2019) Improved model prediction of glioma growthutilizing tissue-specific boundary effects. Mathematical Biosciences 312: 59-66.

Katsanevakis S., Maravelias C.D. (2008) Modelling fish growth: multi-model inferenceas a better alternative to a priori using von Bertalanffy equation. Fish and Fisheries9:178-187.

Formatted: List Paragraph, Indent: Left: 0", Numbered + Level: 1 + Numbering Style: 1, 2, 3, … + Start at: 1 + Alignment: Left + Aligned at: 0.25" + Indent at: 0.5"

Formatted: List Paragraph, Indent: Left: 0", Numbered + Level: 1 + Numbering Style: 1, 2, 3, … + Start at: 1 + Alignment: Left + Aligned at: 0.25" + Indent at: 0.5"

Formatted: List Paragraph, Indent: Left: 0", Numbered + Level: 1 + Numbering Style: 1, 2, 3, … + Start at: 1 + Alignment: Left + Aligned at: 0.25" + Indent at: 0.5"

Kirschel A.N., Blumstein D.T., Cohen R.E., Buermann W., Smith T.B., Slabbekoorn H. (2009) Birdsong tuned to the environment: green hylia song varies with elevation, tree cover, and noise. Behavioral Ecology 20: 1089-1095.

Kuhi H.D., Porter T., López S., Kebreab E., Strathe A., Dumas A., France J. (2010) A review of mathematical functions for the analysis of growth in poultry. Worlds Poultry Science Journal 66: 227-240.

Loibel, S., Andrade M.G., do Val J.B.R., de Freitas A.R. (2010) Richards growth model and viability indicators for populations subject to interventions. Anais da Academia Brasileira de Ciências 82, published online: DOI 10.1590/S0001-37652010000400028.

Maceina M.J. (2007) Use of piecewise nonlinear models to estimate variable size-relatedmortality rates. North American Journal of Fisheries Management 27:971-977.

Manabe A., Yamakawa T., Ohnishi S., Akamine T., Narimatsu Y., Tanaka H., Funamoto T., Ueda Y., Yamamoto T. (2018) A novel growth function incorporating the effects of reproductive energy allocation. PLoS ONE 13, published online: DOI 10.1371/journal.pone.0199346

Marusic M., Bajzer Z. (1993) Generalized Two-Parameter Equations of Growth. Journal of Mathematical Analysis and Applications 179: 446-462.

Naef-Daenzer B., Keller L.K. (1999) The foraging performance of great and blue tits (Parus major and P. caeruleus) in relation to Caterpillar development, and its consequences for nestling growth and fledging weight. Journal of Animal Ecology 68: 708-718.

[7] Ndithia H., Bakari S., Matson K., Muchai M., Tieleman I. (2017) Geographical and temporal variation in environmental conditions affects nestling growth but not immune function in a year-round breeding equatorial lark. Frontiers in Zoology 2017, 14, published online: DOI 10.1186/s12983-017-0213-1.

Ohnishi S., Yamakawa T., Akamine T. (2014) On the Analytical Solution for the Pütter-Bertalanffy Growth Equation. Journal of Theoretical Biology 343: 174-177.

Pauly D., Cheung W.W.L. (2018) Sound physiological knowledge and principles in modeling shrinking of fishes under climate change. Global Change Biology 24, published online: DOI 10.1111/gcb.13831.

Pinheiro J.C., Bates D.M. (2000) Mixed-Effects Models in S and S-Plus. Springer Publishing, New York, USA.

Pütter A. (1920) Studien über physiologische Ähnlichkeit. VI. Wachstumsähnlichkeiten. Pflügers Archiv für die Gesamte Physiologie des Menschen und der Tiere 180: 298-340.

Ricklefs R.E. (1967) A graphical method of fitting equations to growth curves. Ecology 48: 978-983.

Ricklefs R.E. (1984) The Optimization of Growth Rate in Altricial Birds. Ecology 65: 1602-1616.

[8] Remacha C., Delgado J.A., Bulaic M., Pérez-Tris J. (2016) Human disturbance during early life impairs nestling growth in birds inhabiting a nature recreation area. PLoS ONE 2016, 11, published online: DOI 10.1371/journal.pone.0166748.

[9] Remeš V. (2007) Avian growth and development rates and age- specific mortality: the roles of nest predation and adult mortality. Evolutionary Biology 2007, 20: 320-325.

[1] Kirschel A.N., Blumstein D.T., Cohen R.E., Buermann W., Smith T.B., Slabbekoorn H. Birdsong tuned to the environment: green hylia song varies with elevation, tree cover, and noise. Behavioral Ecology Renner-Martin K., Brunner N., Kühleitner M., Nowak W.G., Scheicher K. (2018) On the Exponent in the Von Bertalanffy Growth Model. PeerJ 6, published online:pmid 29312827.

Renner-Martin K., Brunner N., Kühleitner M., Nowak W.G., Scheicher K. (2019) Best-fitting Growth Curves of the Von Bertalanffy-Pütter Type. Poultry Science 98: 3587-3592.

Shi P.J., Ishikawa T., Sandhu H.S., Hui C., Chakraborty A., Jin X.S., Tachihara K., Li B.L. (2014). On the 3/4-exponent van Bertalanffy equation for ontogenetic growth. Ecological Modelling 276:23-28.

[1] Serroyen J., Molenberghs G., Verbek G., Davidian M. (2009) Non-linear Models for Longitudinal Data. American Statistician 63: 378-388.

Stewart J., Robbins W.D., Rowling K., Hegarty A., Gould A. (2013) A multifaceted approach to modelling growth of the Australian bonito, Sarda australis (Family Scombridae), with some observations on its reproductive biology. Marine and Freshwater Research 64: 671-678.

[10] 2009, 20: 1089-1095.

Formatted: List Paragraph, Indent: Left: 0", Numbered + Level: 1 + Numbering Style: 1, 2, 3, … + Start at: 1 + Alignment: Left + Aligned at: 0.25" + Indent at: 0.5"

Formatted: List Paragraph, Indent: Left: 0", Numbered + Level: 1 + Numbering Style: 1, 2, 3, … + Start at: 1 + Alignment: Left + Aligned at: 0.25" + Indent at: 0.5"

Formatted: List Paragraph, Indent: Left: 0", Numbered + Level: 1 + Numbering Style: 1, 2, 3, … + Start at: 1 + Alignment: Left + Aligned at: 0.25" + Indent at: 0.5"

[11] Juárez R., Chacón‐ Madrigal E., Sandoval L. Urbanization has opposite effects on the territory size of two passerine birds. Avian Research 2020, published online: DOI 10.1186/s40657-020-00198-6.

Strathe A.B., Danfær A., Sørensen H., Kebreab E. (2010) A multilevel nonlinear mixed effects approach to model growth in pigs. Journal of Animal Science 88:638-649.

[12] Szulkin M., Garroway C.J., Corsini M., Kotarba A.Z., Dominoni D. (2020) How to quantify urbanization when testing for urban evolution? In: Szulkin M., Munshi-South J., Charmantier A. (Eds.), Urban Evolutionary Biology. Oxford University Press 2020, in print.

[13] Da Silva A., Samplonius J.M., Schlicht E., Valcu M., Kempenaers B. Artificial night lighting rather than traffic noise affects the daily timing of dawn and dusk singing in common European songbirds. Behavioural Ecology 2014, 25: 1037-1047.

[14] Francis C.D. Vocal traits and diet explain avian sensitivities to anthropogenic noise. Global Change Biology 2015, 21: 1809-1820.

[15] Halfwerk W., Holleman L.J., Lessells C.K., Slabbekoorn H. Negative impact of traffic noise on avian reproductive success. Journal of Applied Ecology 2011, 48: 210-219.

[16] Schroeder J., Nakagawa S., Cleasby I.R., Burke T. Passerine birds breeding under chronic noise experience reduced fitness. PloS ONE 2012, 7, published online: DOI 10.1371/journal.pone.0039200.

[17] Gladalski M., Banbura M., Kalinski A., Markowski M., Skwarska J., Wawrzyniak J., Zielinski P., Cyzewska I., Mankowska D., Banbura J. Effects of human-related disturbance on breeding success of urban and non-urban blue tits (Cyanistes caeruleus). Urban Ecosystems 2016, 19, published online: DOI 10.1007/s11252-016-0543-3.

[18] Bańbura J., Bańbura M. Blue tits Cyanistes caeruleus and great tits Parus major as urban habitat breeders. Inter Studies Sparrows 2012, 36: 66-72.

[19] Pütter A. Studien über physiologische Ähnlichkeit. VI. Wachstumsähnlichkeiten. Pflügers Archiv für die Gesamte Physiologie des Menschen und der Tiere 1920, 180: 298-340.

[20] Wu K., Darcet D., Wang Q., Sornette D. Generalized logistic growth modeling of the COVID-19 outbreak in 29 provinces in China and in the rest of the world. Nonlinear Dynamics 2020, published online: DOI 10.1007/s11071-020-05862-6.

[21] Zreiq R, Kamel S, Boubaker S, Al-Shammary AA, Algahtani FD, Alshammari F. Generalized Richards model for predicting COVID-19 dynamics in Saudi Arabia based on particle swarm optimization algorithm. AIMS Public Health 2020, 7: 828-843.

[22] Brunner N., Kühleitner M. Forecasting the final disease size: comparing calibrations of Bertalanffy–Pütter models. Epidemiology and Infection 2021, 149, published online: DOI 10.1017/S0950268820003039.

[23] Pauly D., Cheung W.W.L. Sound physiological knowledge and principles in modeling shrinking of fishes under climate change. Global Change Biology 2018, 24, published online: DOI 10.1111/gcb.13831.

[24] Renner-Martin K., Brunner N., Kühleitner M., Nowak W.G., Scheicher K. On the Exponent in the Von Bertalanffy Growth Model. PeerJ 2018, 6, published online: DOI 10.7717/peerj.4205.

[25] Renner-Martin K., Brunner N., Kühleitner M., Nowak W.G., Scheicher K. Best-fitting Growth Curves of the Von Bertalanffy-Pütter Type. Poultry Science 2019, 98: 3587-3592.

[26] Brunner N., Kühleitner M., Nowak W.G., Renner-Martin K., Scheicher K. Comparing growth patterns of three species: Similarities and differences. PLoS ONE 2019, 14, published online: DOI 10.1371/journal.pone.0224168.

[27] Brunner N., Kühleitner M. The growth of domestic goats and sheep: A meta study with Bertalanffy-Pütter models. Veterinary and Animal Science 2020, 10, published online: DOI 10.1016/j.vas.2020.100135.

Formatted: List Paragraph, Indent: Left: 0", Numbered + Level: 1 + Numbering Style: 1, 2, 3, … + Start at: 1 + Alignment: Left + Aligned at: 0.25" + Indent at: 0.5"

Formatted: List Paragraph, Indent: Left: 0", Numbered + Level: 1 + Numbering Style: 1, 2, 3, … + Start at: 1 + Alignment: Left + Aligned at: 0.25" + Indent at: 0.5"

Formatted: List Paragraph, Indent: Left: 0", Numbered + Level: 1 + Numbering Style: 1, 2, 3, … + Start at: 1 + Alignment: Left + Aligned at: 0.25" + Indent at: 0.5"

[28] Corsini M., Dubiec A., Marrot P., Szulkin M. Humans and Tits in the City: Quantifying the Effects of Human Presence on Great Tit and Blue Tit Reproductive Trait in Variation. Frontiers in Ecology and Evolution 2017, 5, published online: DOI 10.3389/fevo.2017.00082.

[29] Corsini M., Marrot P., Szulkin M. Quantifying human presence in a heterogeneous urban landscape. Behavioral Ecology 2019, 30: 1632-1641.

[30] Corsini M., Schöll E.M., Di Lecce I., Chatelain, M., Dubiec A., Szulkin M. Growing in the city: urban evolutionary ecology of avian growth rates. Evolutionary Applications 2020, published online: DOI 10.1111/eva.13081.

[31] Zach R. Growth-curve analysis: A critical reevaluation. Auk 1988, 105: 208-210.

[32] Tjørve K.M.C., Tjørve E. (2010) Shapes and functions of bird-growth models: how to characterize chick postnatal growth. Zoology 2010, 113: 326-333.

[33] Kuhi H.D., Porter T., López S., Kebreab E., Strathe A., Dumas A., France J. A review of mathematical functions for the analysis of growth in poultry. Worlds Poultry Science Journal 2010, 66: 227-240.

[34] Gompertz B. On the nature of the function expressive of the law of human mortality, and on a new mode of determining the value of life contingencies. Philos. Trans. R. Soc. London 1832, 123: 513-585.

[35] Brody S. Bioenergetics and Growth. Hafner Publ. Comp. 1945, New York, NY, USA.

[36] Bertalanffy L.v. Problems of organic growth. Nature 1949, 163: 156-158.

[37] Bertalanffy L.v. Quantitative laws in metabolism and growth. Quarterly Revues in Biology 1957, 32: 217-231.

[38] West G.B., Brown J.H., Enquist B.J. A general model for ontogenetic growth. Nature 2001, 413:628-631.

[39] West G.B., Brown J.H., Enquist B.J. Growth models based on first principles or phenomenology? Functional Ecology 2004, 18: 188-196.

[40] Katsanevakis S., Maravelias C.D. Modelling fish growth: multi-model inference as a better alternative to a priori using von Bertalanffy equation. Fish and Fisheries 2008, 9:178-187.

[41] Manabe A., Yamakawa T., Ohnishi S., Akamine T., Narimatsu Y., Tanaka H., Funamoto T., Ueda Y., Yamamoto T. A novel growth function incorporating the effects of reproductive energy allocation. PLoS ONE 2018, 13, published online: DOI 10.1371/journal.pone.0199346

[42] Jacobs J., Rockne R.C., Hawkins-Daarud A.J., Jackson P.R., Johnston S.K., Kinahan P., Swanson K.R. Improved model prediction of glioma growth utilizing tissue-specific boundary effects. Mathematical Biosciences 2019, 312: 59-66.

[43] Marusic M., Bajzer Z. Generalized Two-Parameter Equations of Growth. Journal of Mathematical Analysis and Applications 1993, 179: 446-462.

[44] Richards F.J. A flexible growth function for empirical use. Journal of Experimental Botany 1959, 10: 290-300.

[45] Tjørve K.M.C., Tjørve E. (2017) Modelling avian growth with the Unified-Richards: As exemplified by wader-chick growth. Journal of Avian Biology 2017, 48: 770-784.

[46] Fernandes F.A., Silva E.M., Lima K.P., Jane S.A., Fernandes T.J., Muniz J.A. Parameterizations of the von Bertalanffy model for description of growth curves. Revista Brasileira de Biometrica 2020, 38: 369-384.

[47] Ohnishi S., Yamakawa T., Akamine T. On the Analytical Solution for the Pütter-Bertalanffy Growth Equation. Journal of Theoretical Biology 2014, 343: 174-177.

[48] Erdélyi A., Magnus, W., Oberhettinger F., Tricomi F.G. Higher transcendental functions I. McGraw-Hill Book Company 1953, New York, USA.

Formatted: List Paragraph, Indent: Left: 0", Numbered + Level: 1 + Numbering Style: 1, 2, 3, … + Start at: 1 + Alignment: Left + Aligned at: 0.25" + Indent at: 0.5"

Formatted: List Paragraph, Indent: Left: 0", Numbered + Level: 1 + Numbering Style: 1, 2, 3, … + Start at: 1 + Alignment: Left + Aligned at: 0.25" + Indent at: 0.5"

Formatted: List Paragraph, Indent: Left: 0", Numbered + Level: 1 + Numbering Style: 1, 2, 3, … + Start at: 1 + Alignment: Left + Aligned at: 0.25" + Indent at: 0.5"

[49] Fernandes F.A., Fernandes T.J., Pereira A.A., Meirelles S.L.C., Costa A.C. Growth curves of meat-producing mammals by von Bertalanffy's model. Pesquisa Agropecuária Brasileira 2019, 54, published online: DOI 10.1590/s1678-3921.pab2019.v54.01162.

[50] Stewart J., Robbins W.D., Rowling K., Hegarty A., Gould A. A multifaceted approach to modelling growth of the Australian bonito, Sarda australis (Family Scombridae), with some observations on its reproductive biology. Marine and Freshwater Research 2013, 64: 671-678.

[51] White C.R. Physiology: there is no single p. Nature 2010, 464: 691-693.

[52] Austin S.H., Robinson T.R., Robinson W.D., Ricklefs R.D. Potential biases in estimating the rate parameter of sigmoid growth functions. Methods in Ecology and Evolution 2011, 2: 43-51.

[53] Naef-Daenzer B., Keller L.K. The foraging performance of great and blue tits (Parus major and P. caeruleus) in relation to caterpillar development, and its consequences for nestling growth and fledging weight. Journal of Animal Ecology 1999, 68: 708-718.

[54] Calder WA III Size, Function, and Life History. Harvard Univ. Press 1985, Cambridge, MA, USA.

[55] Aggrey S.E. Comparison of three nonlinear and spline regression models for describing chicken growth curves. Poultry Science 2002, 81:1782-1788.

[56] Maceina M.J. Use of piecewise nonlinear models to estimate variable size-related mortality rates. North American Journal of Fisheries Management 2007, 27:971-977.

[57] Hsing Y. Economic growth and income inequality: the case of the US. International Journal of Social Economics 2005, 32: 639-647.

[58] Garcia O. A stochastic differential equation model for the height growth of forest stands. Biometrics 1983, 39: 1059-1072.

[59] Pinheiro J.C., Bates D.M. Mixed-Effects Models in S and S-Plus. Springer Publishing 2000, New York, USA.

[60] Serroyen J., Molenberghs G., Verbek G., Davidian M. Non-linear Models for Longitudinal Data. American Statistician 2009, 63: 378-388.

[61] Strathe A.B., Danfær A., Sørensen H., Kebreab E. A multilevel nonlinear mixed effects approach to model growth in pigs. Journal of Animal Science 2010, 88: 638-649.

[62] Sofaer H.R., Chapman P.L., Sillett T.S., Ghalambor C.K. Advantages of nonlinear mixed models for fitting avian growth curves. Journal of Avian Biology 2013, 44: 469-478.

[63] Svagelj W.S., Laich A.G., Quintana F. Richards's equation and nonlinear mixed models applied to avian growth: why use them? Journal of Avian Biology 2019, 50, published online: DOI 10.1111/jav.01864.

[64] Gustavsson S., Fagerberg B., Sallsten G., Andersson E.M. Regression models for log-normal data: comparing different methods for quantifying the association between abdominal adiposity and biomarkers of inflammation and insulin resistance. International Journal of Environmental Research and Public Health 2014, 11: 3521-3539.

[65] Box G.E.P., Cox, D.R. An analysis of transformations. Journal of the Royal Statistical Society 1964, B26: 211-252.

[66] Brown W.P., Eggermont P., LaRiccia V., Roth R.R. Are parametric models suitable for estimating avian growth rates? Journal of Avian Biology 2007, 38: 495-506.

[67] Ogle D.H. Introductory Fisheries Analyses with R. CRC Press 2016, Boca Raton, FL, USA.

[68] Oswald S.A., Nisbet I.C.T., Chiaradia A., Arnold J.M. FLEXPARAMCURVE: R package for flexible fitting of nonlinear parametric curves. Methods in Ecology and Evolution 2012, 3: 1073-1077.

Formatted: List Paragraph, Indent: Left: 0", Numbered + Level: 1 + Numbering Style: 1, 2, 3, … + Start at: 1 + Alignment: Left + Aligned at: 0.25" + Indent at: 0.5"

[69] Shi P.J., Ishikawa T., Sandhu H.S., Hui C., Chakraborty A., Jin X.S., Tachihara K., Li B.L. On the 3/4-exponent van Bertalanffy equation for ontogenetic growth. Ecological Modelling 2014, 276: 23-28.
[70] Loibel S., Andrade M.G., do Val J.B.R., de Freitas A.R. Richards growth model and viability indicators for populations subject to interventions. Anais da Academia Brasileira de Ciências 2010, 82, published online: DOI 10.1590/S0001-37652010000400028.
[71] Grossman M. Multiphasic analysis of growth curves in chicken. Poultry Science Verhulst P.F. (1838)1988, 67: 33-42.

Notice sur la loi que la population suit dans son accroissement. Curr. Math. Phys. 10:113-121.

[72] Vidal R.V.V. (1993) Applied Simulated Annealing. In: Lecture notes in economics and mathematical systems. Berlin: Springer- Verlag 1993, Berlin, Germany.
[73] Spiess A.N., Neumeyer N. An evaluation of R^2 as an inadequate measure for nonlinear models in pharmacological and biochemical research: a Monte Carlo approach. BMC Pharmacology 2010, 10, published online: DOI 10.1186/1471-2210-10-6.
[74] Burnham K.P., Anderson D.R. Multi-model inference. Understanding AIC and BIC in model selection. Sociological Methods and Research 2004, 33: 261-304.
[75] Hollander M.A., Wolfe D.E. Nonparametric Statistical Methods, Wiley 1999, Chichester, UK.
[76] Anderson T.W., Darling D.A. Asymptotic theory of certain "goodness-of-fit" criteria based on stochastic processes. Annals of Mathematical Statistics 1952, 23: 193-212.
[77] Brisbin Jr.I.L., Collins C.T., White G.C., McCallum D.A. A new paradigm for the analysis and interpretation of growth data: The shape of things to come. Auk 1987, 104: 552-554.
[78] Vrána J., Remeš V., Matysioková B., Tjørve K.M.C., Tjørve E. (2019) Choosing the right sigmoid growth function using the unified-models approach. Ibis 2019, 161: 13-26.

Formatted: List Paragraph, Indent: Left: 0", Numbered + Level: 1 + Numbering Style: 1, 2, 3, … + Start at: 1 + Alignment: Left + Aligned at: 0.25" + Indent at: 0.5"

Formatted: List Paragraph, Indent: Left: 0", Numbered + Level: 1 + Numbering Style: 1, 2, 3, … + Start at: 1 + Alignment: Left + Aligned at: 0.25" + Indent at: 0.5"

Table

West G.B., Brown J.H., Enquist B.J. (2001) A general model for ontogenetic growth. Nature 413:628-631.

West G.B., Brown J.H., Enquist B.J. (2004) Growth models based on first principles or phenomenology? Functional Ecology 18: 188-196.

White C.R. (2010) Physiology: there is no single p. Nature 464: 691-693.

Tables

Formatted: Font: Not Bold

Formatted: Line spacing: Double

Table 1. RangeMedians of the environmental data for the nest-box neighborhoodseach group of birds and counts of above/below median birds.

Indicator	BT-F			BT-M			GT-F			GT-M		
	Minimum	Median	Maximum	Minimum	Median	Maximum	Minimum	Median	Maximum	Minimum	Median	Maximum
Hatching day[1]	31 d	38 d	43 d	36 d	38 d	60 d	33 d	36 d	45 d	33 d	38 d	45 d
Hatchlings[2]	5	9	12	5	7	12	4	7	8	4	7	8
Human activity[3]	0.1	1.1	2.1	0	1.05	2.1	0	1.1	3.35	0	0.75	3.35
ISA[4]	0.57%	4.59%	26.26%	0.57%	4.49%	26.26%	5.12%	10.38%	21.94%	5.12%	9.07%	21.94%
Light[5]	2399 lx	3160 lx	11,463 lx	2384 lx	2852 lx	11,463 lx	2539 lx	8588 lx	13,848 lx	2539 lx	4907 lx	13,848 lx
NDVI[6]	0.49	0.76	0.81	0.49	0.75	0.81	0.65	0.69	0.82	0.65	0.71	0.82
Path[7]	6 m	14.3 m	108.5 m	4.3 m	11.1 m	31.5 m	3.8 m	5.55 m	48.3 m	3.8 m	6.9 m	48.3 m
Road[8]	2 m	85.4 m	256.3 m	2 m	165.1 m	256.3 m	32 m	63.7 m	152.8 m	32 m	106.9 m	152.8 m
Sound[9]	62.06 db	65.44 db	74.63 db	62.06 db	65.93 db	74.63 db	62.46 db	65.41 db	74.85 db	62.46 db	66.98 db	74.85 db
Temperature[10]	10.37°C	10.86°C	12.03°C	10.37°C	10.86°C	14.16°C	10.62°C	11.04°C	12.96°C	10.62°C	11.08°C	12.96°C
Tree cover[11]	3.26%	28.13%	51.94%	2.29%	19.07%	51.94%	22.84%	30.46%	59.12%	12.47%	24.90%	59.12%

Indicator	Female blue tits	count[12]	Male blue tits	count[12]	Female great tits	Male great tits
Hatching day[1]	38 d	A: 15, B: 11	38 d	A: 24, B: 7	36 d	38 d
Hatchlings initially[2]	9	A: 20, B: 6	7	A: 25, B: 6	7	7
Hatchlings finally[2]	6	A: 15, B: 11	5	A: 17, B: 14	5	4
Nest-size difference[2]	5	A: 17, B: 9	1	A: 23, B: 8	1	1
Human activity[3]	1.1	A: 15, B: 11	1.05	A: 20, B: 11	1.1	0.75
ISA[4]	4.59%	A: 13, B: 13	4.49%	A: 20, B: 11	10.38%	9.07%
Light[5]	3160 lx	A: 14, B: 12	2852 lx	A: 20, B: 11	8588 lx	4907 lx
NDVI[6]	0.76	A: 14, B: 12	0.75	A: 17, B: 14	0.69	0.71
Path[7]	14.3 m	A: 11, B: 15	11.1 m	A: 17, B: 14	5.55 m	6.9 m
Road[8]	85.4 m	A: 15, B: 11	165.1 m	A: 18, B: 13	63.7 m	106.9 m

Sound[9]	65.44 db	A: 13, B: 13	65.93 db	A: 16, B: 15	65.41 db	66.98 db
Temperature[10]	10.86°C	A: 17, B: 9	10.86°C	A: 20, B: 11	11.04°C	11.08°C
Tree cover[11]	28.13%	A: 14, B: 12	19.07%	A: 16, B: 15	30.46%	24.90%

Notes: All numbers were rounded to the shown decimal. ~~Environmental indicators in alphabetic order:~~ [1] count of days from the start of the field work to hatching; [2] initial ~~count~~and final counts, respectively, of hatchlings in each nest, and difference of these counts; [3] index of human activity from Corsini et al. (2017); [4] percentage of impervious area around the nest-box; [5] light pollution at the nest-box site (in lx); [6] index for the vegetation cover around the nest-box; [7] minimal (Euclidean) distance to the (center of the) next path (in m); [8] minimal (Euclidean) distance from the nest-box to (the center of) the next road (in m); [9] sound pollution at the nest-box site (in db); [10] average of the morning temperatures at the eight visits (in °C); [11] tree cover around the nest-box (percent~~).~~); [12] count of birds of the given type (first row, left column), whose nest characteristics was above (A)/below (B) the median (displayed in the left column). Counts for great tits are not displayed, because for 12 birds per group the subsamples were too small to be representative.

Formatted: Tab stops: Not at 5.16"

Formatted: Line spacing: Double, Tab stops: Not at 5.16"

Table 2. Range of the model parameters for the sample of 81 birds.

Parameter		BT-F			BT-M			GT-F			GT-M	
	Minimum	Median	Maximum	Minimum	Median	Maximum	Minimum	Median	Maximum	Minimum	Median	Maximum
Exponent *a*	0	0.495	1.61	0.05	0.63	1.52	0.01	0.815	1.24	0.04	0.835	1.6
Exponent *b*	0.01	2.28	23.53	0.44	2.31	18.25	1.33	3.155	7.42	1.17	3.69	11.28
Initial mass *c*	0.80 g	1.01 g	1.51 g	0.80 g	1.10 g	1.79 g	1.00 g	1.50 g	1.77 g	1.00 g	1.26 g	1.57 g
Parameter *p*	0.32	0.71	20.79	0.31	0.67	2.05	0.34	0.91	1.52	0.30	0.96	8.38
Parameter *q*	1.15E-22	0.01	20.01	1.59E-18	0.01	1.54	4.59E-09	0.002	1.04	1.56E-13	6.79E-05	8.14
SSLE	0.0018	0.0107	0.0481	0.0010	0.0087	0.1638	0.0049	0.0113	0.0221	0.0008	0.0111	0.0258
Difference *b*–*a*	0.01	1.8	23.21	0.01	1.67	18	0.14	2.51	7.41	0.01	3.29	10.82
Mass m_{max}	6.290 g	9.225 g	45.848 g	8.451 g	10.190 g	1.41E+114 g	13.39 g	15.00 g	18.26 g	13.93 g	15.63 g	19.29 g
Time t_{infl}	0 d	4.804 d	6.867 d	0	5.075 d	7.32 d	3.35 d	5.03 d	7.46 d	3.59 d	5.38 d	8.67 d
Mass m_{infl}	0 g	4.809 g	7.169 g	0	5.337 g	7.17 g	5.48 g	7.20 g	9.68 g	6.63 g	8.29 g	10.73 g
Ratio m_{infl}/m_{max}	0	0.51	0.83	0	0.50	0.79	0.41	0.46	0.67	0.39	0.53	0.74

Note: All numbers rounded to the shown decimals. Parameter *q* and mass m_{max}: x.xxEy means x.xx times 10^y. Time t_{infl}: Day 1 of the data means time t = 0 of the model; t_{infl} = 0 means no inflection point. Mass m_{infl}: 0 means no inflection point.

Table 3. Spearman rho and significance.

Indicator	Comparing indicator with inflection-point-mass over asymptotic mass				Comparing indicator with the exponent-difference			
	BT-F (26)	BT-M (31)	GT-F (12)	GT-M (12)	BT-F (26)	BT-M (31)	GT-F (12)	GT-M (12)
Hatching day	not significant	not significant	not significant	not significant	0.48 (1.394%)	not significant	−0.59 (4.236%)	not significant
Hatchlings	−0.59 (0.152%)	not significant	−0.86 (0.033%)	−0.78 (0.269%)	not significant	not significant	not significant	not significant
Human activity	not significant	not significant	not significant	not significant	not significant	not significant	not significant	not significant
ISA	−0.61 (0.091%)	−0.49 (0.537%)	not significant	not significant	not significant	not significant	0.71 (0.899%)	not significant
Light	−0.60 (0.133%)	−0.55 (0.134%)	not significant	not significant	−0.61 (0.0935%)	−0.43 (1.514%)	not significant	not significant
NDVI	not significant	not significant	not significant	0.70 (1.106%)	not significant	not significant	not significant	not significant
Path	not significant	not significant	not significant	not significant	not significant	0.42 (2.024%)	−0.82 (0.123%)	−0.65 (2.292%)
Road	not significant	not significant	not significant	not significant	not significant	not significant	not significant	not significant
Sound	−0.48 (1.321%)	not significant	not significant	not significant	not significant	not significant	not significant	not significant
Temperature	not significant	not significant	not significant	not significant	not significant	not significant	not significant	not significant
Tree cover	not significant	not significant	not significant	not significant	not significant	not significant	not significant	not significant

Note: Indicators are explained in Table 1. Entries "x (y%)" inform about the value x of Spearmann rho and the P-value y for the Spearman rank test, whereby the second row informs about the considered sample and the first column informs about the indicator that was compared with m_{infl}/m_{max} and $b-a$, respectively; "not significant" indicates a P-value y > 5%.

Table 4. Comparing the medians of the environmental indicators for different groups of birds.

Indicator	High-ratio (H) compared to low-ratio (L) birds				Near-diagonal (N) compared to far-diagonal (F) birds			
	BT-F (15 H, 11 L)	BT-M (19 H, 12 L)	GT-F (3 H, 9 L)	GT-M (7 H, 5 L)	BT-F (13 N, 13 F)	BT-M (15 N, 16 F)	GT-F (5 N, 7 F)	GT-M (5 N, 7 F)
Hatching day	not significant	not significant	not significant	not significant	36 d< 38 d (0.199%)	Medians 38 d; mean 40.2 d > 40.19 d (4.5%)	36 > 33 (4.794%)	not significant
Hatchlings	9 < 11 (0.346%)	not significant	4 < 8 (2.35%)	4 < 8 (2.536%)	not significant	not significant	not significant	not significant
Human activity	not significant	not significant	not significant	not significant	not significant	1.05 < 1.8 (1.607%)	0 < 1.1 (4.528%)	not significant
ISA	4.5% < 11.7% (0.663%)	3.6% < 8.9% (1.137%)	not significant	not significant	not significant	5.5% > 3.6% (3.671%)	5.1% < 11.7% (0.457%)	not significant
Light	not significant	2852 lx < 3160 lx (2.0%)	not significant	not significant	4420 lx > 2826 lx (1.426%)	not significant	4907 lx < 9988 lx (4.528%)	not significant
NDVI	not significant	not significant	not significant	0.78 > 0.69 (4.728%)	not significant	not significant	not significant	not significant
Path	not significant	not significant	not significant	not significant	not significant	6 m < 19 m (0.067%)	48.3 m > 3.8 m (0.267%)	not significant
Road	not significant	not significant	not significant	not significant	not significant	not significant	106.9 m > 44.2 m (1.951%)	not significant
Sound	64.0 db < 68.3 db (0.565%)	not significant	74.9 db > 65.4 db (0.783%)	not significant	not significant	not significant	62.5 db < 65.4 db (3.01%)	not significant
Temperature	not significant	not significant	not significant	not significant	not significant	not significant	not significant	not significant
Tree cover	not significant	not significant	22.8% < 30.5% (1.354%)	not significant	not significant	not significant	59.1% > 30.5% (1.951%)	not significant

Note: x<y (z%) informs about the median of the indicator values x for H (N), y for L (F) and about the P-value of the Mann-Whitney test to for a significant difference between the medians.

Table 5. Comparing the medians of ratios and exponent differences of two groups of birds defined by ambient conditions above or below the median indicator value.

Indicator	BT-F			BT-M		
	counts	Medians: ratio	Medians: exp. diff.	counts	Medians: ratio	Medians: exp. diff.
Hatching day	H: 7, L: 19	not significant	not significant	H: 7, L: 24	not significant	not significant
Hatchlings	H: 9, L: 17	unbalanced	not significant	H: 14, L: 17	not significant	not significant
Human activity	H: 12, L: 14	not significant	not significant	H: 15, L: 16	not significant	3 > 0.22 (2.815%)
ISA	H: 13, L: 13	0.467 < 0.529 (0.868%)	not significant	H: 15, L: 16	0.45 < 0.561 (0.962%)	not significant
Light	H: 12, L: 14	0.459 < 0.544 (1.231%)	0.155 < 5.48 (0.184%)	H: 15, L: 16	0.483 < 0.529 (2.303%)	not significant
NDVI	H: 12, L: 14	not significant	not significant	H: 15, L: 16	not significant	not significant
Path	H: 11, L: 15	not significant	not significant	H: 15, L: 16	not significant	not significant
Road	H: 12, L: 14	not significant	4.835 > 0.185 (3.705%)	H: 13, L: 18	not significant	not significant
Sound	H: 13, L: 13	0.467 < 0.529 (3.068%)	not significant	H: 12, L: 19	unbalanced	not significant
Temperature	H: 12, L: 14	not significant	not significant	H: 12, L: 19	not significant	not significant
Tree cover	H: 10, L: 16	not significant	not significant	H: 13, L: 18	not significant	not significant
Indicator	GT-F			GT-M		
	counts	Medians: ratio	Medians: exp. diff.	counts	Medians: ratio	Medians: exp. diff.
Hatching day	H: 3, L: 9	not significant	not significant	H: 6, L: 6	not significant	not significant
Hatchlings	H: 5, L: 7	0.433 < 0.481 (0.577%)	not significant	H: 5, L: 7	0.45 < 0.544 (2.299%)	not significant
Human activity	H: 3, L: 9	not significant	not significant	H: 4, L: 8	not significant	not significant
ISA	H: 6, L: 6	not significant	5.025 > 0.245 (1.307%)	H: 5, L: 7	not significant	not significant
Light	H: 6, L: 6	not significant	5.025 > 0.245 (1.307%)	H: 5, L: 7	not significant	not significant
NDVI	H: 5, L: 7	not significant	0.23 < 4.56 (2,299%)	H: 6, L: 6	0.592 > 0.454 (4,533%)	not significant
Path	H: 6, L: 6	not significant	0.245 < 5.025 (0.824%)	H: 5, L: 7	not significant	0.14 < 4.5 (1.168%)
Road	H: 6, L: 6	not significant	0.245 < 5.025 (0.824%)	H: 5, L: 7	not significant	not significant
Sound	H: 4, L: 8	not significant	not significant	H: 5, L: 7	not significant	not significant
Temperature	H: 3, L: 9	not significant	not significant	H: 6, L: 6	not significant	not significant
Tree cover	H: 4, L: 8	not significant	0.21 < 4.355 (0.847%)	H: 5, L: 7	not significant	not significant

Note: counts "H: x, L: y" are the number x of birds, with H (the nest size had an indicator value larger than the median), and the number y of birds with L (the indicator was lower than or equal to the median), whereby the median of Table 1 was used (different medians of each indicator for species and sex); unbalanced indicates a significant difference, but the sample sizes for the above and below median birds differed by more than 2; x < y (z) informs about the medians of the ratios m_{infl}/m_{max} and the exponent difference $b-a$, respectively, of the H and L groups, respectively, and z is the P-value of the Mann-Whitney test.

Figure captions

Formatted: Line spacing: Double

Figure 1: Weather data during May and June of 2016, showing minimal and maximal daily temperatures (°C) in blue and red, using the left axis, maximal daily precipitation rate (mm/h) in grey, using the right axis; source: open data from EPWA weather station (Warsaw Chopin airport), retrieved using Mathematica software.

Figure 2: Mass at days 1, 3, ..., and 15 plotted side-by-side; from left BT-F (red), BT-M (blue), GT-F (orange), and GT-M (cyan).

Figure 3: Growth trajectories of logistic growth and an alternative growth model andtogether with the inflection points. The growth curves had the parameters a = 1, b = 2, c = 0.1, p = q = 1 (blue: logistic growth) and a = 1, b = 4, c = 0.1, p = q = 0.7526 (red: alternative growth).). The asymptotic limit was 1 (both curves) and the inflection point coordinates (gray dots) were (2.197, 0.5) for logistic growth and (2.572, 0.63) for the alternative curve.

Formatted: Font: Not Italic
Formatted: Font: Not Italic
Formatted: Font: Not Italic
Formatted: Font: Not Italic
Formatted: Font: Not Italic
Formatted: Font: Not Italic
Formatted: Font: Not Italic
Formatted: Font: Not Italic
Formatted: Font: Not Italic
Formatted: Font: Not Italic

Figure 2: Mass at days 1, 3, ..., and 15 plotted side-by-side; from left blue tits-F (red), blue tits-M (blue), great tits-F (orange), and great tits-M (cyan).

Figure 3: Mass of bird K7V3513 at days 1, 3, ..., and 15 (plotted at t = 0, 1, ..., 14) and best-fitting BP-model growth curve (parameters a = 0.8, b = 3.8, c = 1.01, p = 0.4, q = 0.0007) with SSLE = 0.006.

Figure 4: Best-fit exponent-pairs for 81 nestlings (color code as in Figure 21) and yellow lines (b = a and b = a+1.5) to indicate near-diagonal exponent-pairs.

Formatted: Font: Not Italic
Formatted: Font: Not Italic
Formatted: Font: Not Italic
Formatted: Font: Not Italic

Figure 5: Exponent-pairs of named models (blue), exponent-pairs of models with RL > 98% for 78 data (red), exponent-pairs of models with RL > 95% for 80 data (green), remaining exponent-pairs of the search grid (yellow), diagonal a = b (grey) and contour lines (thick and black) of the ratios 0.2, 0.3, ..., 0.6 (from left to right), using equation (2) for the ratio.

Formatted: Line spacing: Double, Tab stops: Not at 5.16"

	**Editor**	**Authors**
01	Thank you for submitting your manuscript to PLOS ONE. After careful consideration, we feel that it has merit but does not fully meet PLOS ONE's publication criteria as it currently stands. Therefore, we invite you to submit a revised version of the manuscript that addresses the points raised during the review process.	Changes are described below
02	Please ensure that your manuscript meets PLOS ONE's style requirements, including those for file naming. The PLOS ONE style templates can be found at https://journals.plos.org/plosone/s/file?id=wjVg/PLOSOne_formatting_sample_main_body.pdf https://journals.plos.org/plosone/s/file?id=ba62/PLOSOne_formatting_sample_title_authors_affiliations.pdf	done
03	Please amend the manuscript submission data (via Edit Submission) to include author Katharina Renner-Martin.	done
04	We note that Figure S1 n your submission contains satellite images which may be copyrighted.	Please note that we have resubmitted a completely new set of supporting information files. We removed the satellite image, as the site could be located easily from a Google search.
	**Review 1**	**Authors**
05	This paper presents an application of the von Bertalanffy-Pütter growth curve for analyzing differences in growth parameters (chiefly the position of the inflection point) for passerines. There has been much discussion through the years since the initial work of Ricklefs on the most suitable growth curve to use for different species and applications. The strength of this approach lies in the ability to alter *minfl/mmax,* the position of the inflection point proportional to the extent of growth (most common parametric curves are limited to inflection occurring at a specific point in growth: e.g. 50% in the logistic model).	OK
06	I find the approach appealing. I agree with the argument that a flexible *minfl/mmax* ratio might have biological relevance in some instances of growth. I am also happy that curve-fitting was undertaken for each individual separately, as it is clear that the long tradition of pooling masses across individuals misses biologically important differences as it treats all individuals as equal.	OK
07	I found the paper well written and easy to follow	OK
08	but hard to evaluate because the data, although analyzed per individual, were never directly	We have obtained permission to publish the original data; they are supplied as supporting

	presented in that form: Fig 2 presents individual data but it is not clear which belong to which individual and thus to gauge individual growth trajectories.	information
09	Corsini et al. only report mass at age 15 – near fledging, and they also say that nestlings were ringed (banded) at age 15d. (This begs the question how could they identify individual chicks prior to that point to be able to collect growth on individuals?). It would be beneficial to know how nestlings were weighed and the accuracy of the instruments used to weigh them. I presume this will be rectified when data are deposited but it makes evaluation of the method harder.	The nestlings were marked at the first weighing.
10	My second concern is that the conclusion at the end of the paper: "as noted in the introduction, for many common three-parameter models of avian literature the ratio is fixed (e.g. ratio 0.5 for logistic growth). We conclude that these more traditional three-parameter models are not suitable to explore and compare the shapes of growth curves". I believe that this statement is too strong. Depending on the application of the growth curve a simpler curve may be just as appropriate and since it could be implemented in a well-developed analytical framework, such as mixed-effects models, it might be easier to use.	We modified our conclusion to point out its context.
11	I am very interested in the B-P approach but having to learn another program (Mathematica) just to implement it, is discouraging. From my experience, the majority of ornithologists use R for statistics and thus converting this code into an R package would greatly help foster its use by ornithologists.	The authors would support anyone interested in translating the m-codes into R. However, the authors are not natives of R.
12	One thing that is common is that as more parameters are estimated in a curve there is a greater potential for over-fitting, as changing one parameter can have a knock-on effect on another. I am familiar with this for the 4-parameter Richards curve and imagine that the B-P (4 or 5-parameters in eqn 1 in the paper) would suffer from the same issues. Thus, it is conceivable that different ratios of *minfl/mmax* with different parameters for c, p and q might achieve similar shaped curves. This would imply that just because the ratio of *minfl/mmax* has sufficiently different values for individuals or groups does not *necessarily* mean that it reflects the same differences in growth curve. I think what is needed is a figure (or even supplementary material) that shows there is consistency (or at least a limit to the range of variation) in curve shape across different ratios of *minfl/mmax*.	We have followed up this suggestion and presented more details of the following results in the paper: The observation of the reviewer is insofar true, as the exponent-differences and ratios m_{infl}/m_{max} varied widely amongst three-parameter models with a good fit to the data. However, for the ratios (but not for the exponent differences) the median of the ratios of good-fitting models was close to the ratio of the best-fit model. Thus, the ratios appear to have a biological meaning, while the exponent-differences remained somewhat evasive. We therefore removed the discussion of the exponent-difference (where owing to the high variability there were barely significant associations). Thank you for this valuable suggestion.
13	I also strongly believe that to truly show the B-P	Clearly the 5-parameter model necessarily

	method is superior in the current application to two species of tits that the other 3-parameter curves (and/or Richards curve) need to be fit and compared to the B-P results. You could argue (perhaps by providing more details) that previous publications give support for the fact that B-P fit better than 3-parameter curves and so this is not necessary. I would still suggest at this stage to play it safe and demonstrate this (even if just in supplementary material) for the data set at hand.	has a better fit than any 3-parameter special case. However, as recommended in #15, the issue should not be the superiority of the BP-models over others, but its utility for the specific research question, the study of the ratio. Here the 5-parameter model is insofar superior, as the very topic would be beyond the reach of the 3-parameter models, which assume fixed ratios. We changed the paper accordingly.
14	It would also be nice to provide an indication of approximately how many data points are needed for a good fit: with a 4-parameter Richards curve clearly 6+ is desirable. The need for a large number of data points well-spaced through development clearly precludes using the method in many cases where data are more irregularly collected.	We used only data with eight points of time and required a certain regular scheme of weighing; all other (=most) data were discarded. As one PC could fit only 1-2 data per week, we reserved the available time for clearly comparable data, only.
15	Third, although stated in the 5th paragraph of the Introduction, the precise purpose of this paper [in how it differs from similar explorations for poultry (Kühleitner et al. 2019), poultry and dinosaurs (Brunner et al. 2019), and fish (Renner-Martin et al. 2018)] is currently not immediately forthcoming. I am interpreting it as: the need to show that differences in the ratio of *minfl*/*mmax* have ecological implications. If this is the case, please state it more strongly in the abstract and introduction (and discussion) rather than focusing on the B-P as a superior method.	We changed this accordingly: The issue of the paper is the consideration of the ratio and this requires a model that is flexible with respect to this ratio.
16	I also believe that the paper would benefit from reference to some of the following ornithological literature on growth curves that is missing from the Introduction:	We considered the literature as follows
17	Brisbin Jr., I.L., C.T. Collins, G.C. White and D.A. McCallum. 1987. A new paradigm for the analysis and interpretation of growth data: The shape of things to come. Auk 104: 552–554.	Cited in the results
18	Oswald, S.A., I.C.T. Nisbet, A. Chiaradia and J.M. Arnold. 2012. FlexParamCurve: R package for flexible fitting of nonlinear parametric curves. Methods in Ecology and Evolution 3: 1073–1077.	Cited in the methods
19	Remeŝ, V. and T.E. Martin. 2002. Environmental influences on the evolution of growth and developmental rates in passerines. Evolution 56: 2505–2518.	Cited in the methods
20	Sofaer, H.R., P.L. Chapman, T.S. Sillett and C.K. Ghalambor. 2013. Advantages of nonlinear mixed models for fitting avian growth curves. Journal of Avian Biology 44: 469–478.	Cited in the methods
21	Svagelj, W. S., Laich, A. G., & Quintana, F. (2019). Richards's equation and nonlinear mixed models applied to avian growth: why use them? Journal of	Cited in the methods

	Avian Biology, 50: e01864.	
22	Zach, R. 1988. Growth-curve analysis: A critical reevaluation. Auk 105: 208–210.	Cited in the introduction
23	Fourth, I find the "shotgun" style statistical treatment in "Associations between environmental and model parameters" a bit unusual. Remember, these hypothesis-testing statistics are testing the model parameters, not the data. Thus, prior to this type of approach, it should be verified that all individual models fit well and those for individuals that don't should be excluded.	We added information about the goodness of fit, based on RL-squared (the analogue of R-squared for SSLE): Logistic growth could achieve RL-squared of 98% for 78 of the 81 data. This was already a good fit. However, for most data the best-fit BP-model achieved RL-squared of 99.5%.
24	I know summary stats (and all values in the Supp. Material) are provided for the SSLE values but there is no indication of what SSLE value would constitute a good fit and what a poor fit in the present ms – I recommend including more detail on this, even though previously published elsewhere.	
25	If I were to square-root and backtransform SSLE, i.e. $e^{SQRT(SSLE)}$, would this equal the number of grams by which the curve deviated from the mass data across all measurements of an individual? I get a value between 1.03 and 1.5 across all individuals which seems like a very tight fit. It is my experience that some curves fit much worse than others, but this is not reflected here. Perhaps, it would help if the authors explained how the reader could gauge the fit of the model based on this value (a couple of plotted examples would help immensely).	We added an example of data (with a plot), where the fit was not particularly excellent, as at the end the bird started to shrink. Nevertheless, for the best-fit BP-model the maximal deviation was 0.37 g.
26	Once poor-fitting models are excluded and we can be sure the *minfl*/*mmax* ratio represents actual differences in shapes of growth curves (not just variation among parameter estimates – see my second point above), then we can use this to identify ecological associations with the *minfl*/*mmax* ratio.	As mentioned above (#12), the ratio could be used to represent actual differences in the shape, while the exponent-difference was more problematic.
27	The approaches used here are not common for the ornithological literature for two reasons:	We agree
28	i. Multiple comparisons of hypothesis tests: for the types of studies common in ornithological literature, moderate sample sizes abound and thus, as you note, multiple comparisons lead to higher probability of spurious significance (this is different when considering large order comparisons as common in the genetics/microbiology literature – see Lazzeroni, L. C., & Ray, A. (2012). The cost of large numbers of hypothesis tests on power, effect size and sample size. Molecular psychiatry, 17(1), 108-114.).	We partly agree, noting that we handled the issue of spurious significances by identifying significances with a particularly small p-value and by repeating the same comparisons under different aspects (different tests). If an association is repeatedly confirmed as (highly) significant, we deemed it as plausible that this association was not spurious.
29	ii. Multicollinearity: several predictor variables may be substantially correlated and so it is unclear the extent to which each is individually (or synergistically) important.	We agree, whence we pointed out only those associations that were confirmed in multiple tests. However, the identification of "really important" factors is rather a question about causation, which we avoided

		to discuss (more in #34).
30	While I believe that with careful treatment of the multiple comparisons, it is possible to state as you do: "In total, for each of the 11 environmental indicators we have applied six tests to each sample, resulting in 264 tests for significance (the entries in Tables 3 to 5). Amongst them, at most 19 outcomes would be spurious, recording a false significance (P-value 4.4% for a binomial distribution assuming a 5% chance for a false "significant"), while for our samples 61 outcomes were significant." Clearly 61/264 significant outcomes is beyond the realm of pure chance at a 0.05 level.	We partially agree, pointing out that more can be said (#28)
31	However, you cannot identify which of the 61 were the 19 spurious outcomes. I believe this means that you cannot further subdivide these results by saying: "light pollution (11 of 24 tests were significant), followed by the impervious area (10 significant tests) and the count of hatchlings (9 significant tests)" What if all 11 of the 24 tests of the light pollution predictor happened to be the spurious ones? Even if the chance of a spurious test was equal across all 264 tests (which given the various assumptions of the different tests and data themselves is unlikely), we would still expect 1 or 2 of the light pollution (19/264 x 24) tests to be spurious.	The paper is now more careful about this: Rather than summarizing the results in three tables, we explain them in more detail in the text, pointing out that e.g. for blue tits the association between light pollution and the ratio were highly significant and therefore most likely not spurious. Nevertheless, we stress that the main outcome of the paper was the proof-of-principle, that using the BP-model we could detect significant associations to the ratio (irrespective of the biological interpretation of this result)
32	I don't believe you can look any deeper with this analytical approach than saying that overall there are 3 times as many significant associations at the 0.05 level than would be expected by chance. This supports your statement: "We conclude that despite the small sample size the BP-model could detect significant statistical association between some local environmental variables and certain shape parameters of the BP-model, namely the ratio minfl/mmax and the exponent-difference b–a."	The conclusions are formulated more carefully, now. However, note the very low p-values for certain associations.
33	but renders the details presented in Tables 3-5 misleading and unreliable.	We removed the tables and explained the results in more detail in the text.
34	As for (ii) above, I imagine a correlation matrix across the 11 environmental variables would reveal strong associations (e.g. path vs human activity, sound vs road, etc..) and thus it is unclear which are important without adjustment for this.	Basically, the question of "important associations" is about causation, which we avoided to discuss, because the present data miss information about possibly confounding indicators, such as predation or food availability. Therefore, we point out in the conclusion that the main purpose of the paper was the proof of principle that the ratio may provide additional information.
35	Moreover, this suggests that the 264 significance tests aren't fully independent which would affect the likely rate of a spurious significance test.	We disagree with respect to the likely rate of "spurious": We tested this by means of three-dimensional multinormal distributions, where the first variable was uncorrelated to the second and third variables. Using 1000

		simulations of samples of given size and counting the spurious outcomes of the Spearman-Rank correlation test of the first variable with the second, then aside from the usual random fluctuations we did not discern a difference in the frequency between the simulations using the unit matrix as covariance matrix or a matrix with non-zero covariances between the second and third variable.
36	Finally, a related point is – how were nest-mates used in the analysis? If you have two chicks from the same nest box they are certainly not independent and thus have the potential to have more influence within the analytical group in which they fall.	In the revision we address this problem: Using resampling (deterministic selection of a typical bird from each nest and bootstrap simulations) we confirmed that for blue tits light pollution had a significant correlation with the ratio.
37	Finally, I believe the paper could be a bit easier to follow if revisions were made to the Tables and Figures. I found all the tables to be too detailed for the reader to get an immediate idea of what is important in each. These types of tables belong in supplementary data, with a figure or short table in the text.	As explained above most tables were removed and one was simplified. The relevant information was moved into the text, where it could be explained better.
38	Table 1 could go in supplementary info.	We removed the table – the needed information is stated in the text.
39	If the concerns about significance testing given above can be addressed, Tables 3 &4 could somehow be incorporated into Table 2 – thus the significance is reported using * = p< 0.05 etc..	We removed the tables and explained the outcomes in the text, pointing out, which outcomes most likely were not spurious.
40	and the medians from Table 4 could even be plotted in a figure.	The table has been removed
41	I certainly found it hard going to get much out of the current tabular presentation. I would also remove Figure 1.	Most tables and Figure 1 have been removed and the relevant information is presented more clearly in the text.
42	As indicated above it would be nice to see actual individuals labelled in Figure 2, even if fewer were plotted.	A plot was added that compares the mass data and the growth curves (see #25)
43	Figure 3 could be presented better – since this is one specific example, couldn't the mass data points be plotted with the curves? Even better would be a multipanel figure showing several fits.	The plot was intended to explain the different shape of growth curves without reference to concrete mass data. It has been put in a different context, Further, see the responses #25 and #42.
44	In Fig 4, the near-diagonal exponent pair stratification is an interesting one. Can you expand in the discussion about what this means (what are the physiological implications of having a near-diagonal exponent pair in the B-P curve)?	We note in the discussion that we started our research with this question. However, owing to the high variability of the exponent-difference (explained by a Figure) we could barely identify significant associations to explain the biological differences between near-diagonal and other birds.
44	I thank you for the opportunity to review this interesting work. I tried to be as detailed in my	OK, thanks for the extensive review

	comments as I could as I believe this method has much potential. However, please feel free to contact me if you have any further questions.	
46	I have also provided minor textual suggestions as comments in the ms I attached.	See below for the changes
	**Reviewer 1, comments in manuscript**	**Authors**
47	not a complete sentence	Changed
48	define as Blue Tit (BT) at first mention. For species names in the primary literature it is usual to spell the whole name throughout the text rather than abbreviating	The abbreviations BT and GT were replaced by the whole names
49	as per my suggested text edit, the three listed techniques in previous sentence are ways to fit and analyze curves rather than the curves themselves. Therefore the flow of this paragraph is disjunct and a bit misleading.	We moved the mention of these techniques to the methods section
50	put in Richards curve here too - as per your PeerJ article: Renner-Martin et al. 2018	Done
51	This is important but really breaks up the flow here so that eqn (2) is a sudden surprise. Can this text be moved to before or after discussion of eqn (2)?	Text has been changed
52	provide references for this in a few bird species	Text has been changed: It refers to the method, for which references have been added
53	It is unclear which papers you are referring to here. Ideally it would be one on the same dataset or at least study species you are using here. This text is also a broad statement - for example pooling data for curve fitting is very different from mixed-model analysis that uses random effects to control for individual effects.	General statements of this form were removed, as the introduction was streamlined and material was moved to the methods, where the explanations were more specific.
54	hence	Changed
55	just repeat the citations here	Done
56	don't use parenthesizes here	Done
57	I strongly believe more detail about this is needed here, even if it is simply an overview of how the method works	The methods section has been expanded additional subsections that provide more details
58	I would recommend moving the details about the tests - what was compared to what, using which test, from the Results to this part of the Methods.	Done
59	I strongly feel that more detail is needed here	The methods section has been expanded
60	could this indicate an unrepresentative fit for this individual?	Not necessarily, but in the present case yes (remark added to the text).
61	this would be better as a multipanel figure with several individuals plotted to show the better fit	See #41 and #42.
62	I don't find this very convincing as a justification of the fact that curves are well fitted for individuals. Perhaps I am not sure what you are trying to say. Given eqn (2) in the introduction it should be, by definition, the case that the ratio was related to asymptotic mass and inflection point. The fairly moderate rho values beg the question where the	We removed this text. However, there was no concern with data quality or poor fits.

	discrepancies arise in the data - is it measurement inaccuracy, differences between individuals or poor fitting?	
63	3 individuals does not sound like a very reliable sample	This was the reason of avoiding finer stratifications. We removed all discussions that required samples of less than six birds.
64	simple approach is more acccessible - your custom approach although theoretically more precise is less generally applicable by the common researcher. Help to have an R package to implement it.	The recommendation has been changed accordingly. As concerns an R-package, see #11.
	**Reviewer 2**	**Authors**
65	Very informative and didactic article on the subject of intense discussion in literature. My recommendation is that it be published, provided that the discussion with another papers in literature is improved.	OK
66	Below are some suggestions to improve the presentation of the article.	All suggestions were considered as follows
67	Introduction: Take care to define the acronym the first time it appears. For example, second paragraph, define the acronym BT.	Done; please note a shift in the material. The introduction is shorter and material has shifted to the methods
68	In the fifth paragraph (page 4), you can add the discussion with the article Fernandes et al. (2019) which also evaluated the allometric values (2/3, 1) and (3/4, 1), concluding that for mammals it is better use (3/4, 1).	Reference added
69	Sixth paragraph (page 4), Fernandes et al. (2020) also studied growth and solved the Bertalanffy-Putter differential equation, including adding the relationship between the "original" parameters with the three-parameter models. It is advisable to read this paper to assist in the discussion and presentation of the results.	Reference added
70	Computations and statistics: Statistical analyzes need to be better described. For example, to present the statistics of the used tests.	The statistics part of the methods has been expanded. However, as we removed several tables that were difficult to read (see above), we presented the outcomes of the tests in the text. In order to avoid a overloading of the text, we reported only the p-values of the key results and refrained from citing each outcome of the test-statistic.
71	Was the residue analysis performed after obtaining the estimates? Remember that this is the most important step in studies with regression models. Present (or even comment) on assumptions analysis	In methods/calibration we define the fit residues.
72	Authors should also assess the presence of heteroscedasticity. Looking at Figure 2, it seems quite plausible that the variances between the weighing days are heterogeneous (increasing over time).	Done ... and a distribution fit test supported the hypothesis of a lognormal distribution of mass
73	Data fitting: Why were used the logarithm of data? This transformation interferes in the parameter	We explain in methods/calibration that the logarithmic transformation stabilizes the

	estimates (Table 2).	variance
74	Was the inverse transformation done, to return with the parameters in the original scale?	We fitted log(BP-model) to log(data). As explained now in methods, in the original scale this is related to fitting a BP-model to the data by means of certain weighted least squares.
75	For example, was "b-a" calculated on the original scale or on the logarithmic scale?	As explained above, we always worked with the parameters in the original scale.
76	Best fit model: Even justifying that the focus is not on comparing models, it could present the value of at least R squared (R^2), for the reader to have an idea of the quality of the adjustment obtained.	Following this suggestion, we defined RL-squared as the analogue of R-squared for SSLE and discussed the fit. Thereby, we added a Figure about which RL-squared values could be obtained by 3-parameter models and we used RL-squared to explore the possible range of the ratio.
77	(Compreendi que o foco do artigo está em outros parâmetros, mas nada impede de discutir também os parâmetros de informações práticas, como peso adulto e abscissa (em dias) do ponto de inflexão. Veja a relação indicada por Fernandes et al. (2020).)	We shortly considered the asymptotic mass. However, to keep the paper focused on the ratio, which is the novel contribution of the paper, we did not go into details for other parameters.
78	Discussion and Conclusions: The conclusion of the article is based on the exponential difference parameters b-a and the Minfl/Mmax ratio. Therefore, they need to be better explained in the introduction. What is its biological importance?	We added a subsection in methods, where we presented biological interpretations of the parameters.
79	Why is it important for the reader to be concerned with these parameters and not simply adjust a three-parameter model?	We explain that each 3-parameter model has its own preassigned ratio and exponent-difference. However, this paper argues that the ratio may have a biological meaning, whence more flexible models are needed to study this claim. There are already flexible 4-parameter models, but we wanted to include all common 3-parameter models into the study, whence we used a 5-parameter model that generalizes them all.
80	Its results need to be discussed with articles already published in the literature about the subject. Otherwise it is not an discussion. Here are some suggestions for papers that can help in the discussion.<br>Fernandes et al. (2020), https://doi.org/10.28951/rbb.v38i3.457<br>Fernandes et al. (2019), https://doi.org/10.1590/S1678-3921.pab2019.v54.01162	Both references were added to the methods In order to enhance the readability, we decided to move all discussions on technical matters to the method, where the technicalities could be explained in more detail.
