# Supplementary material for: Bertalanffy-Pütter models for avian growth
Source: PLoS One. 2021 Apr 26;16(4):e0250515. doi: 10.1371/journal.pone.0250515 (PMC8075225; doi:10.1371/journal.pone.0250515)
Supplement: Attachment — Submitted filename: Response_to_Reviews_4.docx [file pone.0250515.s004.docx]

|  | **Editor** | **Authors** |
| --- | --- | --- |
| 01 | Thank you for submitting your manuscript to PLOS ONE. After careful consideration, we feel that it has merit but does not fully meet PLOS ONE’s publication criteria as it currently stands. Therefore, we invite you to submit a revised version of the manuscript that addresses the points raised during the review process. | Changes are described below |
| 02 | Please ensure that your manuscript meets PLOS ONE's style requirements, including those for file naming. The PLOS ONE style templates can be found at <https://journals.plos.org/plosone/s/file?id=wjVg/PLOSOne_formatting_sample_main_body.pdf>  <https://journals.plos.org/plosone/s/file?id=ba62/PLOSOne_formatting_sample_title_authors_affiliations.pdf> | done |
| 03 | Please amend the manuscript submission data (via Edit Submission) to include author Katharina Renner-Martin. | done |
| 04 | We note that Figure S1 n your submission contains satellite images which may be copyrighted. | Please note that we have resubmitted a completely new set of supporting information files. We removed the satellite image, as the site could be located easily from a Google search. |
|  | **Review 1** | **Authors** |
| 05 | This paper presents an application of the von Bertalanffy-Pütter growth curve for analyzing differences in growth parameters (chiefly the position of the inflection point) for passerines. There has been much discussion through the years since the initial work of Ricklefs on the most suitable growth curve to use for different species and applications. The strength of this approach lies in the ability to alter *minfl*/*mmax,* the position of the inflection point proportional to the extent of growth (most common parametric curves are limited to inflection occurring at a specific point in growth: e.g. 50% in the logistic model). | OK |
| 06 | I find the approach appealing. I agree with the argument that a flexible *minfl*/*mmax* ratio might have biological relevance in some instances of growth. I am also happy that curve-fitting was undertaken for each individual separately, as it is clear that the long tradition of pooling masses across individuals misses biologically important differences as it treats all individuals as equal. | OK |
| 07 | I found the paper well written and easy to follow | OK |
| 08 | but hard to evaluate because the data, although analyzed per individual, were never directly presented in that form: Fig 2 presents individual data but it is not clear which belong to which individual and thus to gauge individual growth trajectories. | We have obtained permission to publish the original data; they are supplied as supporting information |
| 09 | Corsini et al. only report mass at age 15 – near fledging, and they also say that nestlings were ringed (banded) at age 15d. (This begs the question how could they identify individual chicks prior to that point to be able to collect growth on individuals?). It would be beneficial to know how nestlings were weighed and the accuracy of the instruments used to weigh them. I presume this will be rectified when data are deposited but it makes evaluation of the method harder. | The nestlings were marked at the first weighing. |
| 10 | My second concern is that the conclusion at the end of the paper: “as noted in the introduction, for many common three-parameter models of avian literature the ratio is fixed (e.g. ratio 0.5 for logistic growth). We conclude that these more traditional three-parameter models are not suitable to explore and compare the shapes of growth curves”. I believe that this statement is too strong. Depending on the application of the growth curve a simpler curve may be just as appropriate and since it could be implemented in a well-developed analytical framework, such as mixed-effects models, it might be easier to use. | We modified our conclusion to point out its context. |
| 11 | I am very interested in the B-P approach but having to learn another program (Mathematica) just to implement it, is discouraging. From my experience, the majority of ornithologists use R for statistics and thus converting this code into an R package would greatly help foster its use by ornithologists. | The authors would support anyone interested in translating the m-codes into R. However, the authors are not natives of R. |
| 12 | One thing that is common is that as more parameters are estimated in a curve there is a greater potential for over-fitting, as changing one parameter can have a knock-on effect on another. I am familiar with this for the 4-parameter Richards curve and imagine that the B-P (4 or 5-parameters in eqn 1 in the paper) would suffer from the same issues. Thus, it is conceivable that different ratios of *minfl*/*mmax* with different parameters for c, p and q might achieve similar shaped curves. This would imply that just because the ratio of *minfl*/*mmax* has sufficiently different values for individuals or groups does not *necessarily* mean that it reflects the same differences in growth curve. I think what is needed is a figure (or even supplementary material) that shows there is consistency (or at least a limit to the range of variation) in curve shape across different ratios of *minfl*/*mmax*. | We have followed up this suggestion and presented more details of the following results in the paper: The observation of the reviewer is insofar true, as the exponent-differences and ratios *m_infl_*/*m_max_* varied widely amongst three-parameter models with a good fit to the data. However, for the ratios (but not for the exponent differences) the median of the ratios of good-fitting models was close to the ratio of the best-fit model. Thus, the ratios appear to have a biological meaning, while the exponent-differences remained somewhat evasive. We therefore removed the discussion of the exponent-difference (where owing to the high variability there were barely significant associations). Thank you for this valuable suggestion. |
| 13 | I also strongly believe that to truly show the B-P method is superior in the current application to two species of tits that the other 3-parameter curves (and/or Richards curve) need to be fit and compared to the B-P results. You could argue (perhaps by providing more details) that previous publications give support for the fact that B-P fit better than 3-parameter curves and so this is not necessary. I would still suggest at this stage to play it safe and demonstrate this (even if just in supplementary material) for the data set at hand. | Clearly the 5-parameter model necessarily has a better fit than any 3-parameter special case. However, as recommended in #15, the issue should not be the superiority of the BP-models over others, but its utility for the specific research question, the study of the ratio. Here the 5-parameter model is insofar superior, as the very topic would be beyond the reach of the 3-parameter models, which assume fixed ratios. We changed the paper accordingly. |
| 14 | It would also be nice to provide an indication of approximately how many data points are needed for a good fit: with a 4-parameter Richards curve clearly 6+ is desirable. The need for a large number of data points well-spaced through development clearly precludes using the method in many cases where data are more irregularly collected. | We used only data with eight points of time and required a certain regular scheme of weighing; all other (=most) data were discarded. As one PC could fit only 1-2 data per week, we reserved the available time for clearly comparable data, only. |
| 15 | Third, although stated in the 5th paragraph of the Introduction, the precise purpose of this paper [in how it differs from similar explorations for poultry (Kühleitner et al. 2019), poultry and dinosaurs (Brunner et al. 2019), and fish (Renner-Martin et al. 2018)] is currently not immediately forthcoming. I am interpreting it as: the need to show that differences in the ratio of *minfl*/*mmax* have ecological implications. If this is the case, please state it more strongly in the abstract and introduction (and discussion) rather than focusing on the B-P as a superior method. | We changed this accordingly: The issue of the paper is the consideration of the ratio and this requires a model that is flexible with respect to this ratio. |
| 16 | I also believe that the paper would benefit from reference to some of the following ornithological literature on growth curves that is missing from the Introduction: | We considered the literature as follows |
| 17 | Brisbin Jr., I.L., C.T. Collins, G.C. White and D.A. McCallum. 1987. A new paradigm for the analysis and interpretation of growth data: The shape of things to come. Auk 104: 552–554. | Cited in the results |
| 18 | Oswald, S.A., I.C.T. Nisbet, A. Chiaradia and J.M. Arnold. 2012. FlexParamCurve: R package for flexible fitting of nonlinear parametric curves. Methods in Ecology and Evolution 3: 1073–1077. | Cited in the methods |
| 19 | Remeŝ, V. and T.E. Martin. 2002. Environmental influences on the evolution of growth and developmental rates in passerines. Evolution 56: 2505–2518. | Cited in the methods |
| 20 | Sofaer, H.R., P.L. Chapman, T.S. Sillett and C.K. Ghalambor. 2013. Advantages of nonlinear mixed models for fitting avian growth curves. Journal of Avian Biology 44: 469–478. | Cited in the methods |
| 21 | Svagelj, W. S., Laich, A. G., & Quintana, F. (2019). Richards's equation and nonlinear mixed models applied to avian growth: why use them? Journal of Avian Biology, 50: e01864. | Cited in the methods |
| 22 | Zach, R. 1988. Growth-curve analysis: A critical reevaluation. Auk 105: 208–210. | Cited in the introduction |
| 23 | Fourth, I find the “shotgun” style statistical treatment in “Associations between environmental and model parameters” a bit unusual. Remember, these hypothesis-testing statistics are testing the model parameters, not the data. Thus, prior to this type of approach, it should be verified that all individual models fit well and those for individuals that don’t should be excluded. | We added information about the goodness of fit, based on RL-squared (the analogue of R-squared for SSLE): Logistic growth could achieve RL-squared of 98% for 78 of the 81 data. This was already a good fit. However, for most data the best-fit BP-model achieved RL-squared of 99.5%. |
| 24 | I know summary stats (and all values in the Supp. Material) are provided for the SSLE values but there is no indication of what SSLE value would constitute a good fit and what a poor fit in the present ms – I recommend including more detail on this, even though previously published elsewhere. |  |
| 25 | If I were to square-root and backtransform SSLE, i.e. eSQRT(SSLE), would this equal the number of grams by which the curve deviated from the mass data across all measurements of an individual? I get a value between 1.03 and 1.5 across all individuals which seems like a very tight fit. It is my experience that some curves fit much worse than others, but this is not reflected here. Perhaps, it would help if the authors explained how the reader could gauge the fit of the model based on this value (a couple of plotted examples would help immensely). | We added an example of data (with a plot), where the fit was not particularly excellent, as at the end the bird started to shrink. Nevertheless, for the best-fit BP-model the maximal deviation was 0.37 g. |
| 26 | Once poor-fitting models are excluded and we can be sure the *minfl*/*mmax* ratio represents actual differences in shapes of growth curves (not just variation among parameter estimates – see my second point above), then we can use this to identify ecological associations with the *minfl*/*mmax* ratio. | As mentioned above (#12), the ratio could be used to represent actual differences in the shape, while the exponent-difference was more problematic. |
| 27 | The approaches used here are not common for the ornithological literature for two reasons: | We agree |
| 28 | i. Multiple comparisons of hypothesis tests: for the types of studies common in ornithological literature, moderate sample sizes abound and thus, as you note, multiple comparisons lead to higher probability of spurious significance (this is different when considering large order comparisons as common in the genetics/microbiology literature – see Lazzeroni, L. C., & Ray, A. (2012). The cost of large numbers of hypothesis tests on power, effect size and sample size. Molecular psychiatry, 17(1), 108-114.). | We partly agree, noting that we handled the issue of spurious significances by identifying significances with a particularly small p-value and by repeating the same comparisons under different aspects (different tests). If an association is repeatedly confirmed as (highly) significant, we deemed it as plausible that this association was not spurious. |
| 29 | ii. Multicollinearity: several predictor variables may be substantially correlated and so it is unclear the extent to which each is individually (or synergistically) important. | We agree, whence we pointed out only those associations that were confirmed in multiple tests. However, the identification of “really important” factors is rather a question about causation, which we avoided to discuss (more in #34). |
| 30 | While I believe that with careful treatment of the multiple comparisons, it is possible to state as you do: “In total, for each of the 11 environmental indicators we have applied six tests to each sample, resulting in 264 tests for significance (the entries in Tables 3 to 5). Amongst them, at most 19 outcomes would be spurious, recording a false significance (P-value 4.4% for a binomial distribution assuming a 5% chance for a false “significant”), while for our samples 61 outcomes were significant.” Clearly 61/264 significant outcomes is beyond the realm of pure chance at a 0.05 level. | We partially agree, pointing out that more can be said (#28) |
| 31 | However, you cannot identify which of the 61 were the 19 spurious outcomes. I believe this means that you cannot further subdivide these results by saying: “light pollution (11 of 24 tests were significant), followed by the impervious area (10 significant tests) and the count of hatchlings (9 significant tests)” What if all 11 of the 24 tests of the light pollution predictor happened to be the spurious ones? Even if the chance of a spurious test was equal across all 264 tests (which given the various assumptions of the different tests and data themselves is unlikely), we would still expect 1 or 2 of the light pollution (19/264 x 24) tests to be spurious. | The paper is now more careful about this: Rather than summarizing the results in three tables, we explain them in more detail in the text, pointing out that e.g. for blue tits the association between light pollution and the ratio were highly significant and therefore most likely not spurious. Nevertheless, we stress that the main outcome of the paper was the proof-of-principle, that using the BP-model we could detect significant associations to the ratio (irrespective of the biological interpretation of this result) |
| 32 | I don’t believe you can look any deeper with this analytical approach than saying that overall there are 3 times as many significant associations at the 0.05 level than would be expected by chance. This supports your statement: “We conclude that despite the small sample size the BP-model could detect significant statistical association between some local environmental variables and certain shape parameters of the BP-model, namely the ratio minfl/mmax and the exponent-difference b–a.” | The conclusions are formulated more carefully, now. However, note the very low p-values for certain associations. |
| 33 | but renders the details presented in Tables 3-5 misleading and unreliable. | We removed the tables and explained the results in more detail in the text. |
| 34 | As for (ii) above, I imagine a correlation matrix across the 11 environmental variables would reveal strong associations (e.g. path vs human activity, sound vs road, etc..) and thus it is unclear which are important without adjustment for this. | Basically, the question of “important associations” is about causation, which we avoided to discuss, because the present data miss information about possibly confounding indicators, such as predation or food availability. Therefore, we point out in the conclusion that the main purpose of the paper was the proof of principle that the ratio may provide additional information. |
| 35 | Moreover, this suggests that the 264 significance tests aren’t fully independent which would affect the likely rate of a spurious significance test. | We disagree with respect to the likely rate of “spurious”: We tested this by means of three-dimensional multinormal distributions, where the first variable was uncorrelated to the second and third variables. Using 1000 simulations of samples of given size and counting the spurious outcomes of the Spearman-Rank correlation test of the first variable with the second, then aside from the usual random fluctuations we did not discern a difference in the frequency between the simulations using the unit matrix as covariance matrix or a matrix with non-zero covariances between the second and third variable. |
| 36 | Finally, a related point is – how were nest-mates used in the analysis? If you have two chicks from the same nest box they are certainly not independent and thus have the potential to have more influence within the analytical group in which they fall. | In the revision we address this problem: Using resampling (deterministic selection of a typical bird from each nest and bootstrap simulations) we confirmed that for blue tits light pollution had a significant correlation with the ratio. |
| 37 | Finally, I believe the paper could be a bit easier to follow if revisions were made to the Tables and Figures. I found all the tables to be too detailed for the reader to get an immediate idea of what is important in each. These types of tables belong in supplementary data, with a figure or short table in the text. | As explained above most tables were removed and one was simplified. The relevant information was moved into the text, where it could be explained better. |
| 38 | Table 1 could go in supplementary info. | We removed the table – the needed information is stated in the text. |
| 39 | If the concerns about significance testing given above can be addressed, Tables 3 &4 could somehow be incorporated into Table 2 – thus the significance is reported using * = p< 0.05 etc.. | We removed the tables and explained the outcomes in the text, pointing out, which outcomes most likely were not spurious. |
| 40 | and the medians from Table 4 could even be plotted in a figure. | The table has been removed |
| 41 | I certainly found it hard going to get much out of the current tabular presentation. I would also remove Figure 1. | Most tables and Figure 1 have been removed and the relevant information is presented more clearly in the text. |
| 42 | As indicated above it would be nice to see actual individuals labelled in Figure 2, even if fewer were plotted. | A plot was added that compares the mass data and the growth curves (see #25) |
| 43 | Figure 3 could be presented better – since this is one specific example, couldn’t the mass data points be plotted with the curves? Even better would be a multipanel figure showing several fits. | The plot was intended to explain the different shape of growth curves without reference to concrete mass data. It has been put in a different context, Further, see the responses #25 and #42. |
| 44 | In Fig 4, the near-diagonal exponent pair stratification is an interesting one. Can you expand in the discussion about what this means (what are the physiological implications of having a near-diagonal exponent pair in the B-P curve)? | We note in the discussion that we started our research with this question. However, owing to the high variability of the exponent-difference (explained by a Figure) we could barely identify significant associations to explain the biological differences between near-diagonal and other birds. |
| 44 | I thank you for the opportunity to review this interesting work. I tried to be as detailed in my comments as I could as I believe this method has much potential. However, please feel free to contact me if you have any further questions. | OK, thanks for the extensive review |
| 46 | I have also provided minor textual suggestions as comments in the ms I attached. | See below for the changes |
|  | **Reviewer 1, comments in manuscript** | **Authors** |
| 47 | not a complete sentence | Changed |
| 48 | define as Blue Tit (BT) at first mention. For species names in the primary literature it is usual to spell the whole name throughout the text rather than abbreviating | The abbreviations BT and GT were replaced by the whole names |
| 49 | as per my suggested text edit, the three listed techniques in previous sentence are ways to fit and analyze curves rather than the curves themselves. Therefore the flow of this paragraph is disjunct and a bit misleading. | We moved the mention of these techniques to the methods section |
| 50 | put in Richards curve here too - as per your PeerJ article: Renner-Martin et al. 2018 | Done |
| 51 | This is important but really breaks up the flow here so that eqn (2) is a sudden surprise. Can this text be moved to before or after discussion of eqn (2)? | Text has been changed |
| 52 | provide references for this in a few bird species | Text has been changed: It refers to the method, for which references have been added |
| 53 | It is unclear which papers you are referring to here. Ideally it would be one on the same dataset or at least study species you are using here. This text is also a broad statement - for example pooling data for curve fitting is very different from mixed-model analysis that uses random effects to control for individual effects. | General statements of this form were removed, as the introduction was streamlined and material was moved to the methods, where the explanations were more specific. |
| 54 | hence | Changed |
| 55 | just repeat the citations here | Done |
| 56 | don't use parenthesizes here | Done |
| 57 | I strongly believe more detail about this is needed here, even if it is simply an overview of how the method works | The methods section has been expanded additional subsections that provide more details |
| 58 | I would recommend moving the details about the tests - what was compared to what, using which test, from the Results to this part of the Methods. | Done |
| 59 | I strongly feel that more detail is needed here | The methods section has been expanded |
| 60 | could this indicate an unrepresentative fit for this individual? | Not necessarily, but in the present case yes (remark added to the text). |
| 61 | this would be better as a multipanel figure with several individuals plotted to show the better fit | See #41 and #42. |
| 62 | I don't find this very convincing as a justification of the fact that curves are well fitted for individuals. Perhaps I am not sure what you are trying to say. Given eqn (2) in the introduction it should be, by definition, the case that the ratio was related to asymptotic mass and inflection point. The fairly moderate rho values beg the question where the discrepancies arise in the data - is it measurement inaccuracy, differences between individuals or poor fitting? | We removed this text. However, there was no concern with data quality or poor fits. |
| 63 | 3 individuals does not sound like a very reliable sample | This was the reason of avoiding finer stratifications. We removed all discussions that required samples of less than six birds. |
| 64 | simple approach is more acccessible - your custom approach although theoretically more precise is less generally applicable by the common researcher. Help to have an R package to implement it. | The recommendation has been changed accordingly. As concerns an R-package, see #11. |
|  | **Reviewer 2** | **Authors** |
| 65 | Very informative and didactic article on the subject of intense discussion in literature. My recommendation is that it be published, provided that the discussion with another papers in literature is improved. | OK |
| 66 | Below are some suggestions to improve the presentation of the article. | All suggestions were considered as follows |
| 67 | Introduction: Take care to define the acronym the first time it appears. For example, second paragraph, define the acronym BT. | Done; please note a shift in the material. The introduction is shorter and material has shifted to the methods |
| 68 | In the fifth paragraph (page 4), you can add the discussion with the article Fernandes et al. (2019) which also evaluated the allometric values (2/3, 1) and (3/4, 1), concluding that for mammals it is better use (3/4, 1). | Reference added |
| 69 | Sixth paragraph (page 4), Fernandes et al. (2020) also studied growth and solved the Bertalanffy-Putter differential equation, including adding the relationship between the "original" parameters with the three-parameter models. It is advisable to read this paper to assist in the discussion and presentation of the results. | Reference added |
| 70 | Computations and statistics: Statistical analyzes need to be better described. For example, to present the statistics of the used tests. | The statistics part of the methods has been expanded. However, as we removed several tables that were difficult to read (see above), we presented the outcomes of the tests in the text. In order to avoid a overloading of the text, we reported only the p-values of the key results and refrained from citing each outcome of the test-statistic. |
| 71 | Was the residue analysis performed after obtaining the estimates? Remember that this is the most important step in studies with regression models. Present (or even comment) on assumptions analysis | In methods/calibration we define the fit residues. |
| 72 | Authors should also assess the presence of heteroscedasticity. Looking at Figure 2, it seems quite plausible that the variances between the weighing days are heterogeneous (increasing over time). | Done ... and a distribution fit test supported the hypothesis of a lognormal distribution of mass |
| 73 | Data fitting: Why were used the logarithm of data? This transformation interferes in the parameter estimates (Table 2). | We explain in methods/calibration that the logarithmic transformation stabilizes the variance |
| 74 | Was the inverse transformation done, to return with the parameters in the original scale? | We fitted log(BP-model) to log(data). As explained now in methods, in the original scale this is related to fitting a BP-model to the data by means of certain weighted least squares. |
| 75 | For example, was "b-a" calculated on the original scale or on the logarithmic scale? | As explained above, we always worked with the parameters in the original scale. |
| 76 | Best fit model: Even justifying that the focus is not on comparing models, it could present the value of at least R squared (R²), for the reader to have an idea of the quality of the adjustment obtained. | Following this suggestion, we defined RL-squared as the analogue of R-squared for SSLE and discussed the fit. Thereby, we added a Figure about which RL-squared values could be obtained by 3-parameter models and we used RL-squared to explore the possible range of the ratio. |
| 77 | (Compreendi que o foco do artigo está em outros parâmetros, mas nada impede de discutir também os parâmetros de informações práticas, como peso adulto e abscissa (em dias) do ponto de inflexão. Veja a relação indicada por Fernandes et al. (2020).) | We shortly considered the asymptotic mass. However, to keep the paper focused on the ratio, which is the novel contribution of the paper, we did not go into details for other parameters. |
| 78 | Discussion and Conclusions: The conclusion of the article is based on the exponential difference parameters b-a and the Minfl/Mmax ratio. Therefore, they need to be better explained in the introduction. What is its biological importance? | We added a subsection in methods, where we presented biological interpretations of the parameters. |
| 79 | Why is it important for the reader to be concerned with these parameters and not simply adjust a three-parameter model? | We explain that each 3-parameter model has its own preassigned ratio and exponent-difference. However, this paper argues that the ratio may have a biological meaning, whence more flexible models are needed to study this claim. There are already flexible 4-parameter models, but we wanted to include all common 3-parameter models into the study, whence we used a 5-parameter model that generalizes them all. |
| 80 | Its results need to be discussed with articles already published in the literature about the subject. Otherwise it is not an discussion. Here are some suggestions for papers that can help in the discussion.  Fernandes et al. (2020), https://doi.org/10.28951/rbb.v38i3.457  Fernandes et al. (2019), https://doi.org/10.1590/S1678-3921.pab2019.v54.01162 | Both references were added to the methods In order to enhance the readability, we decided to move all discussions on technical matters to the method, where the technicalities could be explained in more detail. |
