# Supplementary material for: Bertalanffy-Pütter models for avian growth
Source: PLoS One. 2021 Apr 26;16(4):e0250515. doi: 10.1371/journal.pone.0250515 (PMC8075225; doi:10.1371/journal.pone.0250515)
Supplement: Attachment — Submitted filename: RebuttalLetter_Revision2.docx [file pone.0250515.s006.docx]

| **No** | **Reviewer #1:** | **Authors** |
| --- | --- | --- |
| 1 | I applaud the authors for their revisions. These really help bring out the impact of the study. | This was also due to your efforts. |
| 2 | Although there have been many excellent language revisions, I still believe that the language can be more precise and briefer in many places. Since PLoS ONE does not copy edit following acceptance, I have taken the liberty to provide very detailed language suggestions throughout on a marked-up copy of the ms that I have uploaded here. Feel free to use these as you see fit. | Thank you very much for these detailed comments. We considered all comments in the manuscript copy, but do not mention them in this file.. |
| 3 | There are still a few issues I have with the ms but these are with presentation not content (the authors have done a great job of toning down the ms in areas where the original became speculative). The main problem is that the end of Methods and Results are too long and arduous and the Discussion is too short. Since much of this is wording/formatting I have provided detailed comments on this below: | Following your suggestions, we streamlined the text and reshuffled material. |
| 4 | 1) METHODS/RESULTS ARE VERY LONG. The following sections of the Methods and Results are unnecessary long and deter the reader: | In part, the methods are long, because we had moved introductory material into this section to streamline the introduction. |
| 5 | a. Remove lines 129-136: this has already been covered adequately on lines 84-93. | We removed the material from the earlier lines |
| 6 | b. Lines 143-151: This paragraph is a bit confusing in a methods section as it is talking about your results. I suggest two possible solutions: either Talk about it in more pilot-study or theoretical terms. e.g. "If we compare two BP-growth curves...." or move it to the Discussion. I also suggest moving this paragraph up to be before the line starting "This paper explores.." (Lines 127) | We left only the sentence that we study the ratio. |
| 7 | c. Lines 213-220: suggest move this entire paragraph to Supplementary Info since has already been used in other published studies | We removed it, except for comments that responded to reviewer 2 |
| 8 | d. Lines 221-223: Since this is a single example, it would be good to show more examples for several reasons: 1) to give the reader more confidence that the fit is good across all individuals and that the error shown is consistent - perhaps quote the range of error across all study birds; 2) to show that the curve does better for shapes that are quite different from standard (the example shown looks visually a lot like a logistic curve). I believe that Fig 1 could be eliminated (since it is clear in the text that a flexible inflection point is what you are using) and Fig 3 could be expanded to include several difference examples. | We removed this example and plotted a similar example (the two worst-fitting growth curves) in the result |
| 9 | e. Line 234-238: a bit tangental to the study in hand. It is reasonable to expect fitting to fail for 5 parameters on 8 datapoints – suggest removal | removed |
| 10 | f. Line 277: since this is so high (43,000 models), there needs to be some indication of why - were exponent-pairs assessed to the nearest three significant figures? Or does this include models used to explore the environmental factors. How many possible models were there, how big was the grid-space? | This was the count of grid-points: In the discussion we use it to illustrate a possible speed-up for the computation of the ratio |
| 11 | g. Line 279-284: this is unusual phrasing because usually you describe which tests you did on which data, rather than what appears to be more of a philosophy behind the statistical approach. Since specific tests were matched to the specific analyses that used them below, I'd delete this paragraph | We now discuss only the material relevant for testing the ratio. |
| 12 | h. Lines 301-337. This needs substantial reduction and rephrasing. On reading the first paragraph of the Discussion, I finally understood the approach. I would suggest moving the first paragraph of the Discussion here, and just expanding it by putting in the bare necessary details from the existing method text. Shorter the better. | Done |
| 13 | i. Lines 353-359: This is a good initial approach for when first examining the data. However, since it is one bird, I don't think it should take up a whole paragraph of the Results of the paper. Instead, justify it by saying it captured the correct shape and was only deficient in the height of the asymptote so you didn't exclude it. | We shortened this to one sentence. |
| 14 | 2) RESULTS ARE HARD TO FOLLOW. The sections: “Identification of the ratio from the shape”, “Correlations with the ratio”, “Further associations with the ratio for blue tits” and “Related Issue” need to be shortened and renamed. I would suggest signposting from the analysis roadmap provided in response to my comment “h” above by giving easier to follow names that represent each of the 3 analytical approaches used [a) correlation tests, b) location tests for the shape-parameter (ratio) of birds from clearly distinct environments, and c) location tests of the environmental parameters for birds with clearly distinct shapes of the growth curves]. Also, substantial deletions to each of these sections is necessary to avoid losing the reader in unnecessary detail: | We changed the subsection-structure and followed the roadmap in the subsection statistical analysis of the ratio. |
| 15 | a. Lines 387-422. This is very long and unclear. On the attached marked-up ms, I have provided considerable edits to make this brief and to-the-point. Please address this. | This material is now shortened and part of the subsection on ratios for the best and nearly best fitting growth curves. |
| 16 | b. Lines 385-386, 430-434. These are Methods and should not be in the Results section. | Removed and shortened, respectively |
| 17 | c. Lines 453-476: This is Methods+Results mixed. It is also a justification of the effect not being an artifact of experimental design. Thus, this should go in Supplementary Info and the at Line 456 say: 'However, this was found not to be the case (Supplementary Info)". If indeed that is the conclusion - it was quite confusing. | This is now part of the subsection on testing bias. It is shorter and explains only one approach (selecting one typical bird from each nest). |
| 18 | d. Lines 344-345. 481-486 – delete – this is already in the Methods. | done |
| 19 | e. Lines 417-422: This is unclear. I think you have something here but it is not clear what. I think you are saying that the median of all estimated values was often close to the best fit value - suggesting even if you only did a single fit you might be in the right ballpark.... is this correct. If so, please rephrase. | This is now formulated more clearly (and other comments distracting from this outcome were removed) |
| 20 | f. Line 440-524: consider putting all rhos and their signif levels in a table - would shorten up the text (I know you took some of this out of tables on my request but now that you have shortened and refocused this, a single table with these results would be appropriate and help shorten the text). | Done: Table 2 collects the results for ISA and light pollution for blue tits in detail (all are significant or highly significant). All other significant outcomes are merely summarized in the text. |
| 21 | 3) SIGNIFICANCE TESTING. Line 440 onwards. The reporting of p-values in the text as % is non-standard and confusing. Please put these in as proportions – thus p=4.4% would be p=0.044. As you say in the Response to Reviewers, you should concentrate on the highly significant results. A p of 0.04 is so close to non-significant that given the number of tests, it should not be reported. Please consider removing the reporting of the rho and p for p > 0.01 from these, as there isn't sufficient confidence in these results (this will also make it easier to have a table for all these results, including only the highly significant ones). Also, it is not clear why the term “stochastically” is used. Please explain better in text. | Done, see also the above comment #20. |
| 22 | 4) CORRELATIONS BETWEEN PREDICTORS. Lines 506-524: This is a welcome discussion but I believe the magnitude of the correlation rather than the significance should be the focus here. It is possible to get very significant correlations that are not really very substantial (from many repeated measures or little variability). Thus, focus on magnitude. Since these are environmental factors I would argue that anything over rho = 0.6 (equivalent of an R2 of ~0.4) would be a fairly important correlation, but some would argue anything rho = 0.5 (R2 of 0.25) and above. Also, this would be better in a table showing the correlation matrix among variables. This could be in the Supplementary Info to shorten up the paper. Please note “Related Issue” is not an informative title. | We shortened this discussion in the style of comment #20. It is now a motivating example, why we supplemented the estimate of the count of spurious correlations by simulations that accounted for the dependencies between the environmental indicators. |
| 23 | 5) DISCUSSION TOO BRIEF. Once all the methodological info is removed, the discussion is somewhat lacking. Please comment on what you have achieved – the discovery that the ratio is important. What does this mean going forward? How might ornithologists use this – do they need to, can they given the sample size restrictions? Certainly more discussion is needed. | We wrote a new discussion |
| 24 | a. Lines 587-598: This is only of importance for someone wanting to repeat your study to look at whether the ratio gives a biological signal. Instead, think of the limitations of avian ecologists - fewer data points , but likely more birds of a single species and fewer environmental factors. Given these considerations a better discussion paragraph could be made- the model has some relevance here - as greater sample sizes and perhaps more data points in species that take longer to develop (e.g. 20-40 days) could be better | We discuss this issue |
| 25 | 6) COMPUTER PROCESSING TIME FOR FITTING. LINE 595 and response to comment 14. A data fit of 1 week of computer time per chick is outrageously slow and intense and would not appeal to many ornithologists. However, I believe that this is because you were validating the methods and your grid-search is fine-grained - to the 3rd significant figure. Commenting on this in the paper and indicating that the median ratio was fine and all values were within X units of the median suggests that a much coarser resolution could be used and that would give more realistic processing times - e.g if accuracy to 0.5 units was required how long would this take? For someone to want to fit these models something down to 1h per bird is more realistic (but still slow – considering it takes less than a second for the logistic curve). Perhaps, future research should explore results sensitivity to cutting the parameter search space and perhaps even explore different fitting methods (this could certainly be suggested). | We recommend generalized logistic growth for computing the ratio. This is a four-parameter special case of the BP-model and it can be optimized in less than one hour. We added a few lies on the Akaike information criterion to compare this model with the general BP-model. |
| 26 | A FEW OTHER SMALL POINTS: | Considered |
| 27 | Line 161 – Birds were marked: how? Using felt markers? | Colors and clipping |
| 28 | Line 165- it is more usual convention to call hatching date "Day 0" | Actually it is day 1 for our data. |
| 29 | Line 188 – NDVI - provide scale of resolution (1m2, 10m2, 1km2?) | For all geo-information indicators a radius of 100 m with a pixel-resolution of 20 m was used |
| 30 | Line 222: add (~5% of maximum mass) to indicate what 0.37g means in your case. | Different birds were now considered; we mention their maximal observed mass. |
| 31 | Line 348: Figure 3 only shows 1 bird so is not sufficient evidence. I would suggest that the fact that 94% of birds were normally-distributed then the underlying distribution is normal, and stating this is fine - the others could simply be random sampling errors. | We changed the figure and plot the worst fitting growth curves for each species. |
| 32 | Fig 4: these near diagonals actually look like pairs that are insensitive to the value of "a". This seems true of much of the data - it seems as though if b is low then a can take on any value, if b<6 then a <1, and otherwise as b increases a reduces. | This impression of “insensitivity” was caused by the small number of mass-at-age data per bird. Daily measurements would have resulted in clearly distinct regions of near-optimality for each bird. |
| 33 | Thank you again for the opportunity to review your interesting work. | Thank you for the very detailed comments which helped to focus our paper on the issues of interest for ornithologists. |
| **No** | **Reviewer #2:** | **Authors** |
| 34 | The article has changed considerably from the first version submitted. | OK |
| 35 | Now the text is clearer and more objective and can be published. | OK |
| 36 | I only recommend careful with the statement of line 205, because when stating that the adjustment is made between the logarithm of the model and the logarithm of the data, it is assumed that the error was multiplicative in the original Putter model (BP-model). | We added a comment that the choice of calibration depends on the purpose of the fitted model (referring to an innovative calibration in epidemiology, where the counts with the highest uncertainty weigh least). |
| 37 | All suggestions from this reviewer were accepted and the paper was considerably improved. | OK |
| 38 | The results are better discussed and therefore the paper has merit to be published. | OK |
| 39 | I didn't check the layout question according to the journal's rules. | Thank you again for your insightful comments. |
